# Sterically demanding macrocyclic Eu(iii) complexes for selective recognition of phosphate and real-time monitoring of enzymatically generated adenosine monophosphate†

**DOI:** 10.1039/d1sc05377a

**Published:** 2022-02-11

**Authors:** Samantha E. Bodman, Colum Breen, Sam Kirkland, Simon Wheeler, Erin Robertson, Felix Plasser, Stephen J. Butler

**Affiliations:** Department of Chemistry, Loughborough University Epinal Way Loughborough LE11 3TU UK s.j.butler@lboro.ac.uk

## Abstract

The design of molecular receptors that bind and sense anions in biologically relevant aqueous solutions is a key challenge in supramolecular chemistry. The recognition of inorganic phosphate is particularly challenging because of its high hydration energy and pH dependent speciation. Adenosine monophosphate (AMP) represents a valuable but elusive target for supramolecular detection because of its structural similarity to the more negatively charged anions, ATP and ADP. We report two new macrocyclic Eu(iii) receptors capable of selectively sensing inorganic phosphate and AMP in water. The receptors contain a sterically demanding 8-(benzyloxy)quinoline pendant arm that coordinates to the metal centre, creating a binding pocket suitable for phosphate and AMP, whilst excluding potentially interfering chelating anions, in particular ATP, bicarbonate and lactate. The sensing selectivity of our Eu(iii) receptors follows the order AMP > ADP > ATP, which represents a reversal of the order of selectivity observed for most reported nucleoside phosphate receptors. We have exploited the unique host–guest induced changes in emission intensity and lifetime for the detection of inorganic phosphate in human serum samples, and for monitoring the enzymatic production of AMP in real-time.

## Abstract 2

We present two new europium-based anion receptors that selectively bind to inorganic phosphate and AMP in aqueous media. Their sensing selectivity follows the order AMP > ADP > ATP, representing a reversal of the selectivity order observed for most nucleoside phosphate receptors.

## Graphical abstract

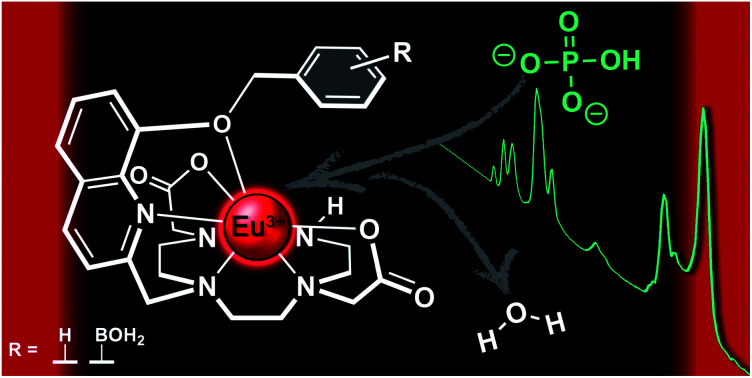

## Introduction

In the field of anion recognition, the development of receptors that bind a target anion selectively in water remains a major challenge, due to the high hydration energies of anions, their range of geometries and pH sensitivities.^1–3^ The recognition of inorganic phosphate is particularly challenging^4^ because it is highly hydrated (
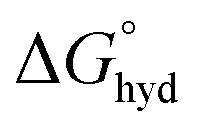
 = −465 kJ mol^−1^) and exists in the forms H_2_PO_4_^−^ and HPO_4_^2−^ in a 1 : 1 ratio in water at pH 7.0.^5^ Adenosine monophosphate (AMP) represents a valuable yet very difficult target for supramolecular detection because of its similarities with the more negatively charged anions, ATP and ADP.^6–8^

Inorganic phosphate plays important roles in the functions of muscles and nerves and skeletal mineralisation. The level of phosphate in human blood is maintained within a relatively narrow range (0.8–1.45 mM),^9^ similar to lactate levels (0.5–1.0 mM) and substantially lower than bicarbonate (23–29 mM) and chloride (98–115 mM).^10,11^ Nucleoside phosphate anions (*e.g.* ATP, ADP, AMP) are present in very low concentrations in human blood and extracellular fluid,^12^ whereas their intracellular concentrations are higher.^13–15^

Numerous diseases cause imbalances in the concentration of inorganic phosphate, hence the development of sensors for its quantitative determination is of great importance for medical diagnosis and disease management.^6,16^ For instance, high phosphate levels in blood are linked with kidney disease and vascular calcification, increasing risk of stroke.^17,18^ For biomedical applications, receptors that target phosphate in blood should exhibit modest affinity for the anion, permitting its detection in the millimolar range whilst displaying excellent selectivity over other anions, particularly bicarbonate and lactate. Examples of receptors that fulfil this criteria and can operate in 100% water are rare.^7,10,19^ Anslyn and co-workers achieved high phosphate selectivity by the careful positioning of ammonium/guanidinium groups around a preorganised receptor with a central Cu(ii) ion, overcoming the strong interactions between phosphate and its hydration sphere.^20^

AMP is a key metabolite that regulates energy homeostasis and signal transduction. AMP is a product of several enzyme reactions including aminoacyl tRNA synthetases and phosphodiesterases, many of which are misregulated during disease conditions.^21–23^ Therefore, monitoring the activities of these enzymes *in vitro* is a critical step in the discovery of new potent enzyme inhibitors and activators. Currently, there is a lack of low-cost methods for real-time analysis of phosphodiesterase and tRNA synthetase reactions that are amenable to high throughput screening, which inhibits progress in the discovery of new inhibitors of these enzymes.^24–26^ Existing assays involving radiolabelled substrates present health hazards and require special facilities, whereas enzyme-coupled assays (*e.g.* AMP-Glo) rely on elaborate multi-step procedures to quantify the AMP using luciferase/luciferin chemiluminescence. Whilst these end-point assays have been crucial in profiling enzyme activities, they are not suitable for direct real-time analysis which makes the determination of enzyme kinetics more difficult.

A synthetic receptor that can discriminate AMP from ATP and other nucleoside phosphate anions would be extremely useful as a bioassay tool for *in vitro* enzyme reaction monitoring and high throughput screening of inhibitors. However, receptors capable of targeting AMP selectively are very rare.^27–29^ Indeed, most reported nucleoside phosphate receptors exhibit a selectivity profile that is governed predominantly by electrostatic interactions and thus follows the order ATP > ADP > AMP.^6,8,30–32^ Such receptors are not suitable for detecting AMP during enzyme reactions, especially in the presence of ATP.

Lanthanide(iii)-based receptors are well suited for sensing phosphate species in water, due to the inherent affinity of Ln(iii) ions for hard oxyanions.^10,11,33,34^ By incorporating a strongly absorbing chromophore (antenna) within the ligand structure, emissive lanthanide(iii) probes can be created that offer unique photophysical properties ideal for sensing in biological assays,^35–39^ including: (1) long luminescence lifetimes that enable time-resolved measurements to eliminate short-lived autofluorescence; (2) line-like emission spectra which enables accurate ratiometric analyses; and (3) a luminescence response that is fast and sensitive, offering the high spatial and temporal resolution required for biological imaging applications.

Despite their valuable optical properties, creating Ln(iii)-based receptors for phosphate or AMP is very difficult because other oxyanions (*e.g.* ATP, bicarbonate and lactate) can compete for coordination to the Ln(iii) centre.^10,11,34^ To the best of our knowledge, there are no examples of macrocyclic Ln(iii) complexes that can bind phosphate or AMP with high selectivity. Recently, Pierre and co-workers developed a series of acyclic tripodal Eu(iii) complexes with an open coordination site that binds 2 or 3 molecules of phosphate with high affinity (log *β* = 11.34) and very good selectivity, although the binding of nucleoside phosphate anions was not reported.^40^ Using a series of related tripodal Gd(iii) complexes,^41,42^ Pierre determined the affinity and selectivity for phosphate to be a function of the stability of the Gd(iii) complex and the basicity of the anion. Albrecht and co-workers developed a double stranded Eu(iii) helicate that exhibits a highly specific emission enhancement upon binding AMP.^49^ We recently developed a class of thermodynamically stable macrocyclic Eu(iii) receptors that present a cavity with sufficient flexibility for the binding of ADP^43,44^ or ATP^45^ in a bidentate manner, facilitated by reversible de-coordination of one of the pendant quinoline arms.^46^ These receptors showed comparatively weaker binding to AMP and phosphate.

**Fig. 1:**
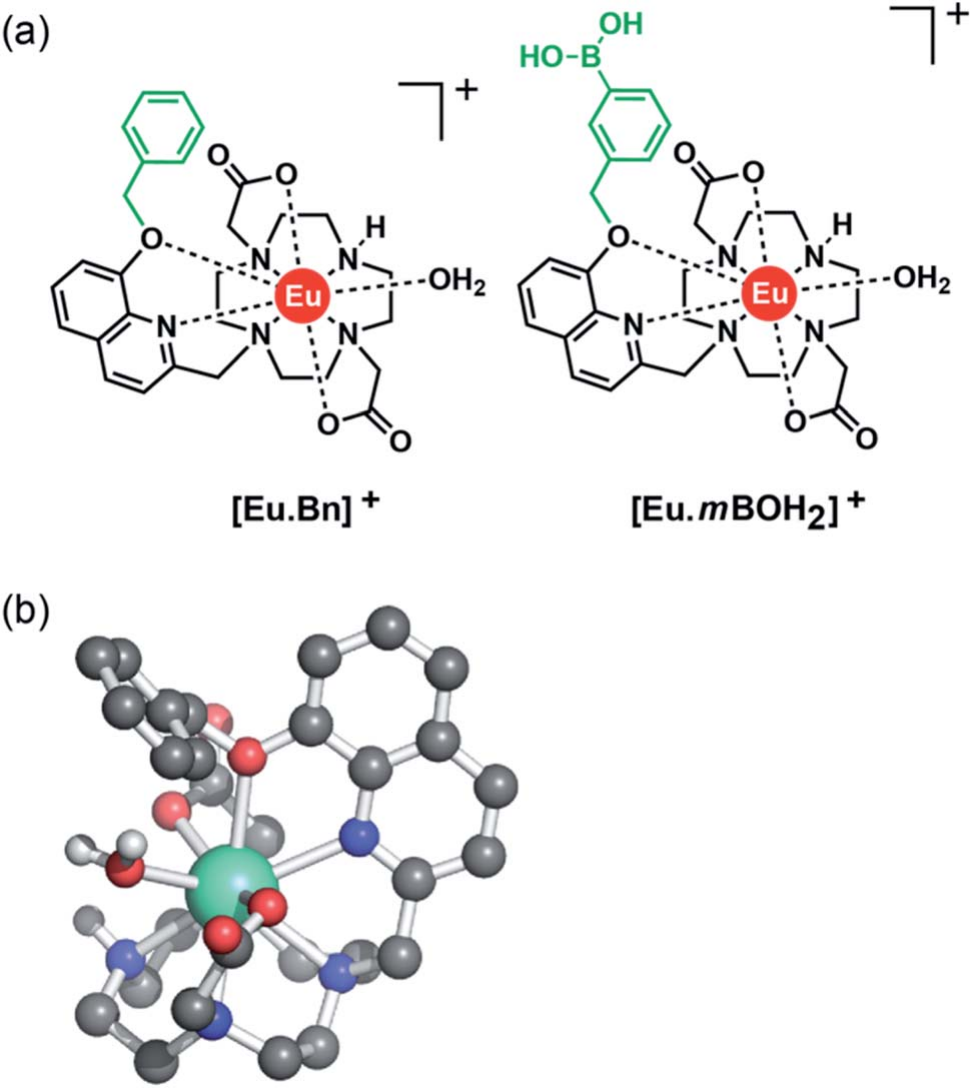
(a) Structures of cationic Eu(iii) complexes designed to bind phosphate and AMP in water, (b) DFT optimised geometry of [Eu·Bn]^+^ showing the anion binding site occupied by a single water molecule.

With this background in mind, we set out to develop two macrocyclic Eu(iii) complexes (Fig. 1) designed to selectively recognise phosphate and AMP in aqueous solution, whilst displaying minimal interference from other biological anions, particularly ATP, bicarbonate and lactate. Each Eu(iii) complex features a bulky 8-(benzyloxy)quinoline pendant arm that coordinates the Eu(iii) ion in a bidentate manner, offering a single coordination site for the monodentate binding of phosphate and AMP. Other oxyanions that prefer a bidentate binding mode, including ATP, bicarbonate and lactate,^11,46,47^ do not bind to the receptors due to the steric hindrance imposed by the surrounding ligand, which blocks the ‘axial’ coordination site. In attempt to gain multisite recognition of AMP, we incorporated a phenylboronic acid motif within the ligand structure of [Eu·*m*BOH_2_]^+^. The unique ability of [Eu·*m*BOH_2_]^+^ to recognise AMP over ATP was utilised to develop a new bioassay tool for monitoring the enzymatic production of AMP in real-time.

## Results and discussion

### Molecular design

Two cationic Eu(iii) complexes were designed to provide sufficient host rigidity and steric hindrance around the metal centre to permit monodentate binding of phosphate and AMP (Fig. 1), whilst preventing the chelation of common oxyanion interferents, in particular ATP, bicarbonate and lactate. Initially, [Eu·Bn]^+^ (Fig. 1) was designed based on a cyclen scaffold incorporating an 8-(benzyloxy)quinoline pendant arm to coordinate the central Eu(iii) ion in a bidentate manner. The opposite secondary amine of the cyclen scaffold is unfunctionalized, offering a single phosphate binding site at the Eu(iii) centre, occupied by a single water molecule in aqueous solution. Indeed, density functional theory (DFT) computations of the mono-aqua complex [Eu·Bn]^+^ (Fig. 1b) showed that the quinoline arm occupied the ‘axial’ site covering the top of the receptor. The bound quinoline moiety also serves as an antenna for the efficient sensitization of Eu(iii) emission.^48^ Two carboxylate donors are positioned either side of the quinoline arm, creating an octadentate ligand that ensures high thermodynamic stability of the resulting Eu(iii) complex. The overall charge of the Eu(iii) complex is +1, to complement the negative charge of inorganic phosphate and AMP at neutral pH.

A boronic acid moiety was introduced within the phenyl ring of receptor [Eu·*m*BOH_2_]^+^ to interact reversibly with the *cis*-diol moiety of the ribose sugar of AMP. Phenylboronic acids are well known to form reversible interactions with 1,2-diols in aqueous media and have been utilized in a range of sensors for carbohydrates with great success.^50,51^ We envisaged that incorporation of a *meta*-boronic acid would enable multisite recognition of AMP, involving coordination of the phosphate group to the Eu(iii) centre and simultaneous interaction between boronate ester and the ribose sugar of AMP.

### Synthesis

The synthesis of [Eu·Bn]^+^ and [Eu·*m*BOH_2_]^+^ was achieved using similar methods and full details are provided in the ESI (Schemes S1 and S2†). The synthesis commenced with *O*-alkylation of 8-hydroxyquinoline-2-carbaldehyde with either benzyl bromide or *meta*-iodobenzyl bromide, to give the corresponding 8-(benzyloxy)quinoline derivatives. Reduction of the aldehyde using sodium borohydride gave the primary alcohol and subsequent mesylation afforded the methanesulfonate esters, which were used directly to alkylate one of the secondary amines of DO2A-*tert*-butyl ester to give the macrocycles bearing a single quinoline pendant arm. The *tert*-butyl ester groups were removed using trifluoroacetic acid, followed by purification of the benzyloxyquinoline ligand by reverse-phase HPLC. Addition of EuCl_3_·6H_2_O in water at pH 7–8 afforded the target complex [Eu·Bn]^+^ and the benzyl iodide complex [Eu·*m*I]^+^ after reverse-phase HPLC purification. The boronic acid moiety was introduced as a pinacol boronic ester in the final borylation step, to avoid undue exposure to acidic conditions during the ligand synthesis. Accordingly, reaction of the benzyl iodide complex [Eu·*m*I]^+^ with bis(pinacolato)diboron using Pd(dppf)Cl_2_ catalyst in DMSO gave access to the target boronic acid-functionalised complex [Eu·*m*BOH_2_]^+^ after purification by reverse-phase HPLC.

### Photophysical analysis

**Fig. 2:**
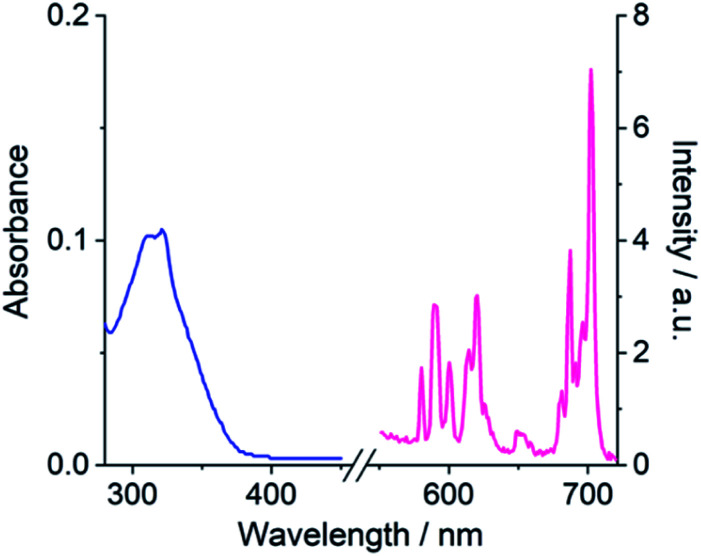
Absorption (blue) and emission (pink) spectrum of [Eu·*m*BOH_2_]^+^ (5 μM) in 10 mM HEPES at pH 7.0 and 295 K, *λ*_exc_ = 322 nm.

Table 1 presents photophysical data for the Eu(iii) complexes measured in 10 mM HEPES at pH 7.0. The UV-Vis absorption spectra of the complexes are similar and display a broad band centred at 322 nm, tailing out to around 370 nm (Fig. 2 and S1†). The emission spectra of [Eu·Bn]^+^, and [Eu·*m*BOH_2_]^+^ were very similar (Fig. 2 and S2†), displaying two distinguishable components in the Δ*J* = 1 (585–600 nm) emission band and three components in the Δ*J* = 2 (605–630 nm) band. Analysis of the intensity ratio of the Δ*J* = 2/Δ*J* = 1 emission bands gave a value of approximately 1.4 in each case, indicating that each complex adopts a conformation of low symmetry in aqueous solution. The quantum yields of the metal-centred luminescence of [Eu·Bn]^+^ and [Eu·*m*BOH_2_]^+^ were around 1.5% and emission lifetimes in H_2_O were approximately 0.19 ms and at least 30% longer in D_2_O (Table 1). The number of coordinated water molecules, *q*, was determined to be one for both complexes.

**Table 1:** Photophysical data for Eu(iii) complexes measured in 10 mM HEPES at pH 7.0

Complex	*λ*_max_/nm	*ε*/M^−1^ cm^−1^	*τ*_H_2_O_ a/ms	*τ*_D_2_O_ a/ms	*q* b	*Φ*_em_ c/%
[Eu·Bn]^+^	322	2900	0.192	0.252	1.2	1.7
[Eu·*m*BOH_2_]^+^	322	2700	0.199	0.283	1.5	1.5

a Emission lifetimes were measured at pH 7.0.

b Values of hydration state *q* (±20%) were derived using literature methods.^53^

c Quantum yields were measured using quinine sulfate in H_2_SO_4_ as standard (*Φ*_em_ = 60%),^54^ errors in *Φ*_em_ are ±15%.

Analysis of the pH dependence of the emission spectra of the Eu(iii) complexes in water revealed that the ratio of the Δ*J* = 2/Δ*J* = 1 emission bands increased by approximately 2-fold as the pH was increased from 4 to 11 (Fig. S3†). By fitting the change in the intensity ratio Δ*J* = 2/Δ*J* = 1 with pH, a p*K*_a_ value of 8.86 ± 0.02 was estimated for [Eu·*m*BOH_2_]^+^, comparable with the p*K*_a_ of the phenylboronic acid/boronate ester couple of 8.9.^52^ For [Eu·Bn]^+^ a slightly higher p*K*_a_ value of 9.65 ± 0.08 was determined. Importantly, for both complexes the intensity ratio Δ*J* = 2/Δ*J* = 1 varied by less than 4% in the physiologically relevant pH range 6.0–7.5, auguring well for their application as sensors in biological media.

### Selective emission enhancement for phosphate

**Fig. 3:**
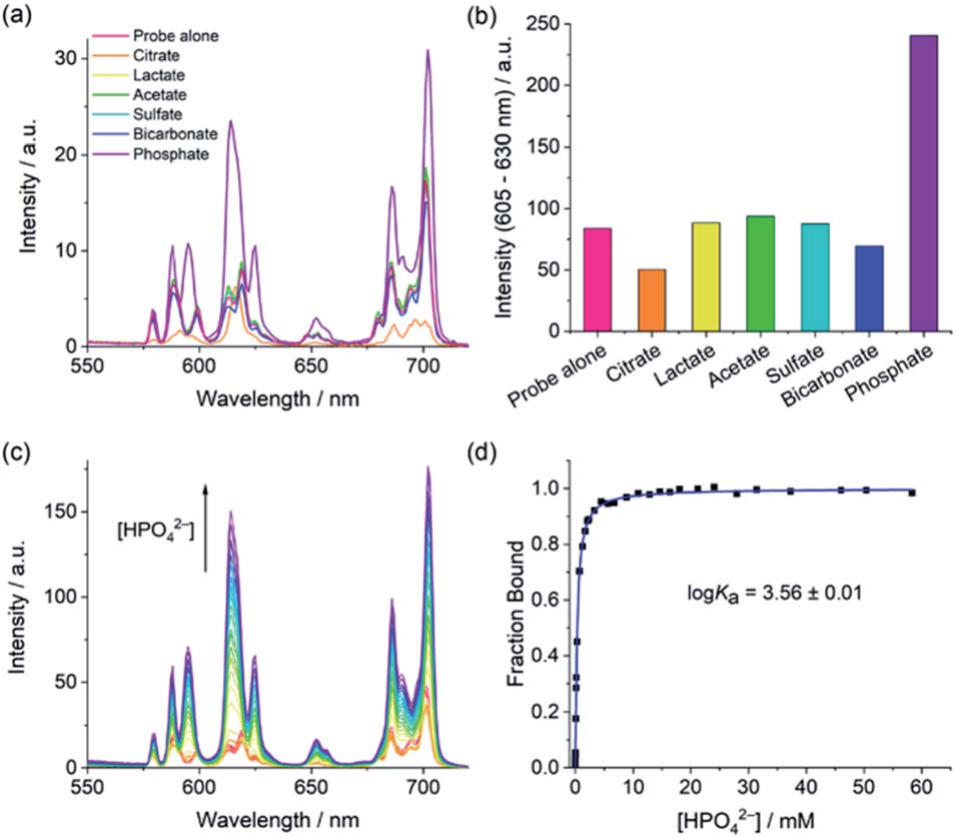
Selective emission response of [Eu·Bn]^+^ for phosphate. (a and b) Emission enhancement of [Eu·Bn]^+^ (5 μM) with phosphate (1 mM) compared with citrate, lactate, acetate, sulfate and bicarbonate (1 mM each); (c) variation in emission spectra of [Eu·Bn]^+^ upon incremental addition of phosphate; (d) plot of fraction bound (determined from Δ*J* = 2/Δ*J* = 1 intensity ratio) *versus* phosphate concentration, showing the fit to a 1 : 1 binding isotherm. Measured in 10 mM HEPES at pH 7.0 and 295 K, *λ*_exc_ = 322 nm.

The anion binding capabilities of the Eu(iii) complexes were assessed by recording their emission spectra in the presence of different biologically relevant anions 10 mM HEPES at pH 7.0. Initially, we wished to determine whether the receptors have selectivity toward phosphate over other anions present in blood serum, with a particular focus on bicarbonate and lactate due to their tendency to bind many Ln(iii)-complexes.^11,34^ Addition of 1 mM phosphate to [Eu·Bn]^+^ and [Eu·*m*BOH_2_]^+^ resulted in an approximate 3-fold enhancement in overall Eu(iii) emission intensity and pronounced changes in spectral shape (Fig. 3 and S4†), characterised by a large increase in the hypersensitive Δ*J* = 2 band centred at 614 nm, indicating binding of phosphate and displacement of the coordinated water. Pleasingly, the emission spectra remained essentially unchanged in the presence of bicarbonate, lactate, sulfate and acetate, confirming that these anions do not bind to the receptors. The complete lack of binding of bicarbonate and lactate is attributed to the steric demand imposed by the 8-(benzyloxy)quinoline pendant arm.

The binding of phosphate to [Eu·Bn]^+^ and [Eu·*m*BOH_2_]^+^ gave rise to a unique emission spectral signature, comprising an approximate 3-fold increase in the Δ*J* = 2 band and a pronounced shoulder at 625 nm, an increase in the Δ*J* = 2/Δ*J* = 1 intensity ratio from 1.4 to 2.4, and the emergence of two signals within the Δ*J* = 1 band at 588 and 595 nm. The changes in fine structure of the Δ*J* = 1 emission band is a consequence of a change in crystal field around the Eu(iii) ion when phosphate is bound, specifically involving a change in magnitude and sign of the second order crystal field parameter, *B*_0_^2^.^55^ Luminescence lifetimes measured in H_2_O and D_2_O in the presence of phosphate revealed that *q* = 0 (Table S1†), confirming that phosphate displaces the bound water molecule.

The only other anion tested that induced a change in emission was citrate, which caused a reduction in the overall emission intensity for both complexes, most notably within the Δ*J* = 1 and Δ*J* = 4 bands (Fig. 3a, S4 and S5†), indicating a common binding mode for this anion. Considering that citrate is well known to chelate Ln(iii) centres^47,56^ and given the decrease in emission intensity observed for each complex here, we propose that chelation of citrate causes decoordination of the quinoline arm, leading to less efficient sensitisation of Eu(iii) and less effective shielding of the metal centre from surrounding water.‡‡It was not possible to determine the binding affinity of citrate due to the small changes in emission intensity that took place, which prevented accurate fitting of the data, see Fig. S5.† Indeed, emission lifetimes in the presence of citrate revealed that *q* = 0.3–0.7 (Table S1†), indicating partial hydration in the citrate-receptor complexes.

### Establishing the sensing selectivity order AMP > ADP > ATP

**Fig. 4:**
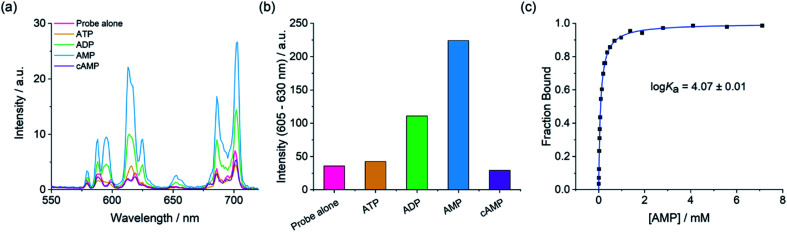
Selective emission enhancement of [Eu·*m*BOH_2_]^+^ for AMP over ATP, ADP and cAMP. (a) Change in emission spectra of [Eu·*m*BOH_2_]^+^ (5 μM) in the presence of 1 mM AMP over ATP, ADP and cAMP. (b) Change in intensity of the Δ*J* = 2 emission band of [Eu·*m*BOH_2_]^+^ (5 μM) with AMP, ATP, ADP and cAMP (1 mM each); (c) plot of fraction bound (determined from Δ*J* = 2/Δ*J* = 1 intensity ratio) *versus* AMP concentration, showing the fit to a 1 : 1 binding isotherm. Measured in 10 mM HEPES at pH 7.0 and 295 K, *λ*_exc_ = 322 nm.

We next examined the ability of the receptors to discriminate between the nucleotide phosphate anions, AMP, ADP, ATP and cAMP. Addition of 1 mM AMP to [Eu·*m*BOH_2_]^+^ induced an approximate 4-fold enhancement in overall Eu(iii) emission intensity and a striking 6-fold increase in the Δ*J* = 2 band (Fig. 4). In sharp contrast, ATP induced a negligible increase in emission and cAMP did not bind to the receptor at all. The emission enhancement caused by ADP was less than half that observed for AMP. A similar sensing pattern was observed for [Eu·Bn]^+^ (Fig. S6†), although the level of discrimination for AMP over ADP and ATP was slightly less pronounced.

Crucially, the sensing selectivity of the receptors follows the order AMP > ADP > ATP, which is opposite to that observed for most nucleoside phosphate receptors^6–8^ where the binding is governed predominantly by electrostatic interactions and thus favours ATP and ADP over AMP. We have previously shown that ATP and ADP prefer to adopt a bidentate binding mode in more conformationally flexible macrocyclic Ln(iii) receptors.^46^ However, we propose that such bidentate binding to [Eu·*m*BOH_2_]^+^ and [Eu·Bn]^+^ is not possible due to the increased rigidity of the anion binding site, combined with the large steric demand imposed by the bulky pendant arm. These structural features enable the sensing selectively to be tuned towards the less highly charged anion, AMP.

Adding AMP to [Eu·*m*BOH_2_]^+^ induced similar emission spectral shape changes to those observed with phosphate, indicating a similar conformation for the AMP-receptor complexes. Emission lifetimes in the presence of AMP revealed that *q* = 0, consistent with displacement of the coordinated water molecule (Table S1†). In the presence of ATP a *q* value of 1.0 and 0.5 was found for [Eu·Bn]^+^–ATP and [Eu·*m*BOH_2_]^+^–ATP, respectively, consistent with the very small increases in emission intensity observed for this anion.

Evidence in support of a 1 : 1 binding stoichiometry between the Eu(iii) receptors and phosphate, AMP and ADP was given by high resolution mass spectrometry. We observed major signals for the singly charged host–guest species [Eu·Bn + H_2_PO_4_ + Na]^+^, [Eu·Bn + AMP + 2Na]^+^ and [Eu·Bn + ADP + 3Na]^+^ (Fig. S7†), whereas no signals corresponding to other binding stoichiometries were observed in electrospray mass spectrometry. A 1 : 1 host–guest species was also observed between [Eu·BOH_2_]^+^ and phosphate, although the signal was weaker (Fig. S8†), attributed to the tendency of boronic acid-functionalised molecules to undergo dehydration at elevated temperatures in the mass spectrometer.^57^

### Determination of binding affinities

Apparent binding constants were determined for the Eu(iii) receptors and phosphate, AMP and ADP in aqueous buffer at pH 7.0, by following the change in the intensity ratio of the Δ*J* = 2/Δ*J* = 1 bands as a function of anion concentration, followed by fitting of the data to a 1 : 1 binding model (Table 2, Fig. 4 and S9†). Receptor [Eu·Bn]^+^ showed slightly stronger binding to inorganic phosphate compared with [Eu·*m*BOH_2_]^+^. Both receptors showed stronger binding to AMP compared with phosphate, with the boronic acid complex [Eu·*m*BOH_2_]^+^ showing a slightly higher level of selectivity. The binding of both receptors to ADP was slightly weaker compared with AMP. It was not possible to estimate binding constants between ATP and the receptors due to the minor changes in emission intensity that took place (Fig. 4 and S9†), which prevented accurate fitting of the data.

**Table 2:** Apparent binding constants (log *K*_a_) determined for Eu(iii) complexes with phosphate, AMP and ADP in 10 mM HEPES buffer at pH 7.0 and 295 K

Anion	log *K*_a_	
	[Eu·Bn]^+^	[Eu·*m*BOH_2_]^+^
HPO_4_^2−^	3.56 ± 0.01	3.31 ± 0.04
AMP	4.02 ± 0.01	4.07 ± 0.01
ADP	3.65 ± 0.01	3.84 ± 0.01
ATP	n.d.^a^	n.d.^a^

a Value not determined due to only minor changes in emission intensity preventing accurate data fitting.

Although the receptors showed only modest differences in affinity for AMP and ADP, the key objective in this work was to selectively detect AMP over the more highly charged anions ATP and ADP. The much larger luminescence enhancement observed for AMP (Fig. 4 and S8†) can be attributed to the formation of tight AMP-receptor complexes (log *K*_a_ ∼ 4.0) wherein the organic portion of the anion shields the Eu(iii) ion effectively from water molecules in the first and second coordination spheres. Assuming a similar monodentate binding mode for ADP (based on the similar spectral shape to AMP), the significantly lower emission with ADP is ascribed to a greater degree of luminescence quenching brought by water in the second coordination sphere of the ADP-receptor complexes, possibly due to the presence of the second, unbound phosphate group of ADP in close proximity to the Eu(iii) ion. The high sensitivity of [Eu·*m*BOH_2_]^+^ for AMP was demonstrated by the linear increase in the emission over the 0–200 μM range (Fig. S10†).

### DFT optimised structures of host-anion binding

**Fig. 5:**
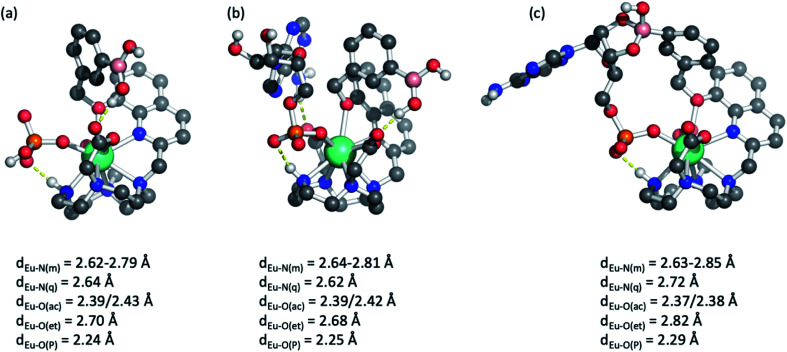
Molecular geometries of [Eu·*m*BOH_2_]^+^ bound to (a) inorganic phosphate and AMP in (b) a monodentate and (c) a bidentate manner involving formation of a cyclic boronic ester. Bond distances of Eu(iii) to donor atoms are shown: N(m) – macrocycle, N(q) – quinoline, O(ac) – acetate, O(et) – ether, O(P) – phosphate.

Further insight into the binding modes of inorganic phosphate and AMP was gained by DFT optimised structures of the host–guest complexes. Using the crystallographic data of a structurally related Eu(iii) complex previously reported by us,^46^ we modelled the Eu(iii) complexes bound to a single water molecule, then to phosphate and AMP. Selected geometries are shown in Fig. 5 and S11,† binding energies are reported in Table S2.† As expected, complexes [Eu·Bn]^+^ and [Eu·*m*BOH_2_]^+^ possess notable binding affinity for H_2_O (104 and 107 kJ mol^−1^, respectively). In comparison, binding energies for mono-coordinated phosphate and AMP were significantly higher, in the range of 340–375 kJ mol^−1^, highlighting that the complexes possess a strong affinity for these anions. Notably, binding of AMP was consistently higher by approximately 25 kJ mol^−1^ compared with phosphate, in agreement with experimentally determined binding constants (Table 2).

Molecular geometries of the host–guest complexes of [Eu·*m*BOH_2_]^+^ with phosphate or AMP bound in monodentate manner are shown in Fig. 5a and b. Notably, the phosphate oxygen binds tightly to Eu(iii) with a bond distance of around 2.25 Å in both cases, and a hydrogen bond between the N–H group in the macrocycle and phosphate oxygen is formed. In addition, AMP forms a hydrogen bond with one of the acetate pendant arms, as shown in the background of Fig. 5b. This additional hydrogen bond may explain the enhanced binding affinity of AMP compared with phosphate. Similar coordination geometries were found for [Eu·Bn]^+^ (Fig. S11†).

Finally, we explored the possibility of the multisite recognition of AMP by complex [Eu·*m*BOH_2_]^+^ involving coordination of the phosphate group to the Eu(iii) centre and simultaneous interaction between boronic ester and the ribose sugar of AMP. Formation of the cyclic boronic ester was indeed possible (Fig. 5c). Overall, a similar geometry to the monodentate binding mode is obtained (Fig. 5b); however, the bond lengths of Eu(iii) to the phosphate oxygen, the quinoline nitrogen, and the ether oxygen are slightly elongated indicating some ring strain. Computations indicated that in this geometry the formation of the cyclic boronic ester is endothermic with respect to the monodentate complex (Fig. 5b); however, the existence of such a complex with a reasonable geometry makes it plausible that both proposed AMP-receptor complexes exist in aqueous solution.

### Detection of phosphate in human serum

Our ultimate goal is to apply our Eu(iii) receptors for analysis of real biological samples, which are much more complex than the buffered aqueous solutions discussed so far. To this end, we conducted competitive titration experiments initially confirming the ability of [Eu·Bn]^+^ to detect phosphate in the presence of physiological levels of bicarbonate (27 mM) and human serum albumin (HSA 0.4 mM). Titrations revealed that bicarbonate and HSA have little or no impact on the receptor's affinity and selectivity for phosphate (Fig. S12 and S13†).

Next, we evaluated the ability of [Eu·Bn]^+^ to sense phosphate in an aqueous medium that mimics extracellular fluid, based on a modified Krebs saline solution commonly used in cell and tissue culture experiments. The solution contains NaCl (145 mM), KCl (5 mM), CaCl_2_ (2.5 mM), MgCl_2_ (1.5 mM), NaHCO_3_ (27 mM), Na_2_SO_4_ (0.5 mM), sodium lactate (1.0 mM), sodium citrate (0.15 mM), glucose (5.5 mM) and HSA (0.4 mM), buffered in 10 mM HEPES at pH 7.0. Pleasingly, addition of phosphate to [Eu·Bn]^+^ under these conditions gave rise to a large emission enhancement especially within the Δ*J* = 2 band (Fig. S14†). An apparent binding constant of log *K*_a_ = 3.32 was determined, slightly lower than that found for phosphate in aqueous buffer (Table 2). Importantly, the emission response of [Eu·Bn]^+^ is tuned to the millimolar concentration range, auguring well for analysis of phosphate levels in blood serum.

**Fig. 6:**
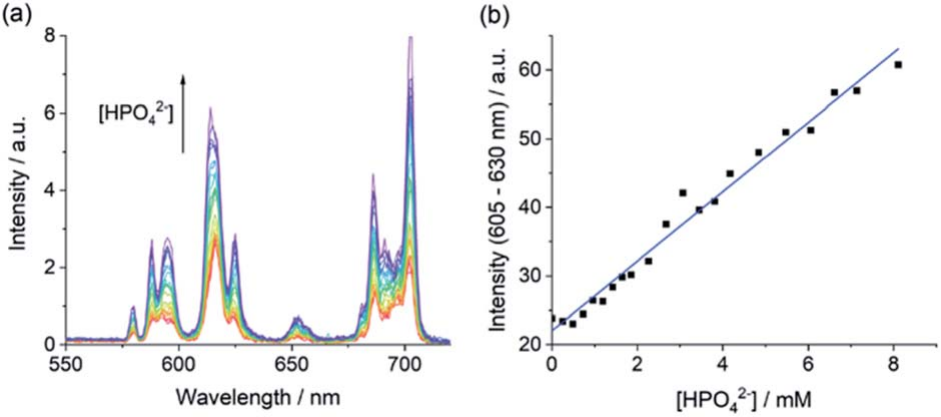
Measurement of phosphate in human serum. (a) Variation in time-resolved emission spectrum of [Eu·Bn]^+^ (20 μM) and (b) linear increase in time-resolved emission of the Δ*J* = 2 band upon addition of phosphate in human serum. Measured at pH 7.0 and 295 K, *λ*_exc_ = 322 nm, integration time = 60–400 μs.

Encouraged by the competition experiments, we demonstrated the utility of [Eu·Bn]^+^ for determining phosphate concentrations in real-life blood serum samples. [Eu·Bn]^+^ was incubated in human serum for five minutes before recording the time-resolved luminescence spectrum, effectively eliminating background fluorescence from the biological sample (Fig. S15†). Analysis of the emission spectra compared with the calibration plot (Fig. S14†) allowed an estimate of the phosphate concentration of 0.8 mM in the serum sample, in accordance with the literature.^7^ Subsequent addition of phosphate to the serum sample gave rise to a linear increase in the Δ*J* = 2 emission band over the phosphate range 1 to 8 mM (Fig. 6b). This highlights the potential of [Eu·Bn]^+^ for estimating unknown phosphate concentrations in real-life blood serum samples, potentially enabling elevated levels to be identified for the diagnosis and treatment of kidney disease.

### Monitoring the enzymatic production of AMP

The selective emission enhancement of the boronic-acid receptor [Eu·*m*BOH_2_]^+^ for AMP compared with its negligible response to ATP and cAMP offers powerful potential for real-time monitoring of enzymatic reactions that produce AMP. Two important enzyme classes that represent attractive druggable targets are cyclic nucleotide phosphodiesterases and aminoacyl tRNA synthetases.^58,59^ PDEs catalyse the conversion of cAMP to AMP and are thus important in cellular signalling.^60,61^ Aminoacyl tRNA synthetases play a crucial role in protein synthesis, catalysing the transfer of amino acids onto tRNA, enabling subsequent transfer of the amino acid onto a growing polypeptide chain, converting ATP into AMP and pyrophosphate (PPi) in the process.^62^ Current assay methods available to monitor the activity of these enzymes in real-time are poorly suited to high-throughput screening approaches for the identification of potent enzyme inhibitors.

**Fig. 7:**
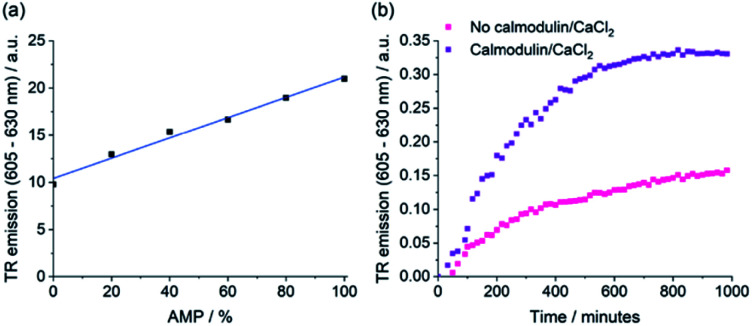
Real-time monitoring of phosphodiesterase (PDE) activity. (a) Simulation of the PDE reaction, recording the time-resolved emission of [Eu·*m*BOH_2_]^+^ with increasing % AMP. Conditions: 100 μM cAMP + AMP, 5 mM MgCl_2_, 5 μM [Eu·*m*BOH_2_]^+^, 10 mM HEPES at pH 7.0. (b) Real-time monitoring of the PDE-catalysed conversion of cAMP to AMP using the time-resolved emission of [Eu·*m*BOH_2_]^+^, comparing the change in rate of reaction upon activation with calmodulin (2 μM) and CaCl_2_ (60 μM). Conditions: 200 μM cAMP, 4 μL PDE (0.1 mg mL^−1^), 5 mM MgCl_2_, 10 μM [Eu·*m*BOH_2_]^+^, 10 mM HEPES at pH 7.0, *λ*_exc_ = 292–366 nm, *λ*_em_ = 615–625 nm, integration time = 60–400 μs.

We decided to simulate phosphodiesterase and tRNA synthetase reactions using a microplate reader. To simulate the phosphodiesterase reaction, we added differing ratios of AMP/cAMP to a 384-well plate containing receptor [Eu·*m*BOH_2_]^+^. Upon increasing the AMP/cAMP ratio we observed a linear increase in time-resolved emission intensity (Fig. 7 and S16†), indicating that the luminescence signal could be directly correlated to phosphodiesterase activity. The receptor displayed excellent signal-to-noise over the AMP concentration range 0–200 μM (Fig. S16†). Simulation of the tRNA synthetase reaction was performed in a similar manner using a more complex aqueous medium containing [Eu·*m*BOH_2_]^+^, KCl (10 mM), MgCl_2_ (5 mM), DTT (1 mM), and all 20 natural amino acids (100 μM each). Pleasingly, increasing the (AMP + PPi)/ATP ratio gave rise to a linear increase in time-resolved luminescence (Fig. S17†). Our receptor shows high sensitivity towards AMP (0–30 μM), even in the presence of ATP and all 20 natural amino acids. We verified the stability of the emission signal towards AMP (and other nucleoside phosphates) over a 4 hours incubation period (Fig. S18†).

To demonstrate the practical application of the receptor in bioassays, we incubated cAMP with a PDE enzyme in the presence of [Eu·*m*BOH_2_]^+^ and monitored the increase in time-resolved luminescence during the course of the reaction (Fig. 7b and S19†). Upon addition of the phosphodiesterase activators calmodulin and CaCl_2_, a faster rate of [Eu·*m*BOH_2_]^+^ emission increase and hence enzyme reaction rate was observed. Thus, we can monitor phosphodiesterase activity in real-time, by following the production of AMP. Our supramolecular assay offers advantages over existing enzyme-coupled assays, including obviating the need for expensive antibodies and labelled substrates, or isolation/washing steps, thereby delivering a potential solution for high throughput screening of inhibitors and kinetic analysis of AMP-generating enzymes.

## Conclusion

We present a new class of cationic Eu(iii) complexes capable of selectively recognising inorganic phosphate and AMP in aqueous solution. Each receptor contains a sterically demanding 8-(benzyloxy)quinoline pendant arm that coordinates the central Eu(iii) ion, blocking the ‘axial’ site covering the top the receptor. The binding cavity is thus suitable for monodentate binding of phosphate and AMP, whilst inhibiting access of commonly interfering and chelating oxyanions, including ATP, bicarbonate and lactate. The excellent selectivity of [Eu·Bn]^+^ for phosphate was exploited for measuring phosphate levels in real-life human serum samples.

A major advantage of our Eu(iii) receptors is their high discriminatory ability towards AMP over ATP. The sensing selectivity follows the order AMP > ADP > ATP, with AMP inducing a striking enhancement in emission intensity compared with only minor emission changes caused by ATP. Thus, by increasing the steric demand at the Eu(iii) centre and creating a relatively rigid anion binding site, we have reversed the sensing selectivity profile observed for most reported nucleoside phosphate receptors, where the binding is governed by electrostatic interactions and follows the order ATP > ADP > AMP.

The AMP-selective response of [Eu·*m*BOH_2_]^+^ was exploited to develop an *in vitro* assay for monitoring phosphodiesterase activity in real-time, by directly monitoring AMP production in a convenient increase-in-luminescence format. Our Eu(iii) complex offers advantages over existing assays for AMP-generating enzymes, removing the need for (1) expensive labelled substrates or equipment, (2) antibodies or coupled enzymes, (3) isolation/purification steps, or (4) the need to remove ATP prior to measurement. The receptor design principles established here will inform future development of receptors with enhanced selectivity for AMP over ADP, enabling quantification of AMP in more complex biological media.

## Data availability

ESI† is available, including compound synthesis and characterisation, spectrometric analysis and computational modelling of host-anion binding interactions.

## Author contributions

S. E. B. and C. B. carried out synthesis and photophysical analysis of the host molecules and their anion binding properties, with support from S. W. and S. K. and E. R. F. P performed computational modelling. S. J. B. conceived and designed the research programme. The paper was written by S. J. B. with contributions from S. E. B., S. W. and F. P.

## Conflicts of interest

There are no conflicts to declare.

## Supplementary Material

SC-013-D1SC05377A-s001
