# Supplementary material for: Sterically demanding macrocyclic Eu(iii) complexes for selective recognition of phosphate and real-time monitoring of enzymatically generated adenosine monophosphate
Source: Chem Sci. 2022 Feb 11;13(12):3386–94. doi: 10.1039/d1sc05377a (PMC8943852; doi:10.1039/d1sc05377a)
Supplement: SC-013-D1SC05377A-s001 [file SC-013-D1SC05377A-s001.pdf]

Electronic Supplementary Material (ESI) for Chemical Science.
This journal is © The Royal Society of Chemistry 2022

Supporting Information

Sterically Demanding Macrocyclic Eu(III) Complexes for Selective Recognition of Phosphate and Real-time Monitoring of Enzymatically Generated Adenosine Monophosphate

Samantha E. Bodman[a], Colum Breen[a], Sam Kirkland[a], Simon Wheeler[a], Erin Robertson[a], Felix Plasser[a] and Stephen J. Butler*[a]

General Considerations

Luminescence experiments

Luminescence spectra were recorded on a Camlin Photonics luminescence spectrometer with FluoroSENS version 3.4.7.2024 software. Emission spectra were obtained using a 40 µL or 100 µL Hellma Analytics quartz cuvette (Art no. 111-10-K-40). Excitation light was set at 332 nm and emission recorded in the range 400 – 720 nm using an integration time of 0.5 seconds, increment of 1.0 nm, excitation slit of 0.2 nm and emission slit of 0.5 nm.

Quantum yields were measured using quinine sulfate in 0.05 M H_2SO_4 as a standard (Φ_{em}= 0.59, λ_{ex} = 350 nm).[1] Emission lifetime measurements were performed on the FluoroSENS instrument. Measurements were taken of 1 mL of 0.1 absorbance samples of Eu(III) complexes in 10 mM HEPES at pH 7.0, unless stated otherwise. Measurements were obtained by indirect excitation of the Eu(III) ion via the quinoline antennae using a short pulse of light at 322 nm followed by monitoring the integrated intensity of the light emitted at 615 nm, with 500 data points collected over a 10 millisecond time period. The decay curves were plotted in Origin Labs 2019 version 9.6.0.172, and fitted to the equation:

$$I = A_0 + A_1\, e^{-kt} \qquad (1)$$

where I is the intensity at time, t, following excitation, A_0 is the intensity when decay has ceased, A_1 is the pre-exponential factor and k is the rate constant for the depopulation of the excited state.

The hydration state, *q*, of the Eu(III) complexes was determined using the modified Horrocks equation[2]:

$$q\ (\mathrm{Eu}) = 1.2\ (1/\tau_{H2O} - 1/\tau_{D2O} - 0.25 - 0.075n) \qquad (2)$$

where τ_{H2O} and τ_{D2O} are the emission lifetime times in water and D_2O, respectively, and n is the number of carbonyl-bound amide NH groups.

Plate reader data was obtained on a BMG Labtech CLARIOstar microplate reader using black Fisherbrand™ 384-well plates, using a total volume of 40 µL per well.

Anion binding titrations

Anion binding titrations were carried out in duplicate in degassed 10 mM HEPES buffer at pH 7.0. Stock solutions of anions (e.g. inorganic phosphate, AMP) containing Eu(III) complex (5 µM) were made up at 0.4, 4 and 40 mM anion. The appropriate anion stock solution was added incrementally to 100 µL of Eu(III) complex (5 µM) and the emission spectrum was recorded after each addition. The ratio of emission bands 605 – 630 nm/ 585 – 600 nm ($\Delta J = 2$ / $\Delta J = 1$) was plotted as a function of anion concentration. The data was analysed using a nonlinear least-squares curve fitting procedure, based on a 1:1 binding model described by the equation:

$$\mathrm{FB} = \frac{\frac{1}{\mathrm{K_a}} + [\mathrm{A}] + [\mathrm{Eu}] - \sqrt{(\frac{1}{\mathrm{K_a}} + [\mathrm{A}] + [\mathrm{Eu}])^2 + 4[\mathrm{A}][\mathrm{Eu}]}}{2[\mathrm{Eu}]}$$

where FB is the fraction bound, calculated by $(I - I_0)/(I_1 - I_0)$ where I is the emission intensity at [A], I_0 is the initial emission intensity, and I_1 is the final emission intensity. [A] is the total concentration of anion in solution, [Eu] is the total concentration of Eu(III) complex, K_a is the apparent binding constant.

pH titrations

A solution of Eu(III) complex (5 µM) in water was adjusted to pH 3.5 by the addition of 1 M HCl and an emission spectrum was recorded. The pH was increased slowly by 0.2 – 0.5 units by the addition of 1 M or 0.1 M NaOH solution, and an emission spectrum recorded at each pH. The ratio of emission bands 605 – 630 nm/ 585 – 600 nm ($\Delta J = 2$ / $\Delta J = 1$) was plotted as a function of pH and fitted to a sigmoidal curve using OriginLab 2019 to determine the pK_a value.

Microplate-based enzyme simulations

Different ratios of a solution of enzymatic substrate and product(s) (e.g. cAMP and AMP, respectively) containing a known concentration Eu(III) complex (500 nM unless stated otherwise) in 10 mM HEPES at pH 7.0 were added to a 384-well plate, in triplicate, to a total well volume of 30 µL. The plate was incubated for 10 minutes prior to reading. Time-resolved emission intensities were recorded in the range 605 – 630 nm (integration time of 60 – 400 µs)

with excitation at 292 – 366 nm. The mean of the triplicate intensity values was plotted against the percentage of enzymatic product(s). Error bars indicate the standard error in the mean value.

Phosphodiesterase reaction monitoring

Phosphodiesterase reactions were carried out in duplicate in degassed 10 mM HEPES buffer at pH 7.0. To a solution containing [**Eu.mBOH$_2$**]$^+$ (10 µM) and $MgCl_2$ (5 mM) and if appropriate calmodulin (2 µM) and $CaCl_2$ (60 µM) in a 384-well plate was added 4 µL of a solution containing phosphodiesterase (0.1 mg mL^{-1}). The plate was incubated for 30 minutes. cAMP (200 µM) was added, and the plate read immediately. Luminescence intensities were taken with excitation at 292 – 366 nm and emission using time-resolved measurements at 615 – 625 nm using an integration time of 60 – 400 µs, at 17-minute time intervals. Assays were run in triplicate and the mean of the triplicate intensity values were taken and plotted against time. A background experiment (containing no enzyme) was performed in parallel and subtracted from experiments containing enzyme.

Computational details

Density functional theory (DFT) computations were performed using the wB97M-V functional[3] along with the 6-31G* basis set.[4] A large-core quasi-relativistic effective core potential (ECP), ECP52MWB,[5] was used for treating the core along with the $4f^6$ shell of Eu(III) and the associated (7s6p5d)/[5s4p3d] basis set was used for the valence electrons. All computations were performed using spin-restricted orbitals using a pseudo-singlet configuration. Solvation in water was modelled using a conductor-like polarisable continuum model[6]. All computations were carried out in Q-Chem 5.4.[7] The level of theory was verified against a previously reported crystal structure[8] showing that the bond lengths for coordination around the europium atom are reproduced with a mean absolute error below 0.04 Å. The underlying computational research data (molecular structures and input files including ECP definition) are provided via separate repository.§

§ Supporting research data available: molecular structures and input files including ECP definition. DOI: 10.17028/rd.lboro.16685125.

Synthesis and characterisation of ligands and corresponding Eu(III) complexes

High Performance Liquid Chromatography

Preparative RP-HPLC was performed using a Waters 2489 UV/Visible detector performed at 254 nm, a Waters 1525 Binary HPLC pump controlled by the Waters Breeze 2 HPLC system software. Separation was achieved using a semi-preparative XBridge C18 (5 µm OBD 19 × 100 mm) column at a flow rate maintained at 17 mL min^{-1}. A solvent system composed of either water (0.05% formic acid) / acetonitrile (0.05% formic acid) or water (25 mM NH_4HCO_3) / acetonitrile was used over the stated linear gradient (usually 0 to 100% organic solvent over 10 min). Analytical RP-HPLC was performed using a XBridge C18 (5 µm 4.6 × 100 mm) column at a flow rate maintained at 2.0 mL min^{-1} using the stated gradient and solvents.

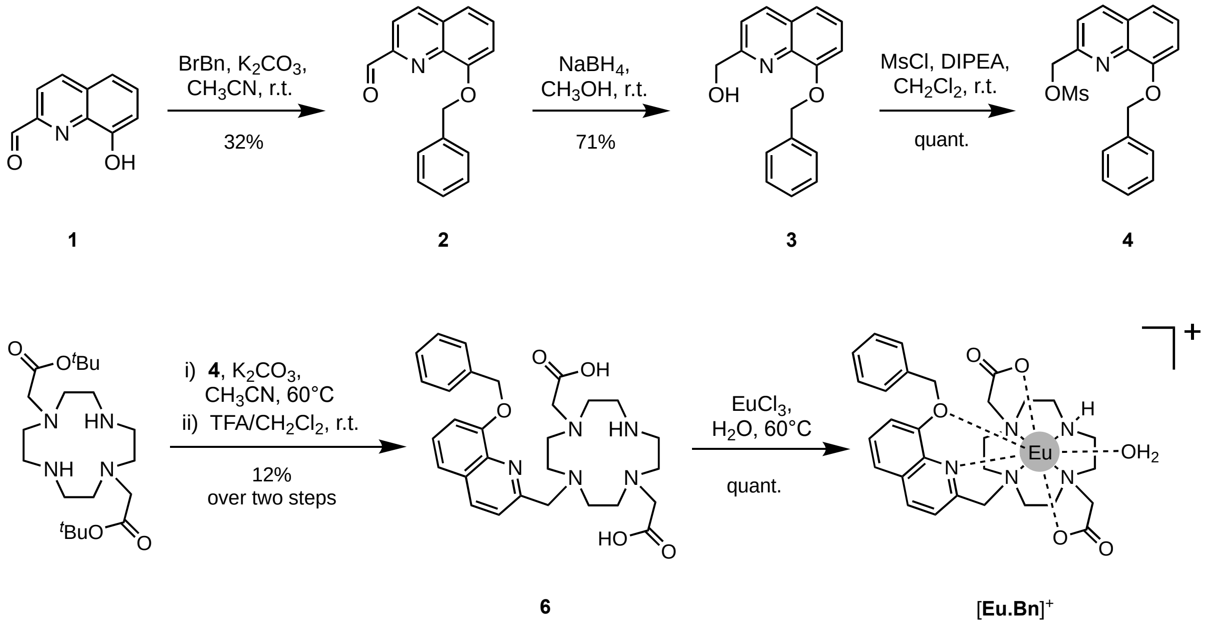

Scheme S1. Synthesis of [**Eu.Bn**]$^{+}$.

8-Hydroxyquinoline-2-carbaldehyde (1)

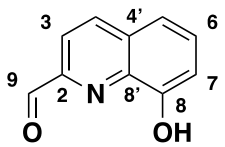

To a stirred solution of commercially available 2-methylquinolin-8-ol (1.00 g, 6.28 mmol) in 1,4-dioxane (15 mL) was added selenium dioxide (0.871 g, 7.85 mmol). The bright yellow

reaction mixture was stirred at 80°C for 24 hours. The mixture was filtered over celite and washed with EtOAc (3 × 10 mL). The organic fractions were combined, and the solvent removed under reduced pressure. A dark orange solid was collected as the crude material, which was purified by column chromatography (silica gel; 1:4 EtOAc/hexane) to give 8-hydroxyquinoline-2-carbaldehyde **1** as a yellow solid (0.657 g, 60%). ^{1}H NMR (400 MHz, $CDCl_3$): δ 10.18 (1H, s, H^{9}), 8.28 (1H, d, *J* = 8.2 Hz, H^{4}), 8.13 (1H, s, O-H), 8.01 (1H, d, *J* = 8.2 Hz, H^{3}), 7.59 (1H, t, *J* = 8.0 Hz, H^{6}), 7.39 (1H, dd, *J* = 8.2 Hz, 0.8 Hz, H^{5}), 7.25 (1H, dd, *J* = 7.6 Hz, 1.0 Hz, H^{7}). ^{13}C NMR (100 MHz, $CDCl_3$): δ 192.8 (C^{9}), 153.1 (C^{8}), 150.2 (C^{2}), 137.9 (C^{4}), 137.5 (C$^{8'}$), 131.0 (C^{6}), 130.5 (C$^{4'}$), 118.1 (C^{5}), 118.0 (C^{3}), 111.3 (C^{7}). R_f = 0.35 (1:5 EtOAc/hexane). HRMS (ESI+) calculated for $[C_{10}H_7NO_2Na]^+$ *m*/*z* 196.0369, found 196.0370. The spectroscopic data were in agreement with those reported previously.[9]

8-((Phenylmethoxy)-2-quinoline)-carbaldehyde (2)

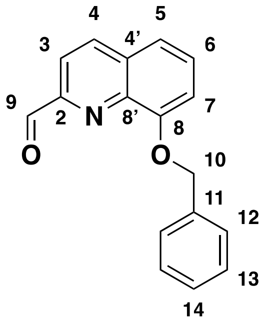

To a stirred solution of 8-hydroxyquinoline-2-carbaldehyde **1** (100 mg, 0.58 mmol) in acetonitrile (5 mL) under a nitrogen atmosphere was added potassium carbonate (120 mg, 0.87 mmol) causing an orange colouration. After 5 minutes, benzyl bromide (137 mL, 1.15 mmol) was added and stirring continued for 20 hours. Solvent was removed *in vacuo* and the residue suspended in water (20 mL) which was extracted with EtOAc (2 × 10 mL). Combined organics were washed with brine, dried over $MgSO_4$ and evaporated to a yellow oil which was purified by column chromatography (silica gel; 1:9 EtOAc/petroleum ether) to give compound **2** (49 mg, 32%) as an off-white solid. ^{1}H NMR (400 MHz, $CDCl_3$): 10.30 (1H, s, H^{9}), 8.24 (1H, d, *J* = 8.5 Hz, H^{4}), 8.04 (1H, d, *J* = 8.5 Hz, H^{3}), 7.56 – 7.48 (3H, m, H^{6}, H^{12}), 7.43 (1H, dd, *J* = 8.2 Hz, 1.1 Hz, H^{5}), 7.41 – 7.35 (2H, m, H^{13}), 7.31 – 7.28 (1H, m, H^{14}), 7.13 (1H, dd, *J* = 7.7 Hz, 1.1 Hz, H^{7}), 5.46 (2H, s, H^{10}). ^{13}C NMR (100 MHz, $CDCl_3$): 194.0 (C^{9}), 155.3 (C^{2}), 151.6 (C^{8}), 140.4 (C$^{8'}$), 137.3 (C^{11}), 136.6 (C^{4}), 131.5 (C$^{4'}$), 129.7 (C^{14}), 128.8 (C^{13}), 128.1 (C^{6}), 127.2 (C^{12}), 120.0 (C^{5}), 117.9 (C^{3}), 111.1 (C^{7}), 71.2 (C^{10}). HRMS (ESI+) calculated for $[C_{17}H_{14}NO_2]^+$ *m*/*z* 264.1018, found 264.1019.

8-((Phenylmethoxy)-2-quinoline)methanol (3)

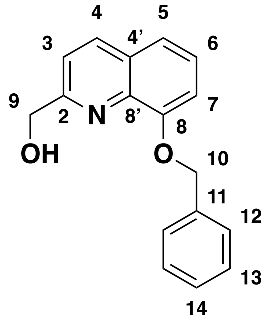

To a stirred solution of aldehyde **2** (160 mg, 0.61 mmol) in methanol (5 mL) at 0°C under a nitrogen atmosphere was added sodium borohydride (35 mg, 0.93 mmol) causing effervescence. After 1 hour the reaction was quenched by the addition of saturated aqueous NH_4Cl (20 mL) and the whole extracted with EtOAc (3 × 15 mL). Combined organics were washed with brine, dried over $MgSO_4$ and evaporated to a yellow semi-solid which was purified by column chromatography (silica gel; 25 – 50% EtOAc/petroleum ether) to give the desired compound **3** (115 mg, 71%) as a pale-yellow solid. ^{1}H NMR (500 MHz, $CDCl_3$): 8.10 (1H, d, *J* = 8.5 Hz, H^4), 7.55 (2H, d, *J* = 7.3 Hz, H^{12}), 7.43 – 7.36 (4H, m, H^5, H^6, H^{13}), 7.36 – 7.29 (2H, m, H^3, H^{14}), 7.13 (1H, m, H^7), 5.37 (2H, s, H^{10}), 4.94 (2H, s, H^9). ^{13}C NMR (100 MHz, $CDCl_3$): 158.2 (C^2), 154.1 (C^8), 139.1 ($C^{8'}$), 137.1 (C^{11}), 136.9 (C^4), 128.9 ($C^{4'}$), 128.7 (C^{13}), 128.0 (C^{14}), 127.2 (C^{12}), 126.5 (C^6), 120.1 (C^5), 119.0 (C^3), 111.0 (C^7), 71.0 (C^{10}), 64.5 (C^9). HRMS (ESI+) calculated for $[C_{17}H_{16}NO_2]^+$ *m/z* 266.1176, found 266.1175.

(8-((Phenylmethoxy)-2-quinoline)-methanesulfonate (4)

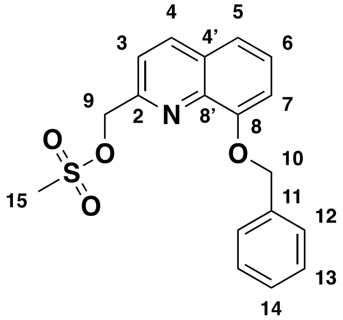

To a stirred solution of alcohol **3** (163 mg, 0.62 mmol) in dichloromethane (10 mL) cooled in an ice-bath was added triethylamine (129 mL, 0.93 mmol) followed by methanesulfonyl chloride (57 mL, 0.74 mmol) as a solution in dichloromethane (2 mL) and stirring continued. After 2 hours the reaction was washed with water (10 mL) and brine (10 mL), dried over $MgSO_4$, evaporated and dried under reduced pressure to give the desired product as a pale

coloured foam which was used without further purification. ^{1}H NMR (500MHz, $CDCl_3$): 8.20 (1H, d, J = 8.5 Hz, H^4), 7.56 (1H, d, J = 8.5 Hz, H^3), 7.52 (2H, d, J = 7.1 Hz, H^{12}), 7.46 – 7.32 (4H, m, H^5, H^6, H^{13}) 7.30 (1H, m, H^{14}), 7.08 (1H, m, H^7), 5.58 (2H, s, H^{10}), 5.37 (2H, s, H^9), 3.14 (3H, s, H^{15}). ^{13}C NMR (100 MHz, $CDCl_3$): 154.5 (C^2), 153.1 (C^8), 139.9 ($C^{8'}$), 137.3 (C^{11}), 136.7 (C^4), 129.1 ($C^{4'}$), 128.7 (C^{13}), 128.2 (C^{14}), 127.7 (C^{12}), 127.3 (C^6), 120.0 (C^5), 119.8 (C^3), 110.6 (C^7), 72.3 (C^{10}), 71.1 (C^9), 38.5 (C^{15}).

Di-*tert*-butyl 2,2'-(4-((8-(phenylmethoxy)-2-quinoline)methyl)-1,4,7,10-tetraazacyclododecane-1,7-diyl)diacetate (5)

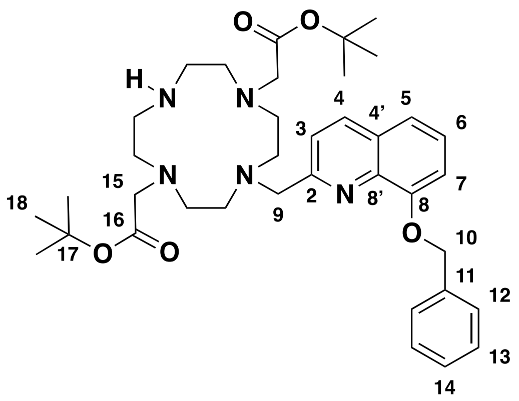

To a stirred solution of mesylate ester **4** (125 mg, 0.36 mmol) in acetonitrile (15 mL) was added potassium carbonate (149 mg, 1.08 mmol) followed by DO2A (di-*tert*-butyl 2,2'-(1,4,7,10-tetraazacyclododecane-1,7-diyl)diacetate) (209 mg, 0.52 mmol) and the mixture was heated at 60°C for 19 hours. The reaction mixture was cooled and centrifuged. The liquours were decanted and the solid washed with dichloromethane (2 × 10 mL). Combined organic fractions were evaporated to give a yellow gum which was purified by column chromatography (silica gel, 25 g) eluting with 95:5 dichloromethane/methanol then with 95:5:0.5 dichloromethane/methanol/NH_3 causing product to elute contaminated with bis-alkylated product. Further elution with the same system yielded fractions containing no material. Elution with 90:10:1 dichloromethane/methanol/NH_3 yielded product free of bis-alkylated material but contaminated with ca. 1.75 equiv. of DO2A. These fractions were combined, evaporated and dried under high vacuum for 2 hours to give the desired product **5** (114 mg, 24% accounting for impurity) as a colourless oil. ^{1}H NMR (400MHz, $CDCl_3$): 8.09 (1H, d, J = 8.4 Hz, H^4), 7.56 (1H, br m, H^3), 7.47 (2H, d, J = 7.4 Hz, H^{12}), 7.40 – 7.32 (4H, m, H^5, H^6, H^{13}), 7.29 (1H, m, H^{14}), 7.07 (1H, m, H^7), 5.32 (2H, s, H^{10}), 3.96 (2H, s, H^9), 3.16 (4H, bs, H^{15}) 3.00 – 2.70 (16H, m, H^{cyclen}, also some DO2A signals), 1.37 (18H, s, H^{18}), ^{13}C NMR (100 MHz, $CDCl_3$): 170.8

(C^{16}), 157.8 (C^{2}),154.2 (C^{8}), 140.0 ($C^{8'}$), 136.9 (C^{11}), 136.6 (C^{4}), 128.7 ($C^{4'}$), 128.5 (C^{13}), 128.1 (C^{14}), 127.5 (C^{12}), 126.5 (C^{6}), 122.4 (C^{3}), 120.1 (C^{5}), 110.4 (C^{7}), 81.1 (C^{17}), 71.0 (C^{10}), 57.5 (C^{9}), 54.2 (C^{cyclen}) 52.0 (C^{15}) 51.4 (C^{cyclen}), 50.2 (C^{cyclen}), 47.1 (C^{cyclen}), 28.3 (C^{18}). HRMS (ESI+) calculated for $[C_{37}H_{54}N_5O_5]^+$ *m/z* 648.4119, found 648.4120.

2,2'-(4-((8-(phenylmethoxy)-2-quinoline)-methyl)-1,4,7,10-tetraazacyclododecane-1,7-diyl)diacetic acid (6)

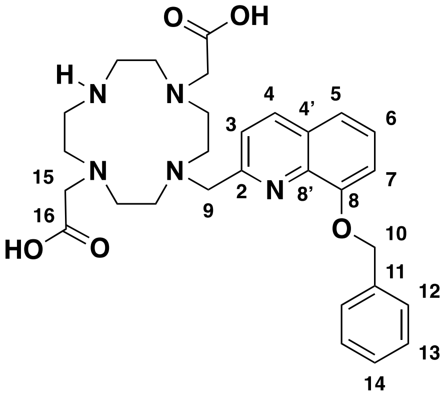

To a stirred solution of protected ligand **5** (57 mg, 0.04 mmol accounting for DO2A impurity) in dichloromethane (7.5 mL) was added trifluoroacetic acid (2.5 mL) and the reaction mixture stirred overnight. Solvent was removed under reduced pressure and the residue co-evaporated from dichloromethane (5 × 10 mL) to give the crude product as a brown gum. This was purified by preparative HPLC (gradient: 0 − 100% acetonitrile in 25 mM aqueous NH_4CO_3 over 15 min). Acetonitrile was removed under reduced pressure and aqueous by freeze-drying to give the pure ligand (12 mg, 0.02 mmol, 50%). ^{1}H NMR (400MHz, methanol- d_4): 8.31 (1H, m, H^{4}), 7.70 – 7.34 (9H, m, H^{3}, $H^{5\text{-}7}$, $H^{12\text{-}14}$), 5.37 (2H, s, H^{10}) 4.12 (2H, s, H^{9}), 3.10 – 2.70 (20H, br m, H^{17}, H^{cyclen}), ^{13}C NMR (100 MHz, methanol-d_4): 176.2 (C^{16}), 155.5 (C^{2}), 153.3 (C^{8}), 139.0 ($C^{8'}$), 136.7 (C^{4}), 135.9 (C^{11}), 128.2 ($C^{4'}$), 128.1 (C^{12}), 128.0 (C^{13}), 127.9 (C^{6}), 126.2 (C^{14}), 122.7 (C^{3}), 119.6 (C^{5}), 109.6 (C^{7}), 70.4 (C^{10}), 58.4 (C^{9}), 56.4 (C^{15}), 54.0 (C^{cyclen}), 50.0 (C^{cyclen}), 49.2 (C^{cyclen}), 43.8 C^{cyclen}). HRMS (ESI+) calculated for $[C_{29}H_{38}N_5O_5]^+$ *m/z* 536.2867, found 536.2868.

[**Eu.Bn**]$^{+}$

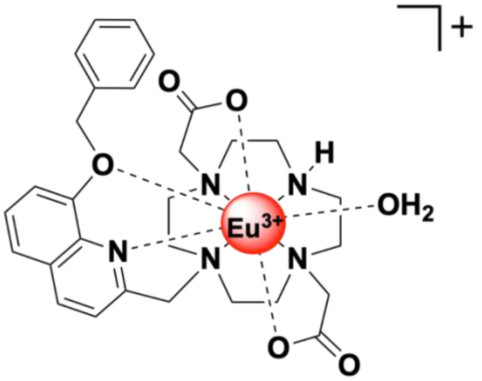

Pure ligand **6** (12 mg, 0.022 mmol) was dissolved in water (2 mL) and the pH adjusted to 8.9. $EuCl_3.6H_2O$ (8.2 mg, 0.022 mmol) was added and the mixture stirred for 30 minutes, after which time the reaction was again basified to pH 8.5. The reaction was stirred at 60°C for 4 hours. The water was removed by freeze-drying to give the Eu(III) complex, [**Eu.Bn**]$^{+}$ (15 mg, 100%) as a white solid. ^{1}H NMR (400MHz, D_2O): 27.23, 13.53, 12.88, 11.81, 9.16, 9.00, 8.91, 8.74, 7.70, 7.58, 7.48, 7.18, 5.59, 5.53, 3.41, 3.23, 2.83, 1.40, 1.33, -1.45, -13.08. HRMS (ESI+) calculated for $[C_{29}H_{35}EuN_5O_5]^+$ *m/z* 686.1845, found 686.1843. Photophysical data measured in 10 mM HEPES at pH 7.0: λ_{max} = 322 nm, ε = 2900 M^{-1} cm^{-1}, Φ_{em} = 1.7%, τ_{H2O} = 0.192 ms, τ_{D2O} = 0.252 ms, q = 1.2.

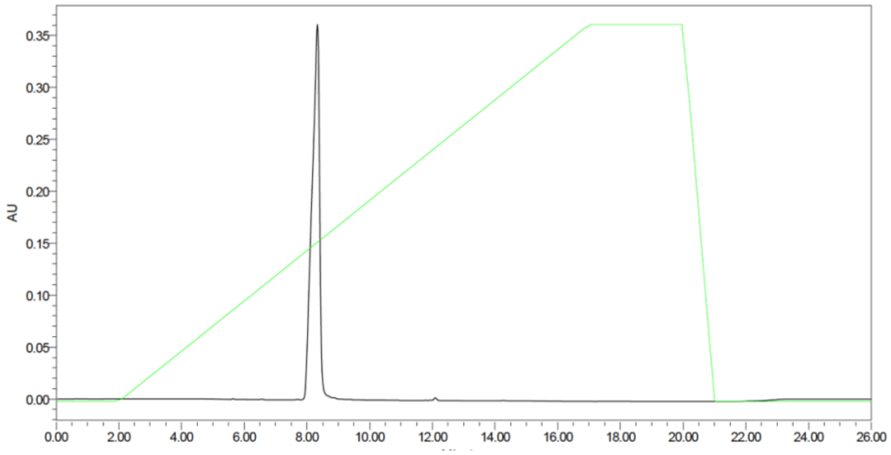

Analytical RP-HPLC trace of [**Eu.Bn**]$^{+}$ [gradient: 0 − 100% acetonitrile in 25 mM aqueous NH_4CO_3 over 15 min; at 0.7 mL per minute].

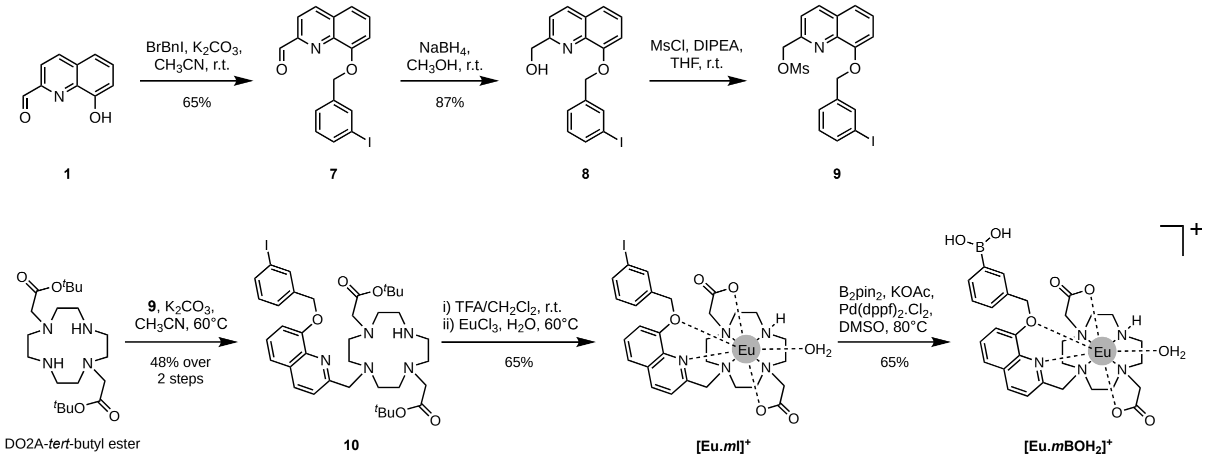

Scheme S2. Synthesis of [**Eu.*m*BOH2**]$^+$

8-((3-Iodobenzyl)oxy)quinoline-2-carbaldehyde (7)

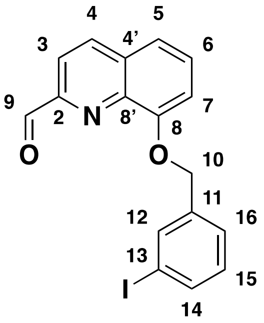

To a solution of aldehyde **1** (0.500 g, 2.89 mmol) in acetonitrile (25 mL) was added potassium carbonate (1.20 g, 8.65 mmol) and 3-iodobenzyl bromide (1.20 g, 4.16 mmol) and the resulting yellow solution was stirred at room temperature for 24 hours. The resulting orange solution was centrifugated at 120 rpm for 3 minutes. The solution was decanted, and the solid pellet was washed twice with dichloromethane (3 × 15 mL) and the solvent was evaporated under reduced pressure. The resulting residue was partitioned between dichloromethane (10 mL) and saturated aqueous sodium chloride (10 mL). The aqueous phase was extracted with dichloromethane (3 × 10 mL) and the combined organic phases were dried over $MgSO_4$, filtered and concentrated under reduced pressure. The crude material was purified by column chromatography (silica gel; 20 – 80% EtOAc/petroleum ether) to give the desired aldehyde **7** (0.770 g, 65%) as a white solid. ^{1}H NMR (400 MHz, $CDCl_3$) δ 10.30 (1H, s, H^9), 8.26 (1H, d, J = 8.2 Hz, H^4), 8.05 (1H, d, J = 8.7 Hz, H^3), 7.93 (1H, s, H^{12}), 7.65 (1H, d, J = 7.8 Hz, H^{14}), 7.55 – 7.45 (3H, m, H^6, H^{16}, H^5), 7.13 – 7.09 (2H, m, H^7, H^{15}), 5.39 (2H, s, H^{10}). ^{13}C NMR (100 MHz, $CDCl_3$) δ 193.9 (C^9), 155.0 (C^8), 151.7 (C^2), 140.3 ($C^{8'}$), 139.0 (C^{11}), 137.4 (C^4), 137.2 (C^{14}), 136.0 (C^{12}), 131.5 ($C^{4'}$), 130.5 (C^{15}), 129.7 (C^6), 126.3 (C^{16}), 120.4 (C^5), 118.1 (C^3),

111.2 (C[7]), 94.7 (C[13]), 70.3 (C[10]). HRMS (ESI+) calculated for $[C_{17}H_{13}O_2NI]^+$ *m/z* 389.9985, found 389.9984.

(8-((3-Iodobenzyl)oxy)quinolin-2-yl)methanol (8)

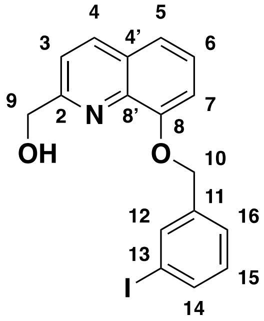

To a solution of aldehyde **7** (0.450 g, 1.16 mmol) in methanol (10 mL) was added sodium borohydride (0.160 g, 4.2 mmol) and the reaction mixture was stirred at 30°C for 24 hours. The reaction was quenched with deionised water (3 mL) and the solvent was removed under reduced pressure. The crude material was partitioned between dichloromethane (20 mL) and saturated sodium chloride solution (10 mL). The aqueous layer was extracted with dichloromethane (3 × 15 mL) and the organic layers were combined, dried over $MgSO_4$ and concentrated under reduced pressure to give the desired alcohol **8** (0.400 g, 88%) as a white solid. ^{1}H NMR (400 MHz, $CDCl_3$) δ 8.11 (1H, d, *J* = 8.7 Hz, H[4]), 7.91 (1H, s, H[12]), 7.65 (1H, d, *J* = 7.8 Hz, H[14]), 7.51 (1H, d, *J* = 7.8 Hz, H[16]), 7.43 – 7.38 (2H, m, H[5], H[6]), 7.35 (1H, d, *J* = 8.7 Hz, H[3]), 7.14 – 7.08 (2H, m, H[15], H[7]), 5.29 (2H, s, H[10]), 4.95 (2H, s, H[9]), 4.53 (1H, br s, O-H). ^{13}C NMR (100 MHz, $CDCl_3$) δ 158.3 (C[2]), 153.9 (C[8]), 139.5 (C[8']), 139.1 (C[11]), 137.0 (C[14]), 136.9 (C[4]), 136.0 (C[12]), 130.4 (C[15]), 129.0 (C[4']), 126.4 (C[6]), 126.3 (C[16]), 120.5 (C[5]), 119.1 (C[3]), 111.3 (C[7]), 94.5 (C[13]), 70.1 (C[10]), 64.5 (C[9]). HRMS (ESI+) calculated for $[C_{17}H_{15}O_2NI]^+$ *m/z* 392.0142, found 392.0141.

(8-((3-Iodobenzyl)oxy)quinolin-2-yl)methyl methanesulfonate (9)

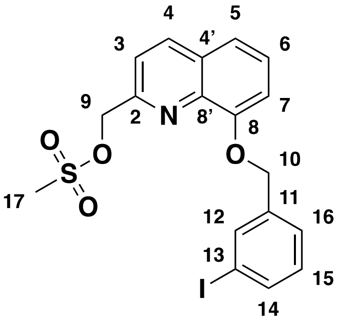

To a solution of alcohol **8** (100 mg, 0.256 mmol) in anhydrous THF (10 mL) was added triethylamine (54 µL, 0.384 mmol) and methanesulfonyl chloride (2.0 µL, 0.28 mmol) to give a yellow solution which was stirred at 30 °C for 2 hours under a nitrogen atmosphere. The solvent was removed under reduced pressure and the residue was partitioned between dichloromethane (20 mL) and saturated sodium chloride solution (10 mL). The aqueous layer was extracted with dichloromethane (3 × 15 mL) and the organic layers combined, dried over $MgSO_4$, filtered and evaporated under reduced pressure to give the desired mesylate ester **9** (85 mg, 71%) as a yellow oil, which was used immediately in the next step. ^{1}H NMR (400 MHz, $CDCl_3$) δ 8.20 (1H, d, *J* = 8.2 Hz, H^4), 7.89 (1H, s, H^{12}), 7.67 (1H, d, *J* = 7.8 Hz, H^{14}), 7.58 (1H, d, *J* = 8.2 Hz, H^3), 7.48 (1H, d, *J* = 7.4 Hz, H^{16}), 7.44 – 7.42 (2H, m, H^6, H^5), 7.14 – 7.07 (2H, m, H^{15}, H^7), 5.58 (2H, s, H^9), 5.30 (2H, s, H^{10}), 3.16 (3H, s, H^{17}). ^{13}C NMR (100 MHz, $CDCl_3$) δ 154.2 (C^8), 153.2 (C^2), 139.9 ($C^{8'}$), 139.2 (C^{11}), 137.4 (C^4), 137.2 (C^{14}), 136.3 (C^{12}), 130.5 (C^{15}), 129.1 ($C^{4'}$), 127.3 (C^6), 126.7 (C^{16}), 120.4 (C^5), 120.0 (C^3), 110.9 (C^7), 94.6 (C^{13}), 72.3 (C^9), 70.2 (C^{10}), 38.5 (C^{17}). LRMS (ESI+) calculated for $[C_{18}H_{17}INO_4S]^+$ *m/z* 469.9, found 469.8.

2,2'-(4-((8-((3-Iodobenzyl)oxy)quinolin-2-yl)methyl)-1,4,7,10-tetraazacyclododecane-1,7-diyl)diacetic acid (10)

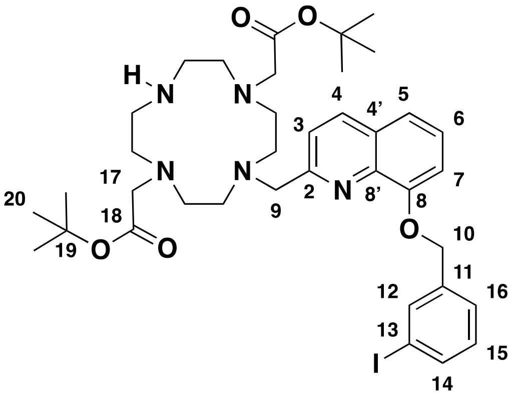

To a solution of DO2A (114 mg, 0.285 mmol) and potassium carbonate (79 mg, 0.570 mmol) in anhydrous acetonitrile (20 mL), was added the mesylate ester **9** (160 mg, 0.342 mmol). The solution was stirred at 60°C for 24 hours and then the solution was centrifuged at 1500 rpm for 3 minutes. The organic layer was removed, and the pellet was washed with dichloromethane (2 × 10 mL). The organic layers were combined, and the solvent was removed under reduced pressure. The crude material was purified by column chromatography (silica gel; neat dichloromethane to 92:8 dichloromethane/methanol) to give the desired protected ligand **10**

(117 mg, 53%) as a yellow solid. ^{1}H NMR (400 MHz, $CDCl_3$) δ 9.65 (1H, br s, N-H), 8.14 (1H, d, J = 8.2 Hz, H^4), 7.90 (1H, s, H^{12}), 7.65 (1H, d, J = 7.8 Hz, H^{14}), 7.49 – 7.46 (2H, m, H^3, H^{16}), 7.42 – 7.41 (2H, m, H^6, H^5), 7.14 (1H, t, J = 7.8 Hz, H^{15}), 7.09 (1H, t, J = 4.3 Hz, H^7), 5.29 (2H, s, H^{10}), 4.04 (2H, s, H^9), 3.24 – 2.84 (20H, m, H^{17}, H^{cyclen}), 1.41 (18H, s, H^{20}). ^{13}C NMR (100 MHz, $CDCl_3$) δ 170.7 (C^{18}), 157.2 (C^2), 154.0 (C^8), 140.0 ($C^{8'}$), 139.4 (C^{11}), 137.1 (C^{14}), 136.8 (C^4), 136.2 (C^{12}), 130.5 (C^{15}), 128.5 ($C^{4'}$), 126.6 (C^6), 126.5 (C^{16}), 122.5 (C^3), 120.4 (C^5), 110.7 (C^7), 94.5 (C^{13}), 81.5 (C^{19}), 70.0 (C^{10}), 57.5 (C^{17}), 56.5 (C^9), 54.1 (C^{cyclen}), 51.5 (C^{cyclen}), 49.8 (C^{cyclen}), 46.9 (C^{cyclen}), 29.8 (C^{20}). R_f = 0.25 (5:95 methanol/dichloromethane). HRMS (ESI+) calculated for $[C_{37}H_{53}IN_5O_5]^+$ m/z 774.3091, found 774.3083.

2,2'-(4-((8-((3-Iodobenzyl)oxy)quinolin-2-yl)methyl)-1,4,7,10-tetraazacyclododecane-1,7-diyl)diacetic acid (11)

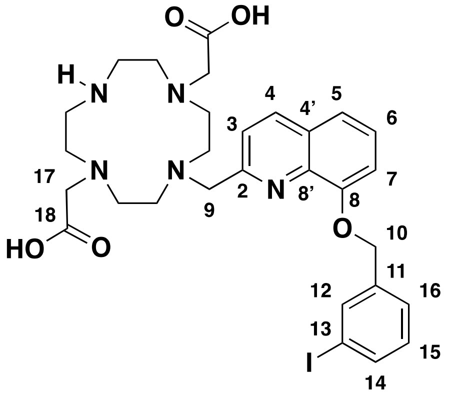

To a solution of di-*tert*-butyl 2,2'-(4-((8-((3-iodobenzyl)oxy)quinolin-2-yl)methyl)-1,4,7,10-tetraazacyclododecane-1,7-diyl)diacetate **10** (80 mg, 0.10 mmol) in dichloromethane (3 mL) was added trifluoroacetic acid (3 mL) and the reaction mixture was stirred at room temperature for 2 hours. The resulting orange solution was evaporated to dryness to give the deprotected ligand **11** (69 mg, 100%) as an orange oil. ^{1}H NMR (400 MHz, methanol- d_4) δ 8.36 (1H, d, J = 8.7 Hz, H^4), 8.01 (1H, s, H^{12}), 7.75 (1H, d, J = 7.8 Hz, H^{14}), 7.63 (1H, d, J = 8.2 Hz, H^3), 7.59 – 7.53 (3H, m, H^{16}, H^5, H^6), 7.31 – 7.22 (2H, m, H^7, H^{15}), 5.31 (2H, s, H^{10}), 4.81 (2H, s, H^9), 4.28 (2H, br s, O-H), 3.71 – 2.93 (20H, m, H^{cyclen}, H^{17}). ^{13}C NMR (100 MHz, CD_3OD) δ 175.0 (C^{18}), 155.4 (C^8), 151.6 (C^2), 141.2 (C^{11}), 140.7 ($C^{8'}$), 139.1 (C^4), 138.3 (C^{14}), 137.8 (C^{12}), 131.7 (C^{15}), 130.5 ($C^{4'}$), 129.1 (C^6), 128.3 (C^{16}), 123.7 (C^3), 121.1 (C^5), 111.5 (C^7), 95.1 (C^{13}), 70.7 (C^{10}), 58.6 (C^9), 54.1 (C^{cyclen}), 53.4 (C^{cyclen}), 50.8 (C^{cyclen}), 50.2 (C^{cyclen}), 43.2 (C^{17}). HRMS (ESI+) calculated for $[C_{29}H_{37}IN_5O_5]^+$ m/z 662.1865, found 662.1843.

[Eu.*m*I]$^+$

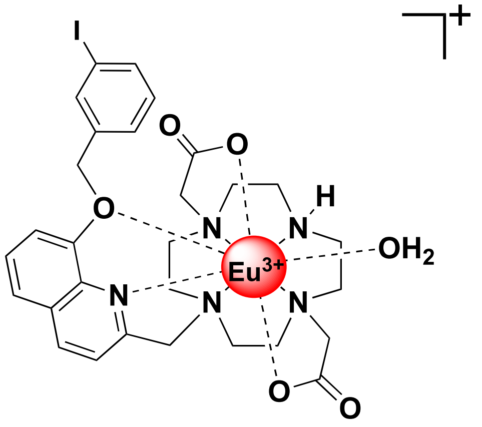

The deprotected ligand **11** (42 mg, 60 µmol) was dissolved in deionised water (10 mL) and the pH adjusted to 7.5 using 0.3 M NaOH solution. $EuCl_3.6H_2O$ (22 mg, 60 µmol) was added and the pH was readjusted to 7.5, before stirring the solution at 70 °C for 24 hours. The solvent was removed by freeze drying to give the Eu(III) complex **[Eu.*m*I]**$^+$ (45 mg, 88%) as a yellow solid, which was used in the next step without further purification. HRMS (ESI+) calculated for $[C_{29}H_{34}EuIN_5O_5]^+$ *m/z* 812.0811, found 812.0812.

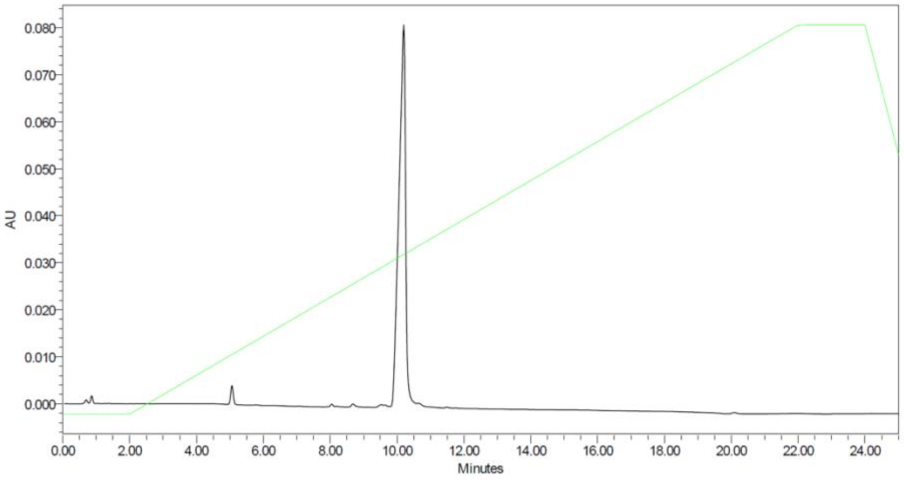

Analytical RP-HPLC trace of [**Eu.*m*I**]$^+$ [gradient: 0 – 100% acetonitrile in 25 mM aqueous NH_4CO_3 over 25 min; at 0.7 mL per minute].

[Eu.*m*BOH2]+

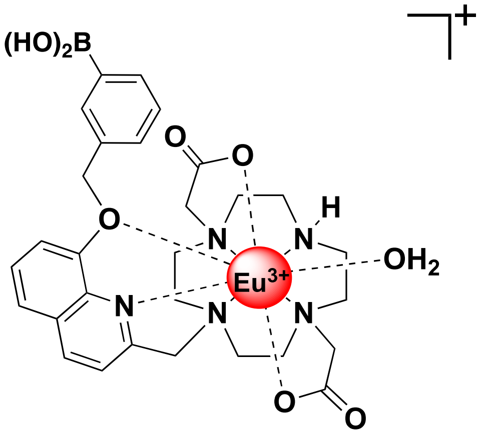

The Eu(III) complex, **[Eu.*m*I]+** (10 mg, 12 μmol) was dissolved in DMSO (2 mL) and potassium acetate (16 mg, 240 μmol) and bis(pinacolato)diboron (13 mg, 51 μmol) were added. The mixture was degassed by freeze-pump-thaw cycle prior to the addition of [1,1′-bis(diphenylphosphino)ferrocene]dichloropalladium(II) (2 mg, 2.7 μmol). The reaction mixture was stirred at 80 °C for 2 hours under an atmosphere of argon. The solution was diluted with 100 mL of deionised water and the solvent was removed by freeze drying. The resulting yellow solid was purified by preparative RP-HPLC [gradient: 0 − 100% acetonitrile in 0.05% *v/v* formic acid, over 10 minutes at 17 mL per min^{-1}; t_R = 6.57 min] to give **[Eu.*m*BOH2]+** (1.3 mg, 44%) as a white solid. ^1H NMR δ (500 MHz, D_2O) δ 27.65, 13.72, 13.31, 11.64, 10.74, 9.44, 9.14, 8.96, 8.81, 8.45, 7.87, 7.66, 7.37, 7.36, 7.21, 6.98, 6.97, 5.90, 5.53, 4.71, 4.62, 3.73, 3.41, 3.21, 2.78, 2.32, 2.29, 1.65, 1.58, 1.56, 1.53, 1.38, 1.28, 0.93, -1.63, -13.62; several signals obscured or overlapping. HRMS (ESI+) calculated for $[C_{29}H_{36}BEuN_5O_7]^+$ *m/z* 730.1915, found 730.1909. Photophysical data measured in 10 mM HEPES at pH 7.0: λ_{max} = 322 nm, ε = 2700 M^{-1} cm^{-1}, Φ_{em} = 1.5%, τ_{H2O} = 0.199 ms, τ_{D2O} = 0.283 ms, q = 1.5.

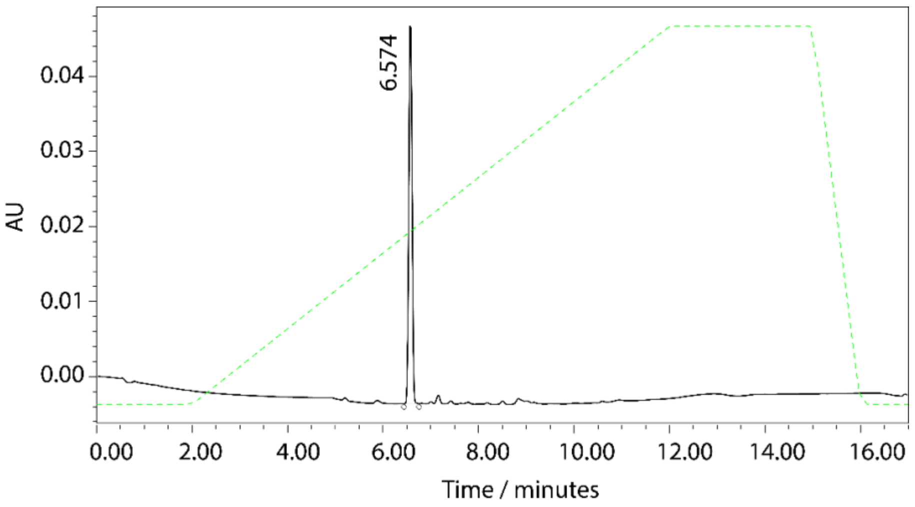

Analytical RP-HPLC trace of [**Eu.*m*BOH$_2$**]$^+$ [gradient: 0 − 100% acetonitrile in 0.05% *v/v* formic acid, over 17 minutes at 0.7 mL per minute].

Photophysical measurements of Eu(III) complexes

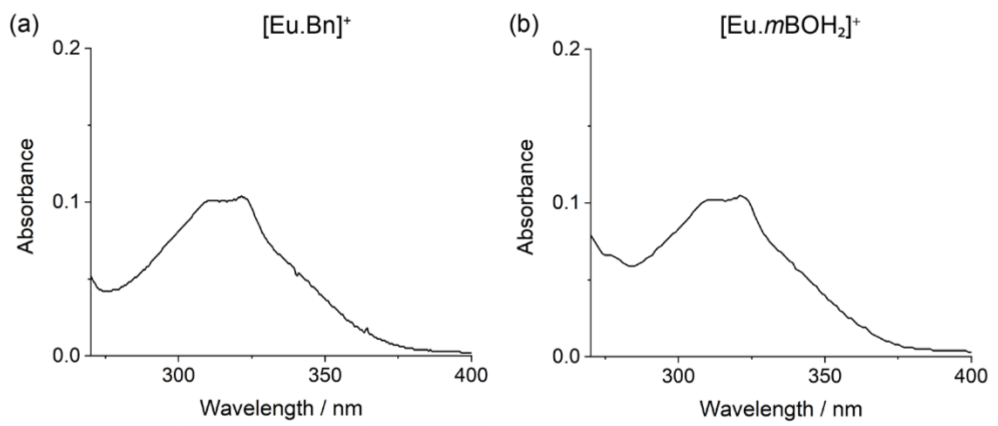

Figure S1. Absorption spectra of (a) **[Eu.Bn]**$^+$ and (b) **[Eu.*m*BOH$_2$]**$^+$, measured in 10 mM HEPES at pH 7.0.

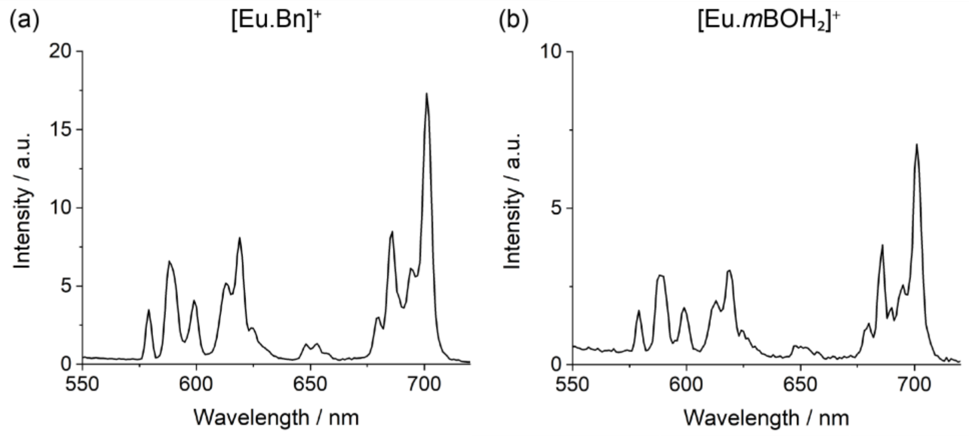

Figure S2. Emission spectra for (a) **[Eu.Bn]**$^+$ and (b) **[Eu.*m*BOH$_2$]**$^+$ measured in 10 mM HEPES at pH 7.0.

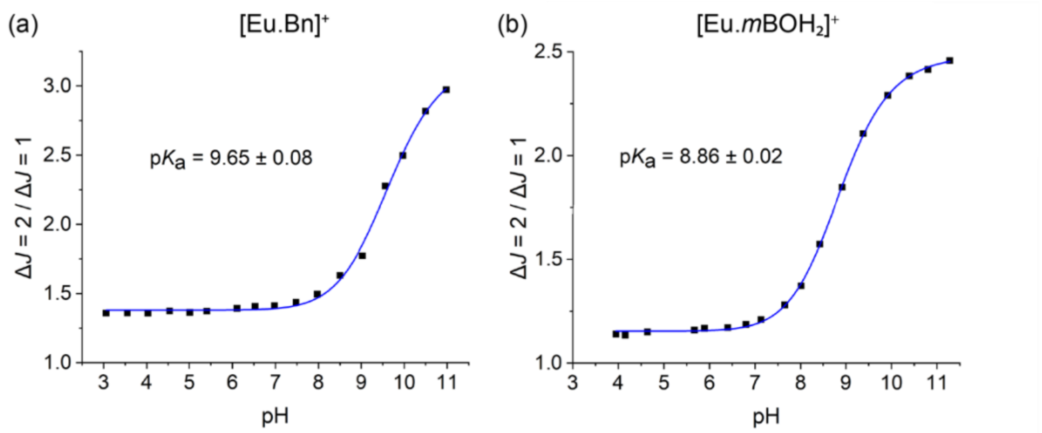

Figure S3. Plot of emission intensity ratio $\Delta J = 2/ \Delta J = 1$ as a function of pH, showing the fit to the observed data (measured in water, 295 K) for (a) **[Eu.Bn]**$^+$ and (b) **[Eu.*m*BOH$_2$]**$^+$.

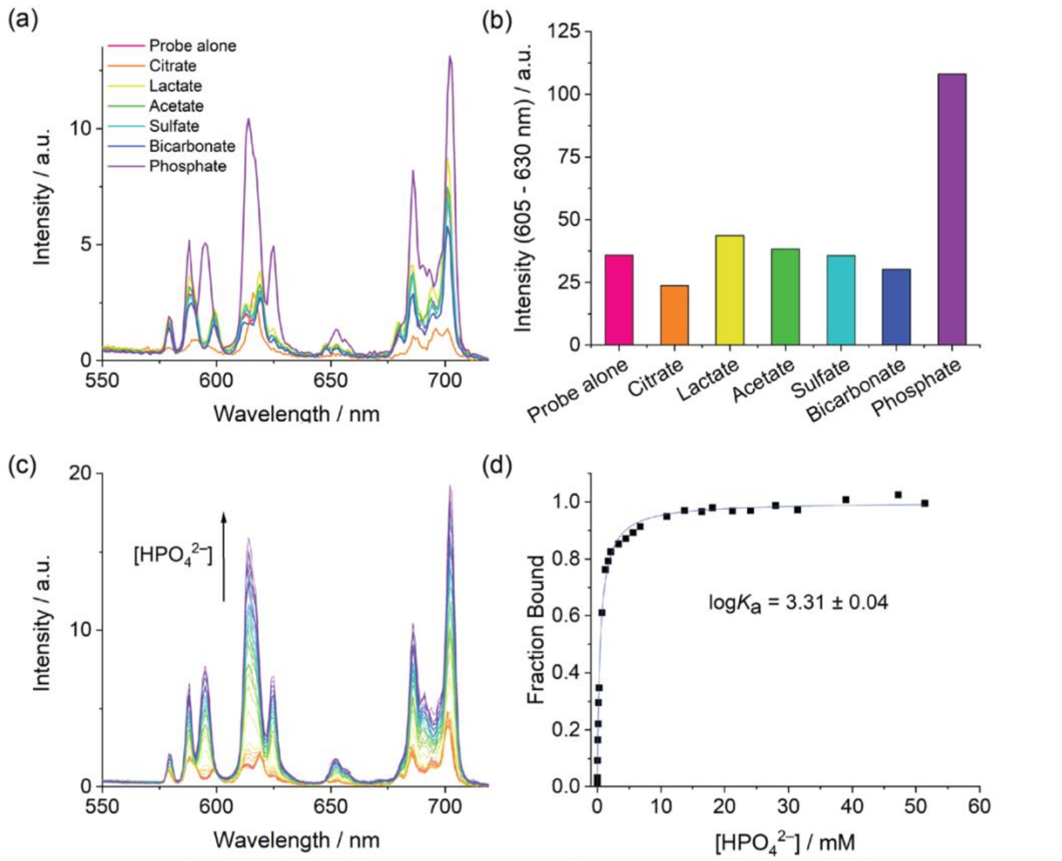

Figure S4. (a and b) Selective emission enhancement of **[Eu.*m*BOH2]**$^{+}$ (5 µM) with phosphate (1 mM) compared with citrate, lactate, acetate, sulfate and bicarbonate (1 mM each); (c) Variation in emission spectra of **[Eu.*m*BOH2]**$^{+}$ upon incremental addition of phosphate; (d) Plot of fraction bound (determined from $\Delta J = 2$ / $\Delta J = 1$ intensity ratio) versus phosphate concentration, showing the fit to a 1:1 binding isotherm. Measured in 10 mM HEPES at pH 7.0 and 295 K, λ_{exc} = 322 nm.

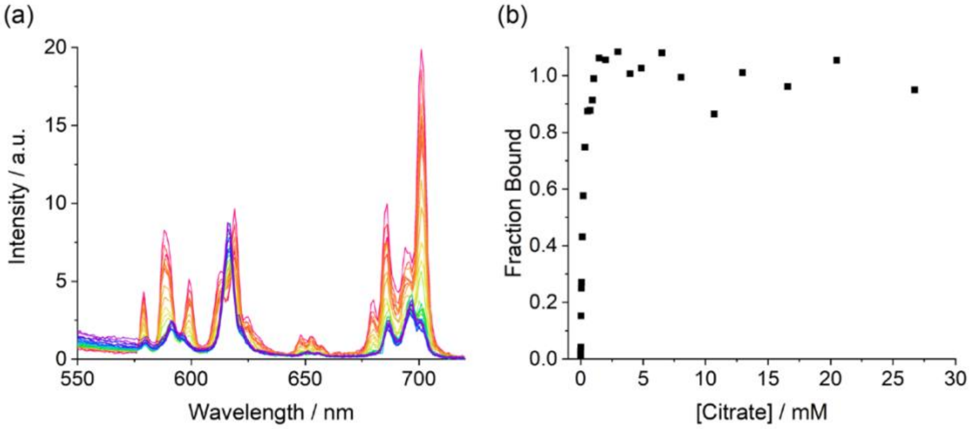

Figure S5. (a) Change in emission spectra of [**Eu.Bn**]$^+$ upon incremental addition of citrate (0–27 mM); (b) Increase in the ratio of emission bands ΔJ = 2 / ΔJ = 1 (605 – 630 nm/ 580 – 600 nm) of [**Eu.Bn**]$^+$. The data could not be fit with confidence to a 1:1 binding isotherm. Measured in 10 mM HEPES at pH 7.0 and 295 K, λ_{exc} = 322 nm.

Table S1. Lifetime values for Eu(III) complexes alone and in the presence of selected anions (1 mM each), measured in 10 mM HEPES at pH 7.0.

Complex	Anion	τ_{H2O} / ms	τ_{D2O} / ms	q [a]
**[Eu.Bn]$^+$**	none	0.192	0.252	1.2
	HPO_4^{2-}	0.456	0.524	0.0
	AMP	0.483	0.545	0.0
	ADP	0.479	0.569	0.1
	ATP	0.347	0.456	0.9
	citrate	0.375	0.524	0.3
**[Eu.*m*BOH$_2$]$^+$**	none	0.199	0.283	1.5
	HPO_4^{2-}	0.436	0.483	0
	AMP	0.485	0.525	-0.1
	ADP	0.495	0.629	0.2
	ATP	0.385	0.507	0.5
	citrate	0.294	0.392	0.7

Values of hydration state q (±20%) were derived using literature methods.[2]

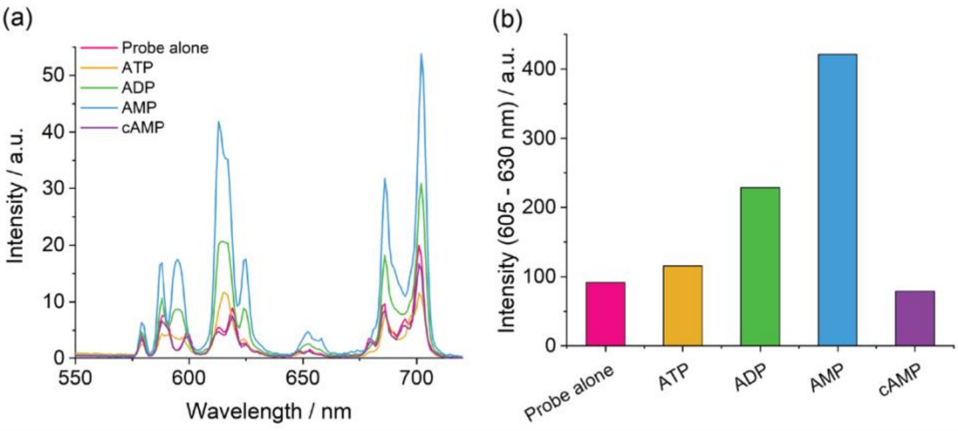

Figure S6. (a and b) Large enhancement in emission intensity of [**EuBn**]$^+$ (5 µM) with AMP compared with ATP, ADP and cAMP (1 mM each). Measured in 10 mM HEPES at pH 7.0 and 295 K, λ_{exc} = 322 nm.

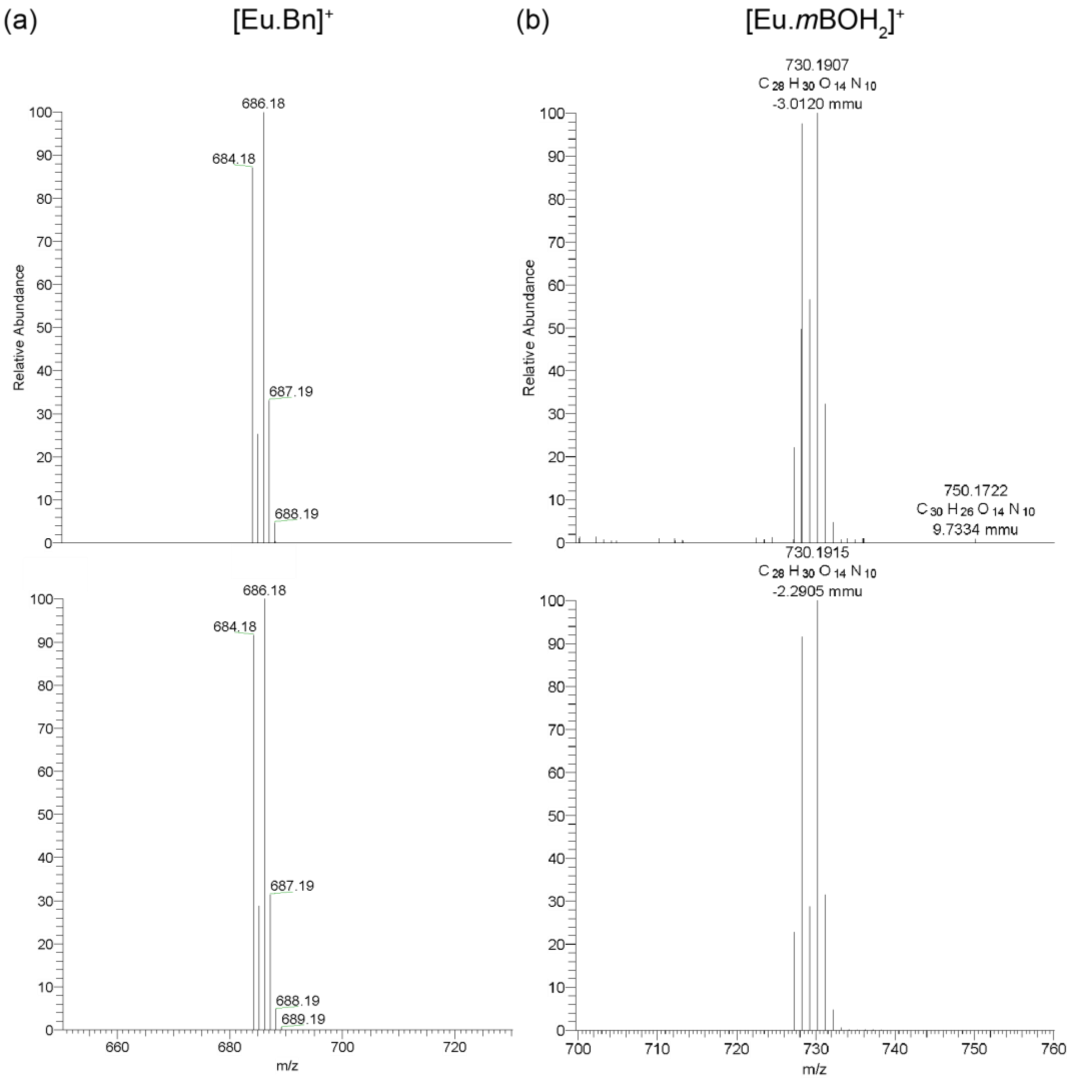

Figure S7. High resolution mass spectra of (a) [**Eu.Bn**]$^+$ and (b) [**Eu.*m*BOH$_2$**]$^+$.

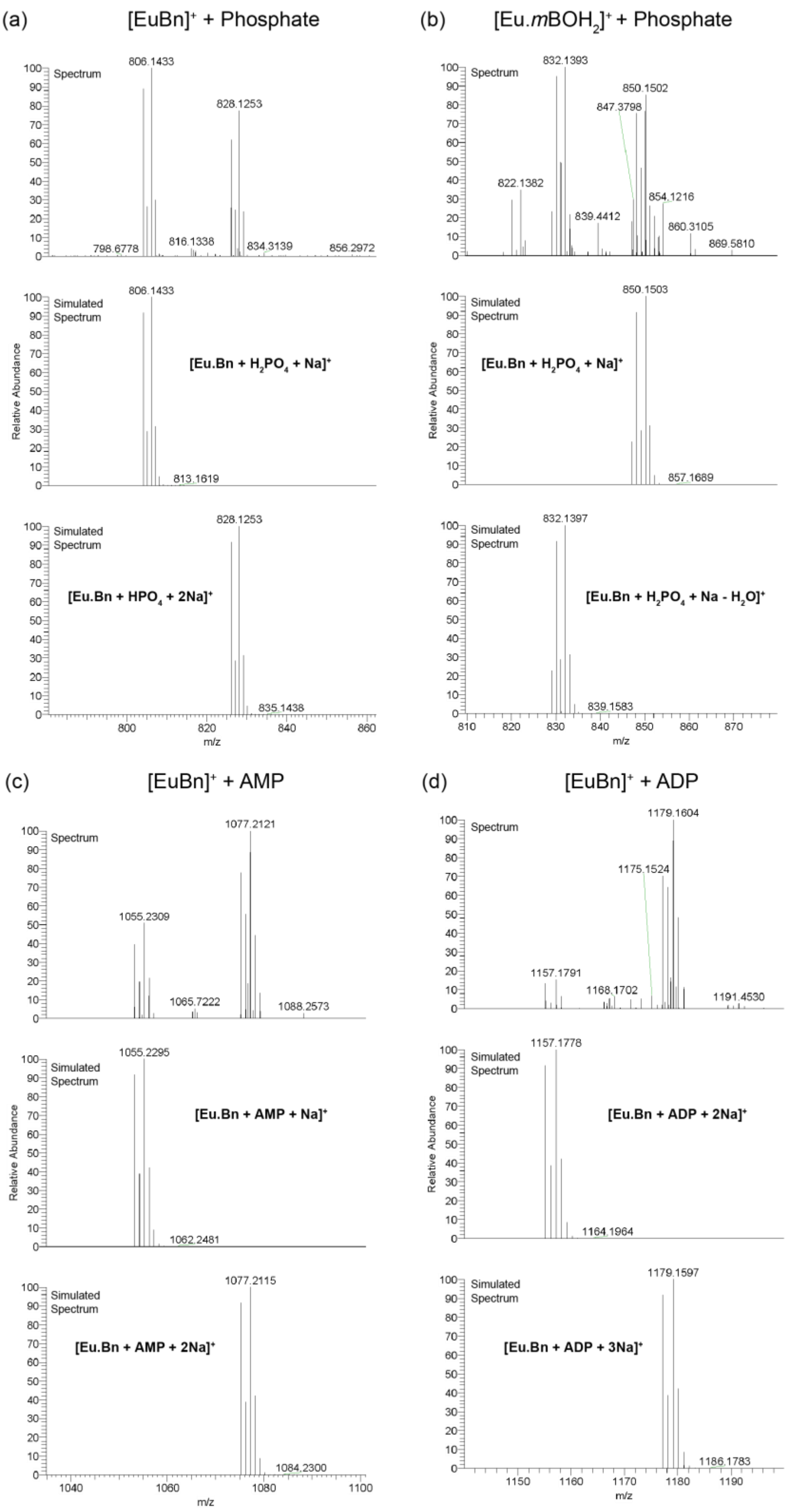

Figure S8. High resolution mass spectra of 1:1 mixtures (1 mM) of (a) [**Eu.Bn**]$^+$ and phosphate, (b) [**Eu.*m*BOH$_2$**]$^+$ and phosphate (also showing the dehydration product common for boronic acids), (c) [**Eu.Bn**]$^+$ and AMP, and (d) [**Eu.Bn**]$^+$ and AMP, showing the formation of 1:1 adducts in all cases.

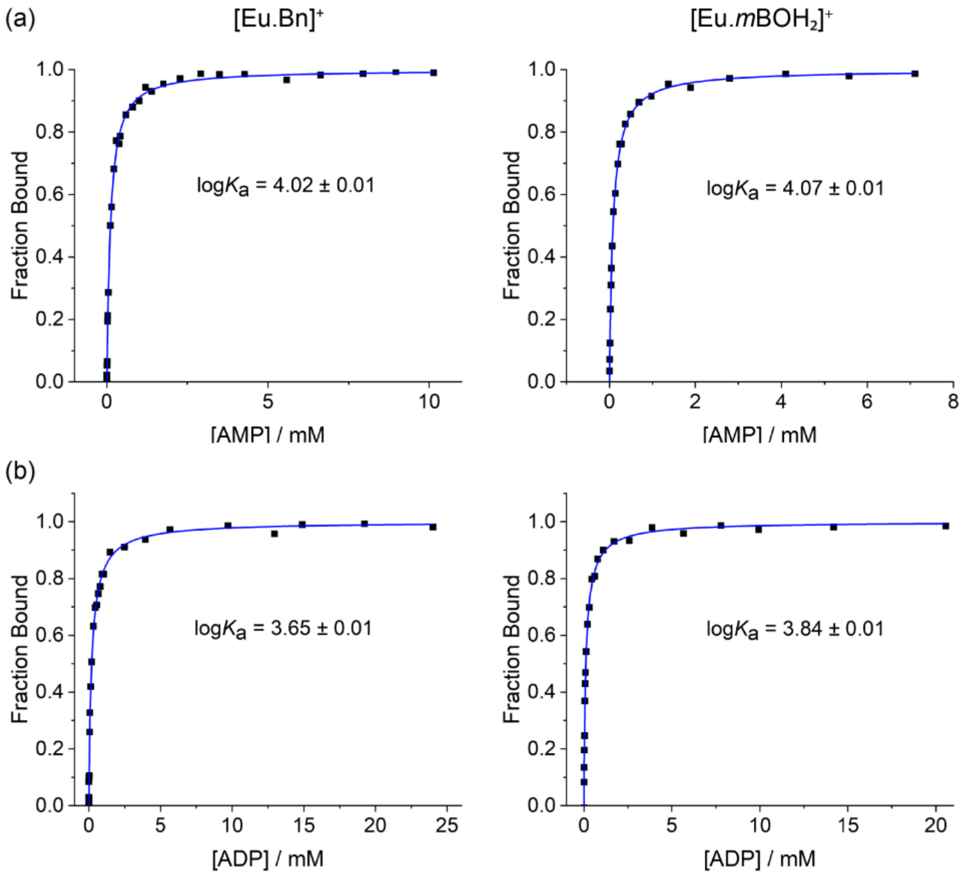

Figure S9. Anion binding isotherms for complexes [**EuBn**]$^+$ and [**Eu.*m*BOH$_2$**]$^+$ with (a) AMP and (b) ADP. Plot of fraction bound (determined from $\Delta J = 2$ / $\Delta J = 1$ intensity ratio) versus anion concentration, showing the fit to a 1:1 binding isotherm. Measured in 10 mM HEPES at pH 7.0. λ_{exc} = 322 nm, 295 K.

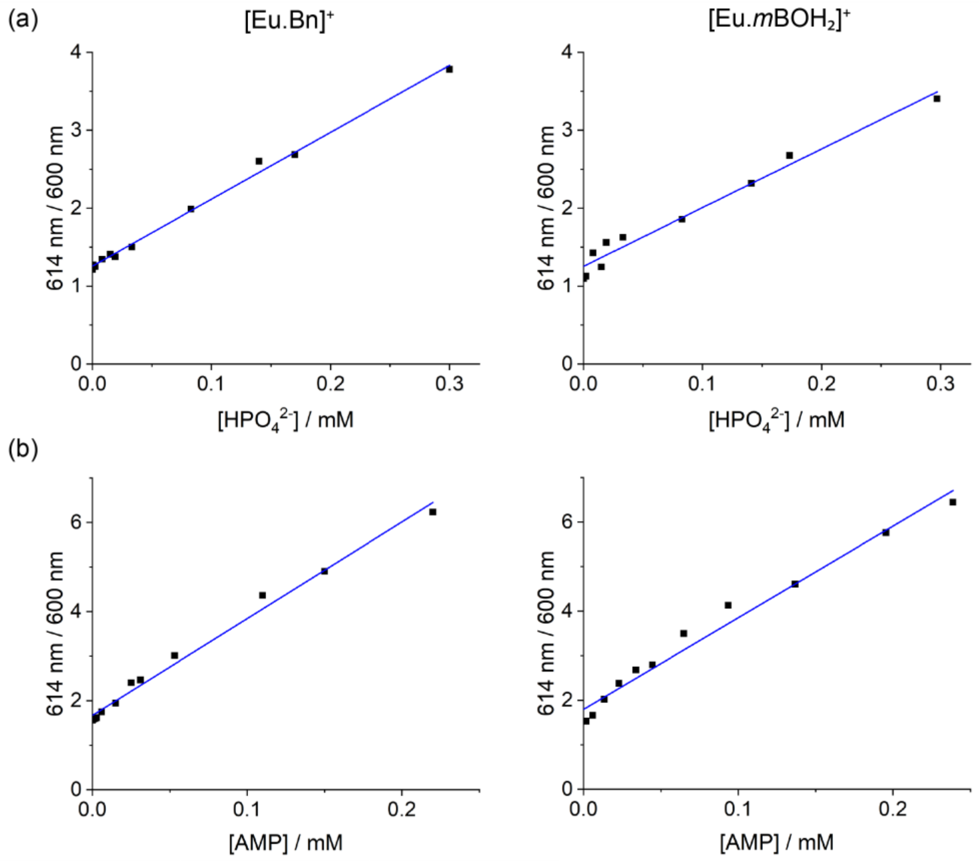

Figure S10. Linear increase in the emission intensity ratio of [**Eu.Bn**]+ and [**Eu.*m*BOH2**]+ with added (a) phosphate (0 – 300 μM) and (b) AMP (0–200 μM or above) measured in 10 mM HEPES buffer at pH 7.0. λ_{exc} = 322 nm, 295 K.

Table S2. Computed binding energies (kJ/mol) for water, phosphate, and AMP to the Eu(III) complexes.

	Binding energy (kJ/mol)[a]	
	[**Eu.Bn**]$^+$	[**Eu.mBOH2**]$^+$
H_2O	107	104
HPO_4^{2-}	340	350
AMP	364	375

[a] The binding energies are intended as a general measure of affinity. Evaluation of free energy differences needed to reproduce the binding constants in Table 2 of the main text is beyond the scope of this work, requiring consideration of entropic effects, solvation, conformational flexibility, and the influence of pH.

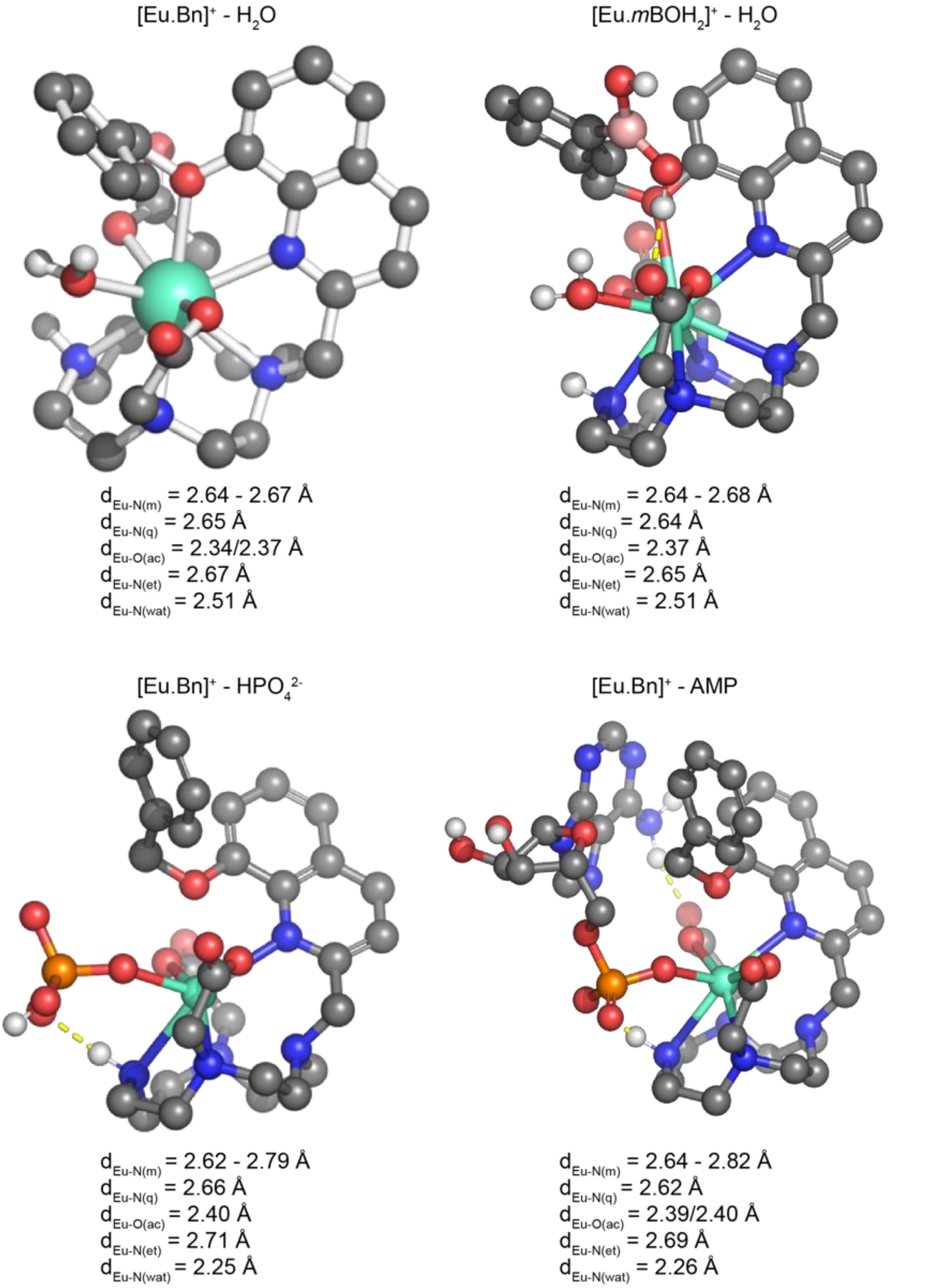

Figure S11. DFT-optimised molecular structures of europium(III) complexes bound to water, phosphate and AMP.

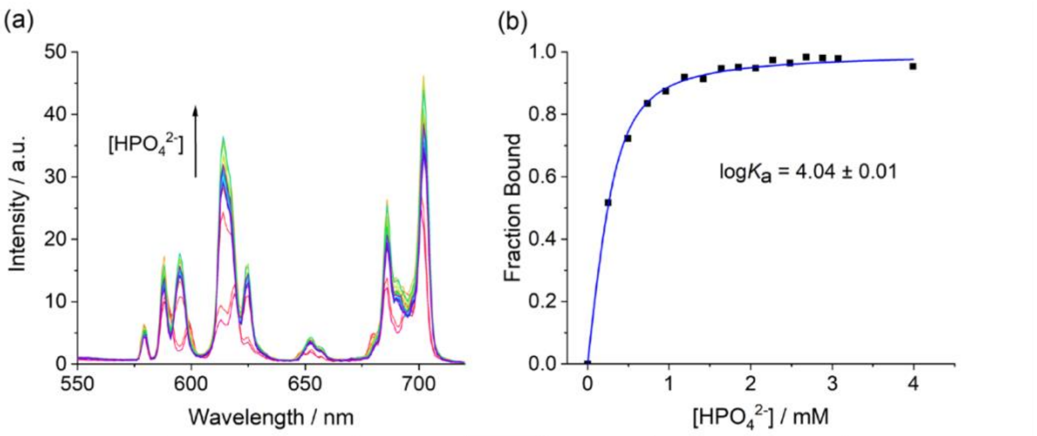

Figure S12. (a) Competition experiment showing the increase in emission spectra of [**Eu.Bn**]$^+$ (5 μM) upon addition of phosphate (0 – 4 mM) in a background of $NaHCO_3$ (27 mM). (b) Plot of fraction bound (determined from $\Delta J = 2$ / $\Delta J = 1$ ratio) versus phosphate concentration, showing the fit to a 1:1 binding isotherm. Measured in 10 mM HEPES at pH 7.0, containing 27 mM $NaHCO_3$, λ_{exc} = 322 nm, 295 K.

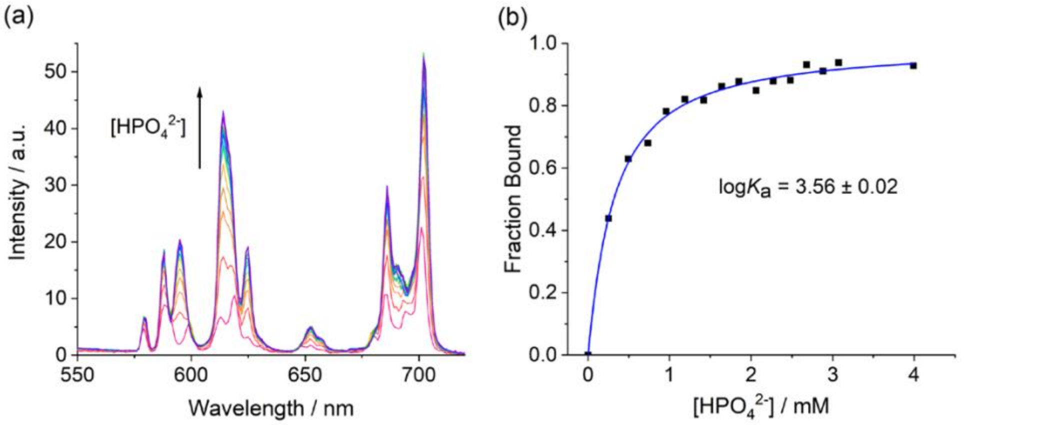

Figure S13. (a) Competition experiment showing the increase in emission spectra of [**Eu.Bn**]$^+$ (5 μM) upon addition of phosphate (0 – 4 mM) in a background of human serum albumin (0.4 mM). (b) Plot of fraction bound (determined from $\Delta J = 2$ / $\Delta J = 1$ ratio) versus phosphate concentration, showing the fit to a 1:1 binding isotherm. Measured in 10 mM HEPES at pH 7.0, containing 0.4 mM HSA, λ_{exc} = 322 nm, 295 K.

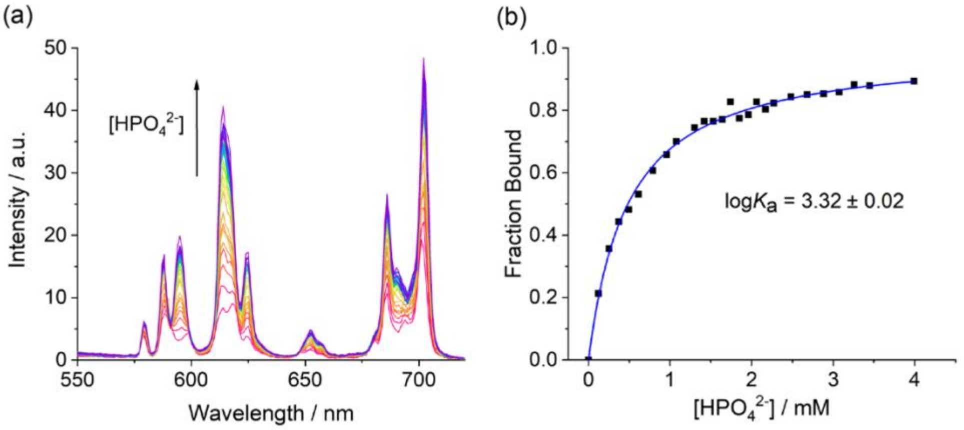

Figure S14. Selective detection of phosphate in a simulated extracellular fluid based on a Krebs saline solution, containing NaCl (145 mM), KCl (5 mM), $CaCl_2$ (2.5 mM), $MgCl_2$ (1.5 mM), $NaHCO_3$ (27 mM), Na_2SO_4 (0.5 mM), sodium lactate (1.0 mM), sodium citrate (0.15 mM), glucose (5.5 mM) and HSA (0.4 mM) in 10 mM HEPES buffer (pH 7.0). (a) Change in emission spectra of [**Eu.Bn**]$^+$ (5 μM) upon addition of phosphate (0 – 4 mM) (b) Plot of fraction bound (determined from ΔJ = 2 / ΔJ = 1 ratio) of [**Eu.Bn**]$^+$ (5 μM) versus phosphate concentration, showing the fit to a 1:1 binding isotherm. λ_{exc} = 322 nm, 295 K.

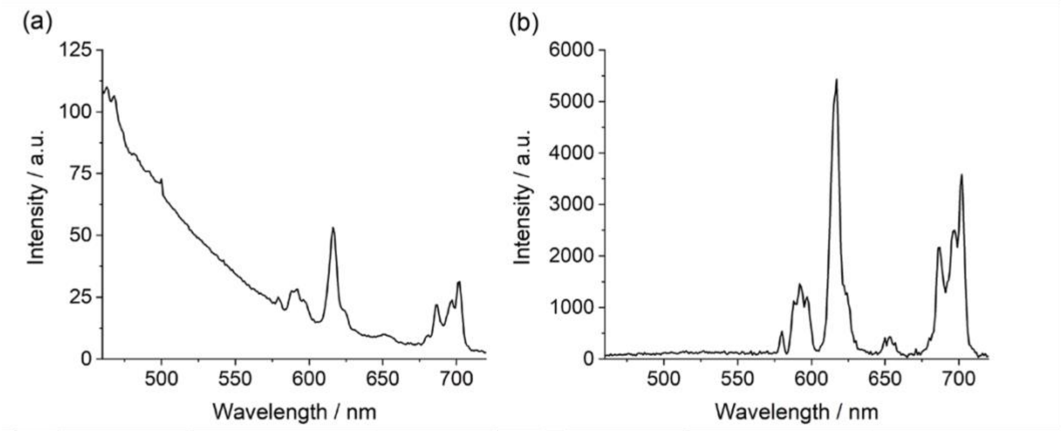

Figure S15. (a) Steady state emission spectrum of [**Eu.Bn**]$^+$ (20 μM) in human serum. (b) Time-resolved emission spectrum of [**Eu.Bn**]$^+$ (20 μM) in human serum, with protein fluorescence completely eliminated (integration time = 60 – 400 μs).

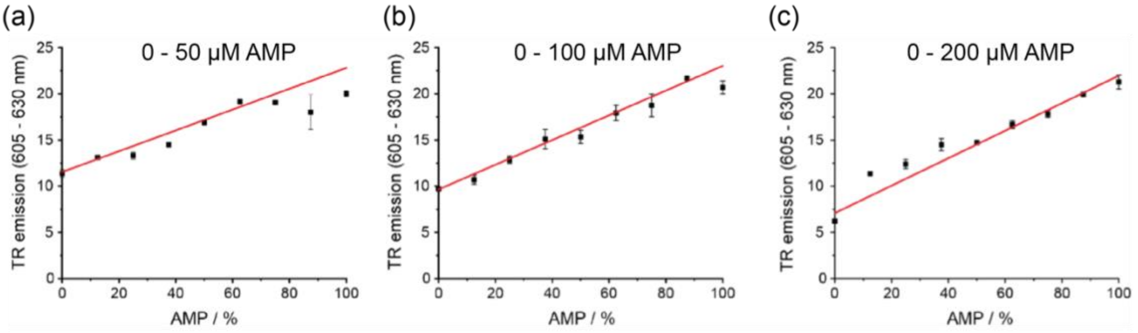

Figure S16. Microplate-based simulation of phosphodiesterase reactions (conversion of cAMP to AMP) at different total concentrations of [cAMP] + [AMP] including (a) 50 µM, (b) 100 µM and (c) 200 µM. Different ratios of cAMP / AMP were incubated with [**Eu.*m*BOH$_2$**]$^+$ (500 nM) in 10 mM HEPES at pH 7.0, containing 5 mM $MgCl_2$. The time-resolved emission intensity of [**Eu.*m*BOH$_2$**]$^+$ was recorded (λ_{ex} = 292 – 366 nm, λ_{em} = 605 – 630 nm, integration time = 60 – 400 µs) as the percentage of AMP increased.

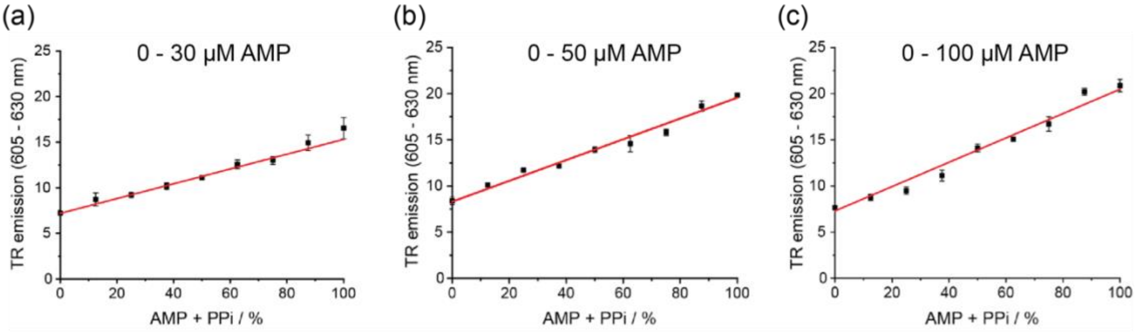

Figure S17. Microplate-based simulation of aminoacyl tRNA synthetase reactions (conversion of ATP to AMP + PPi) at different total concentrations of [ATP] + [AMP] + [PPi] including (a) 30 µM, (b) 50 µM and (c) 100 µM. Different ratios of ATP / (AMP + PPi) were incubated with [**Eu.*m*BOH$_2$**]$^+$ (500 nM) in 10 mM HEPES at pH 7.0, containing all 20 natural amino acids (100 µM each), DTT (1 mM), KCl (10 mM), $MgCl_2$ (5 mM). The time-resolved emission intensity of [**Eu.*m*BOH$_2$**]$^+$ was recorded (λ_{ex} = 292 – 366 nm, λ_{em} = 605 – 630 nm, integration time = 60 – 400 µs) as the percentage of AMP + PPi increased.

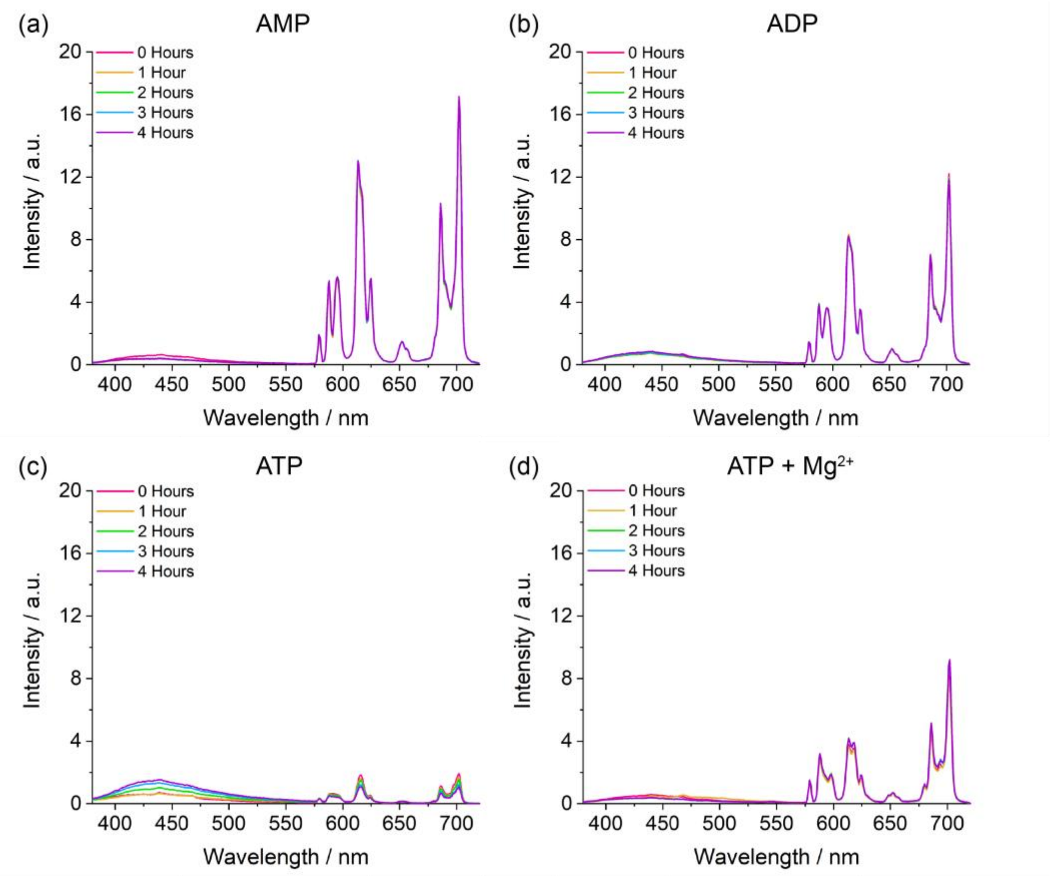

Figure S18. Stability of the emission response of [**Eu.*m*BOH**$_2$]$^+$ (5 µM) over a 4 hour incubation period with (a) 1 mM AMP, (b) 1 mM ADP, (c) 1 mM ATP, (d) 1 mM ATP + 5 mM Mg^{2+}, measured in 10 mM HEPES buffer at pH 7.0. λ_{exc} = 322 nm, 295 K.

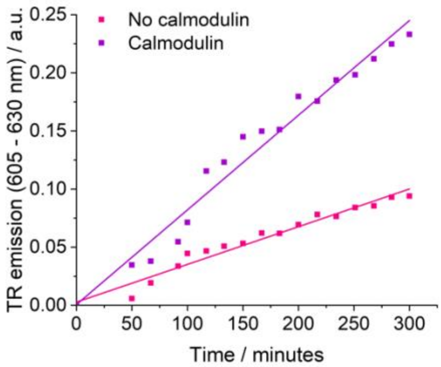

Figure S19. Real-time monitoring of the PDE-catalysed conversion of cAMP to AMP using the time-resolved luminescence of [**Eu.*m*BOH**$_2$]$^+$, highlighting the linear regions of the reaction profiles to compare the change in rate upon activation with calmodulin (2 µM) and $CaCl_2$ (60 µM). Conditions: 200 µM cAMP, 4 µL PDE (0.1 mg mL^{-1}), 5 mM $MgCl_2$, 10 µM [**Eu.*m*BOH**$_2$]$^+$, 10 mM HEPES at pH 7.0, λ_{exc} = 292 – 366 nm, λ_{em} = 615 – 625 nm, integration time = 60 – 400 µs.

NMR Spectra

8-Hydroxyquinoline-2-carbaldehyde (1)

^{1}H NMR (400 MHz, $CDCl_3$, 298 K)

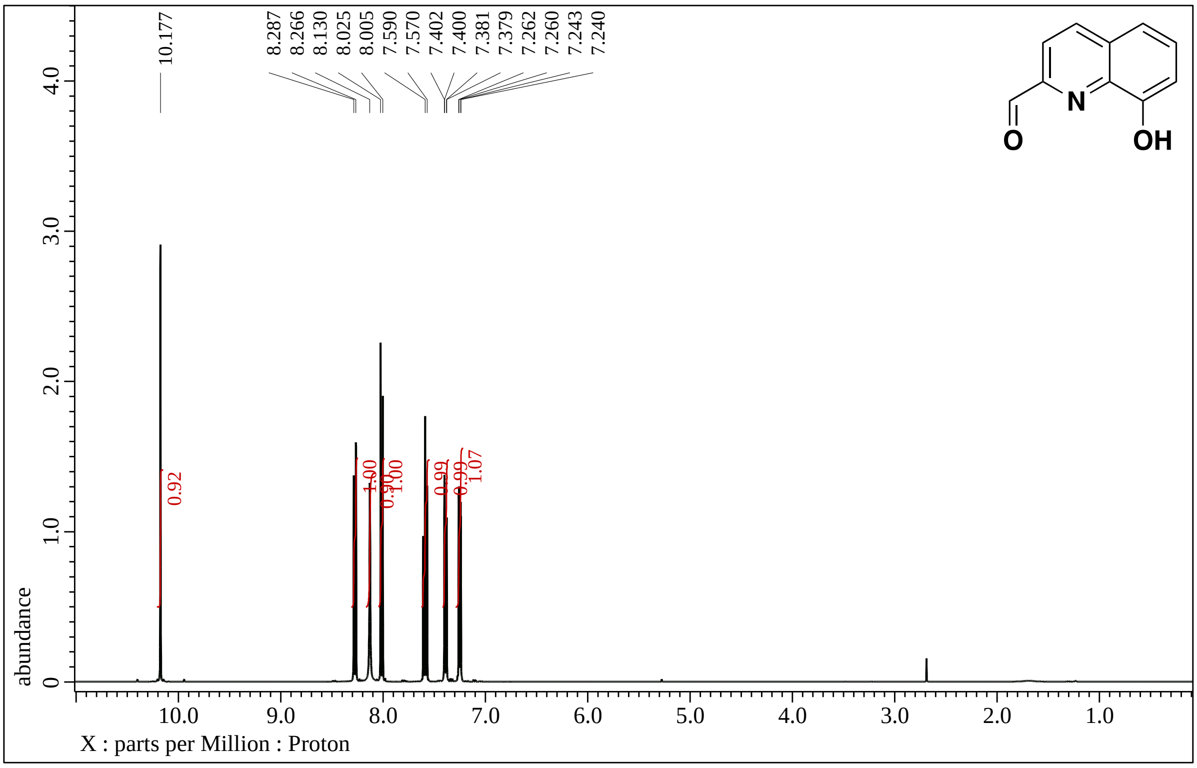

^{13}C NMR (100 MHz, $CDCl_3$, 298 K)

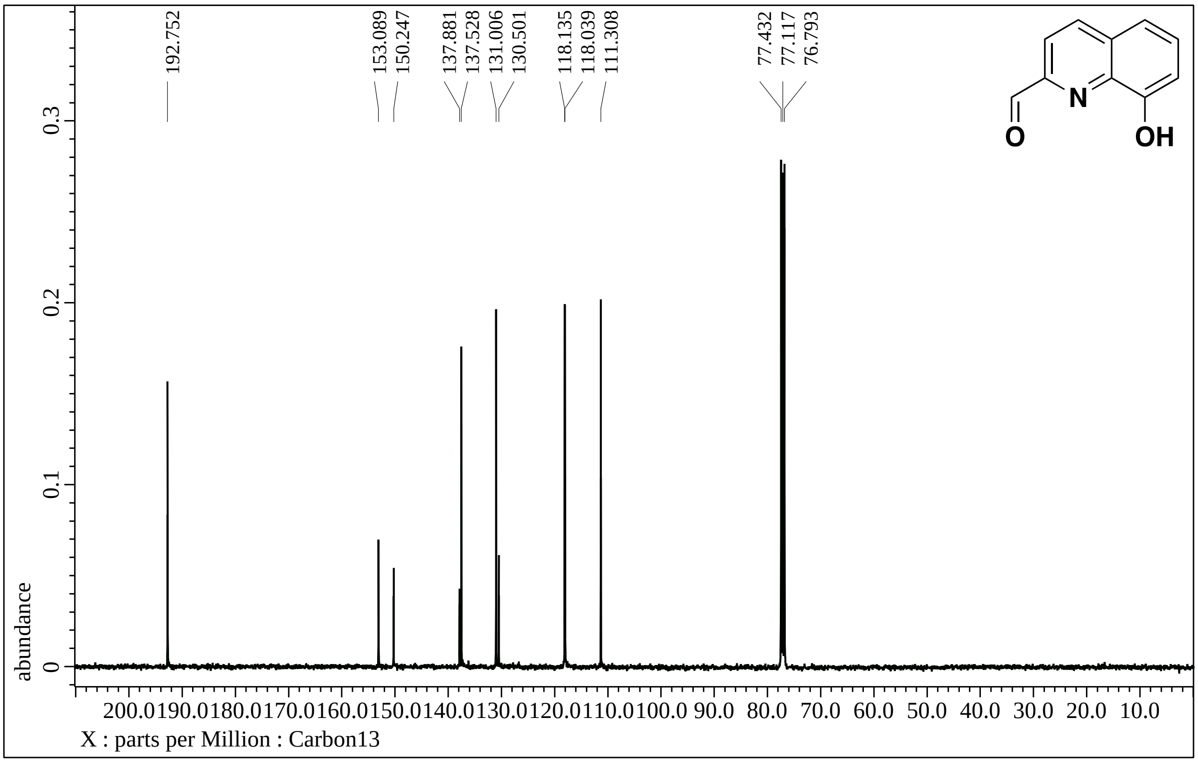

COSY (400 MHz, $CDCl_3$, 298 K) displayed range: 6.5 ppm – 10.5 ppm

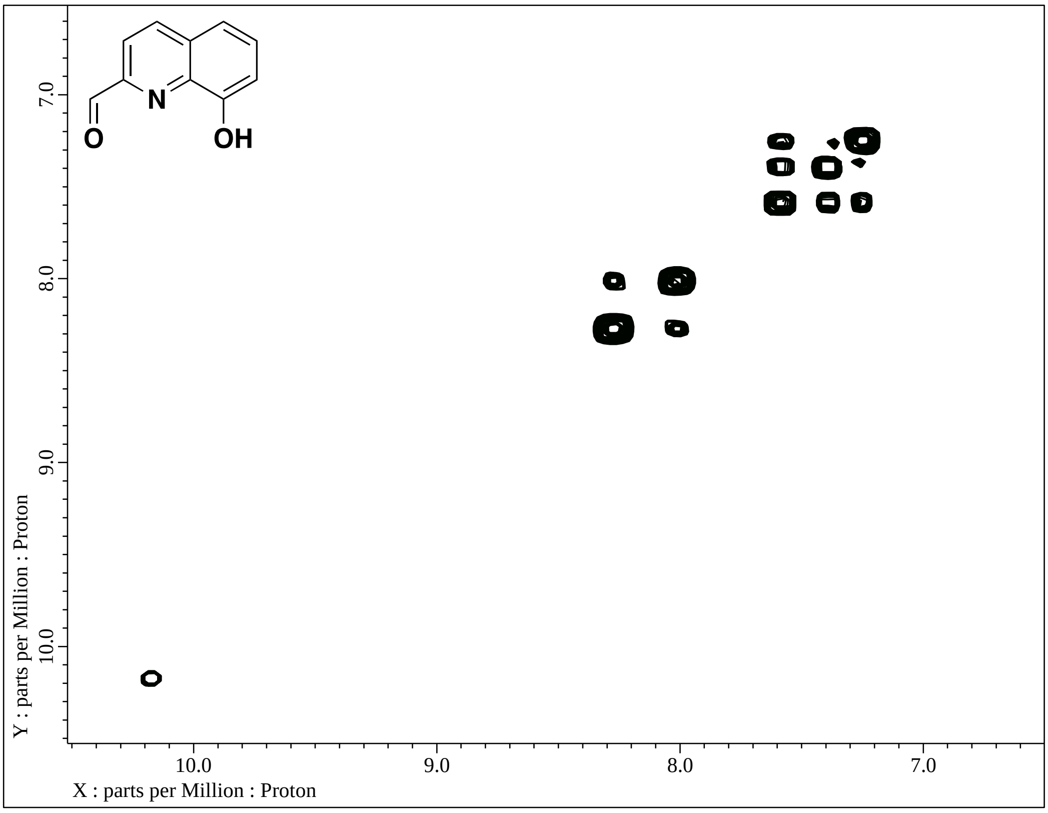

HSQC (400 MHz, $CDCl_3$, 298 K)

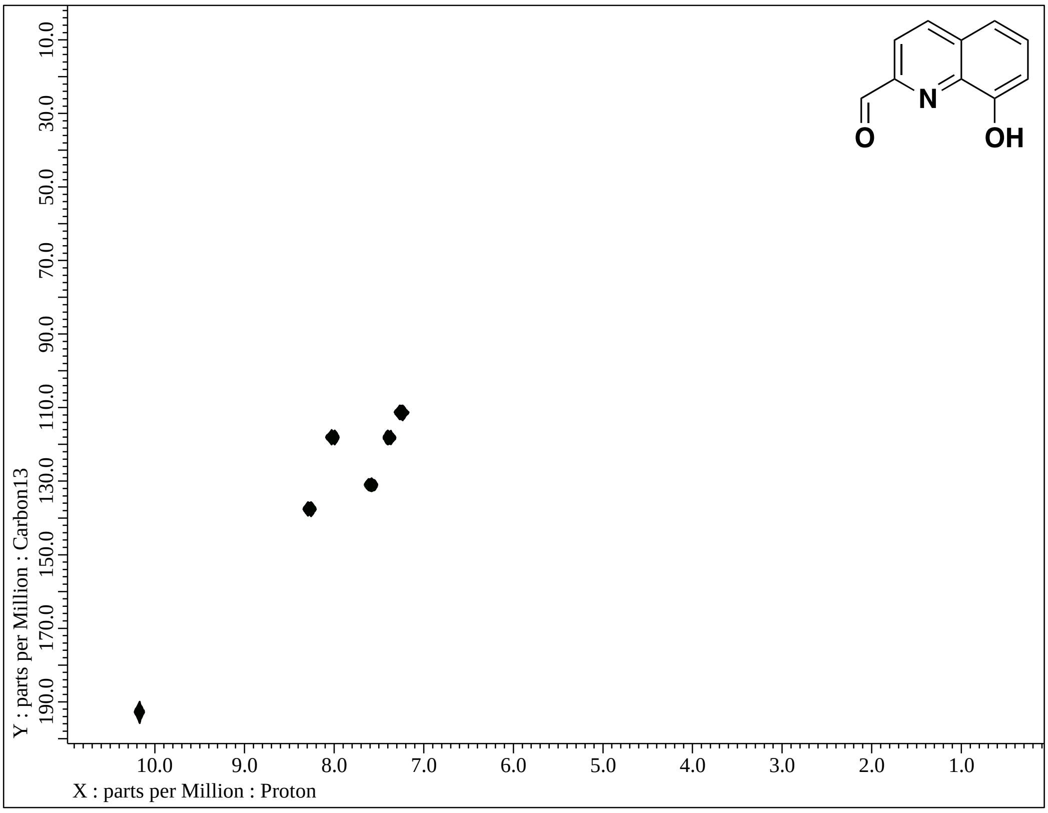

HMBC (400 MHz, $CDCl_3$, 298 K)

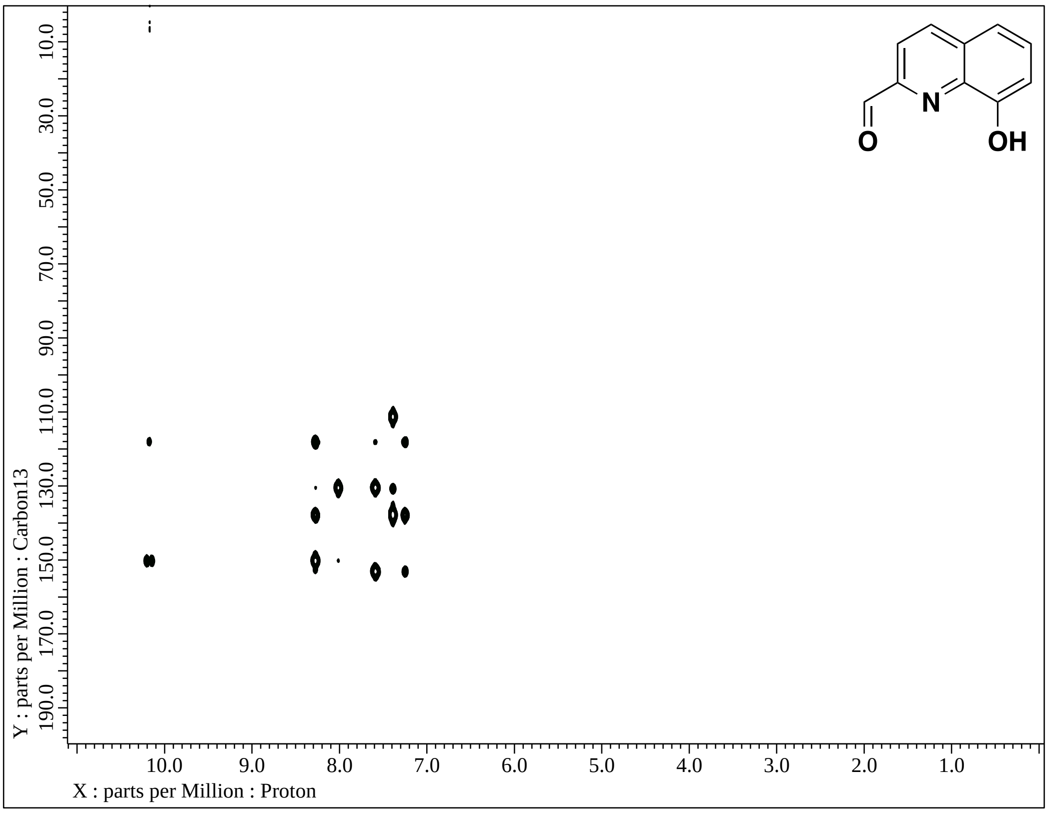

8-((Phenylmethoxy)-2-quinoline)-carbaldehyde (2)

^{1}H NMR (400 MHz, $CDCl_3$, 298 K)

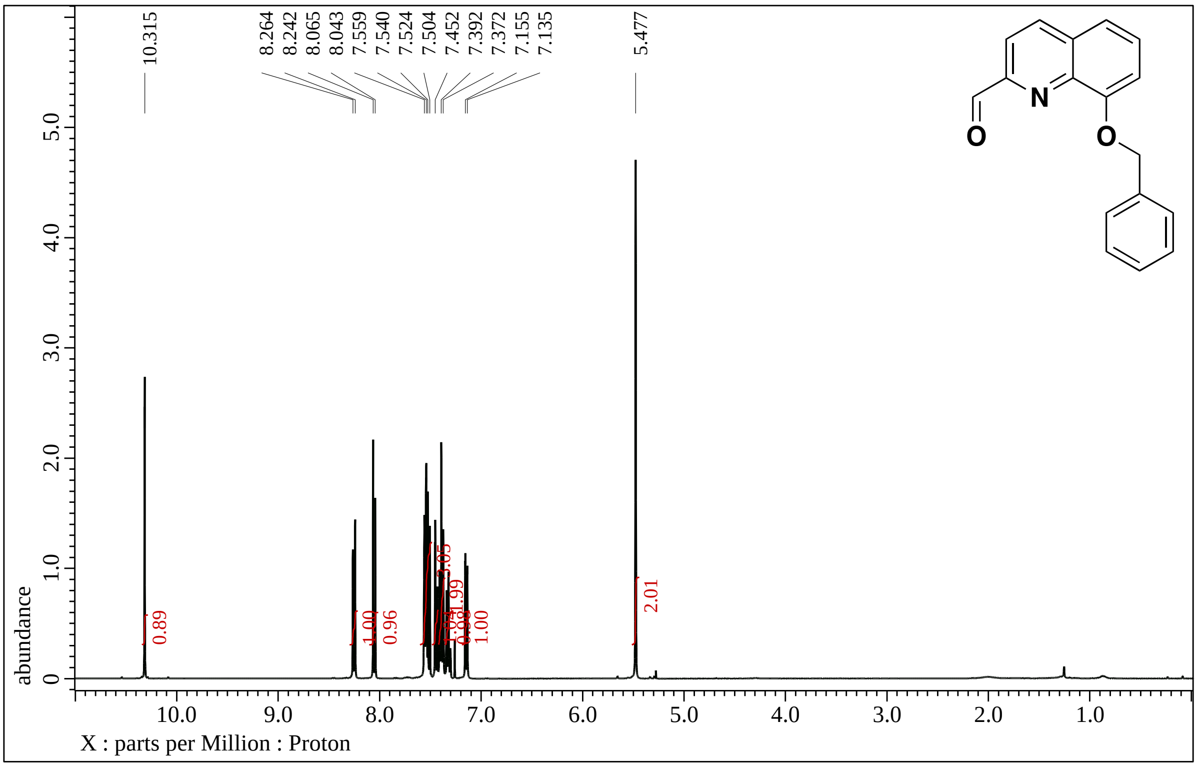

^{13}C NMR (100 MHz, $CDCl_3$, 298 K)

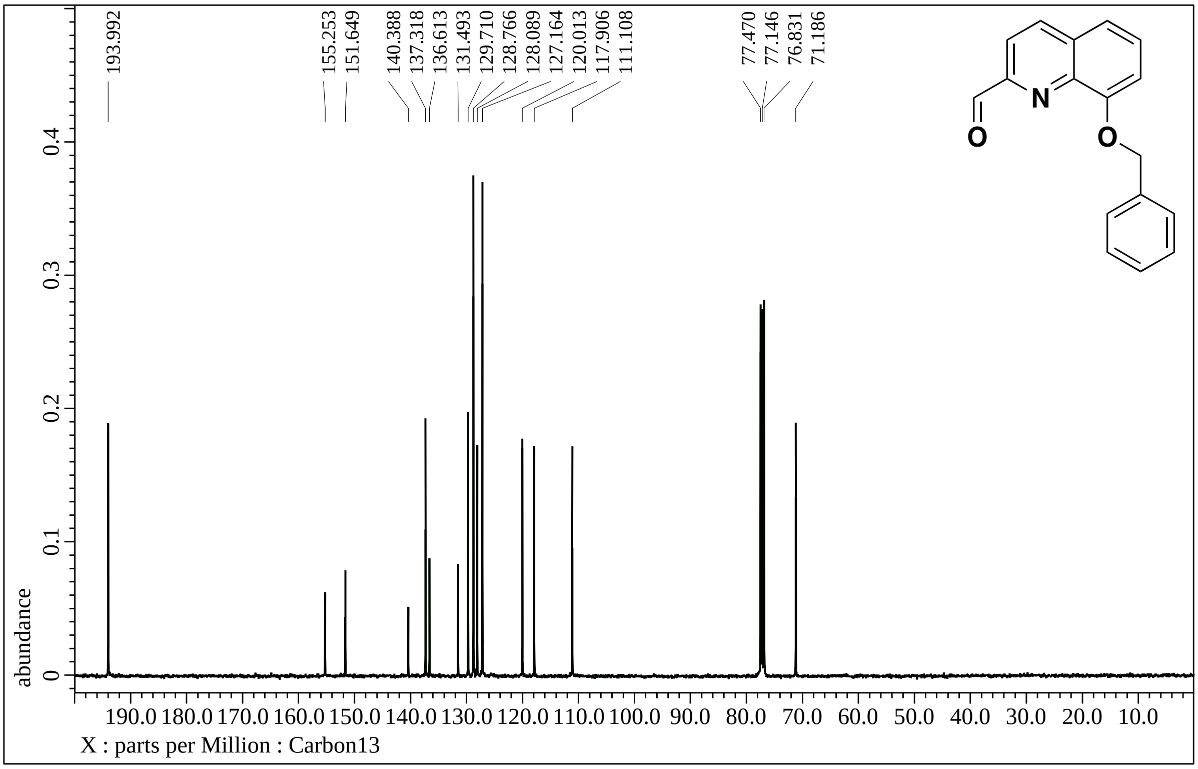

COSY (400 MHz, $CDCl_3$, 298 K) displayed range 5.5 ppm – 10.5 ppm

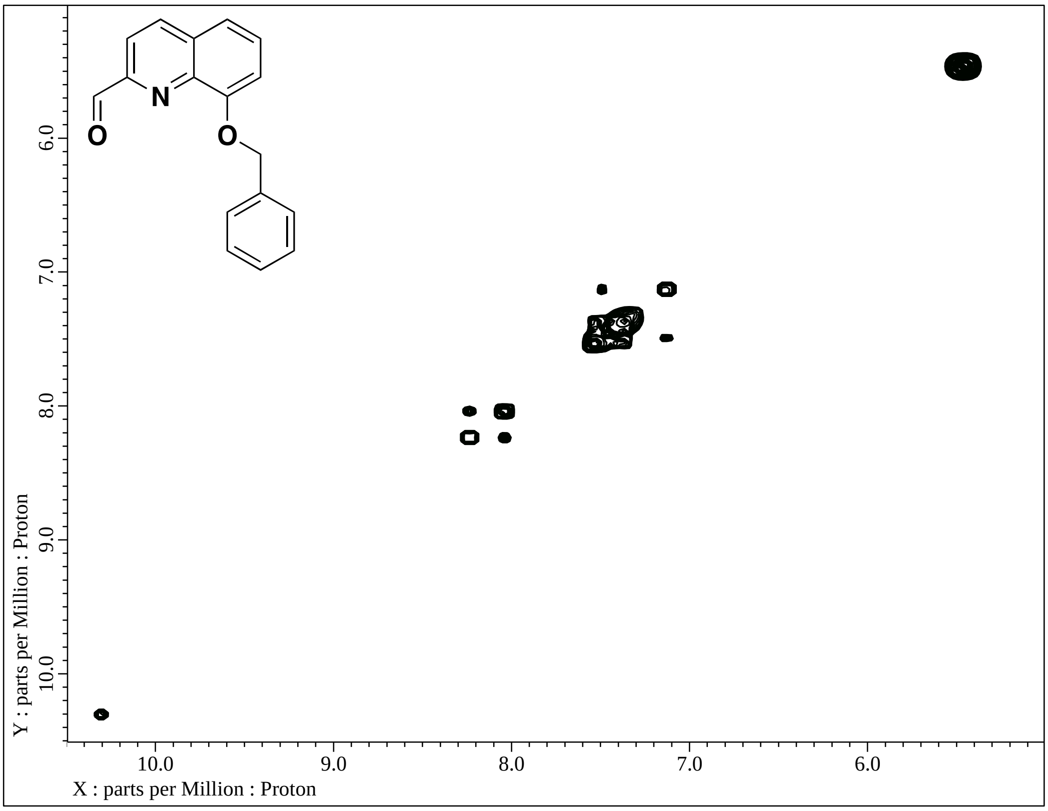

HSQC (400 MHz, $CDCl_3$, 298 K)

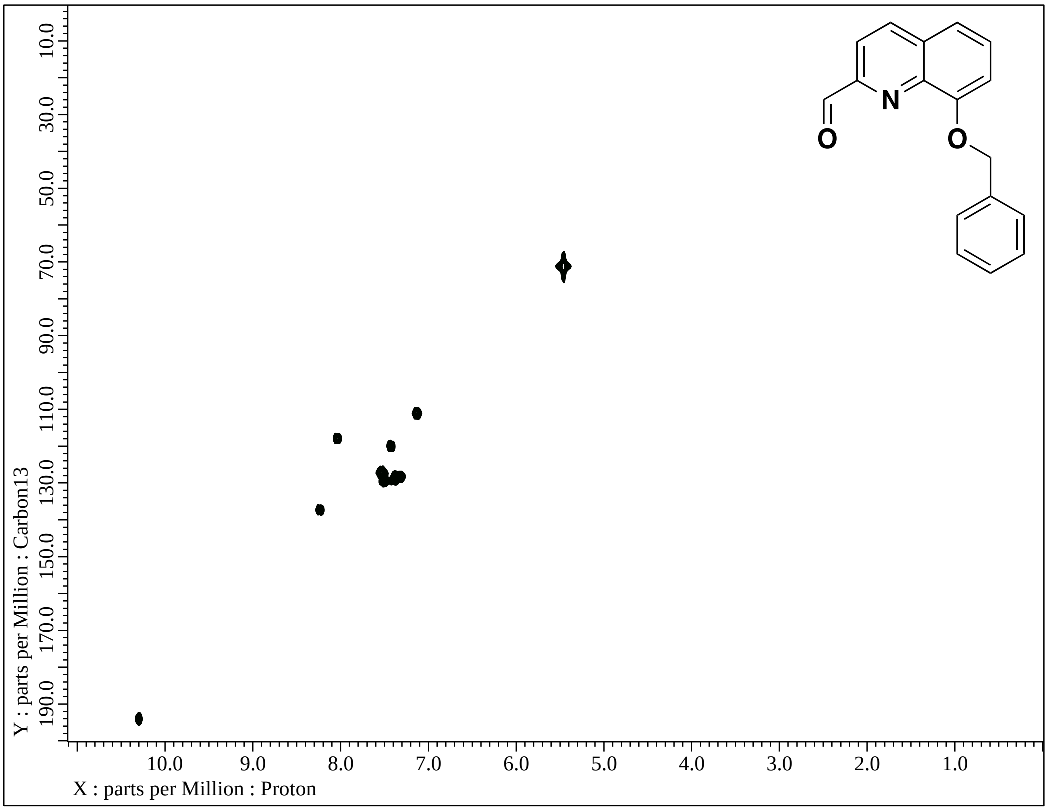

HMBC (400 MHz, $CDCl_3$, 298 K)

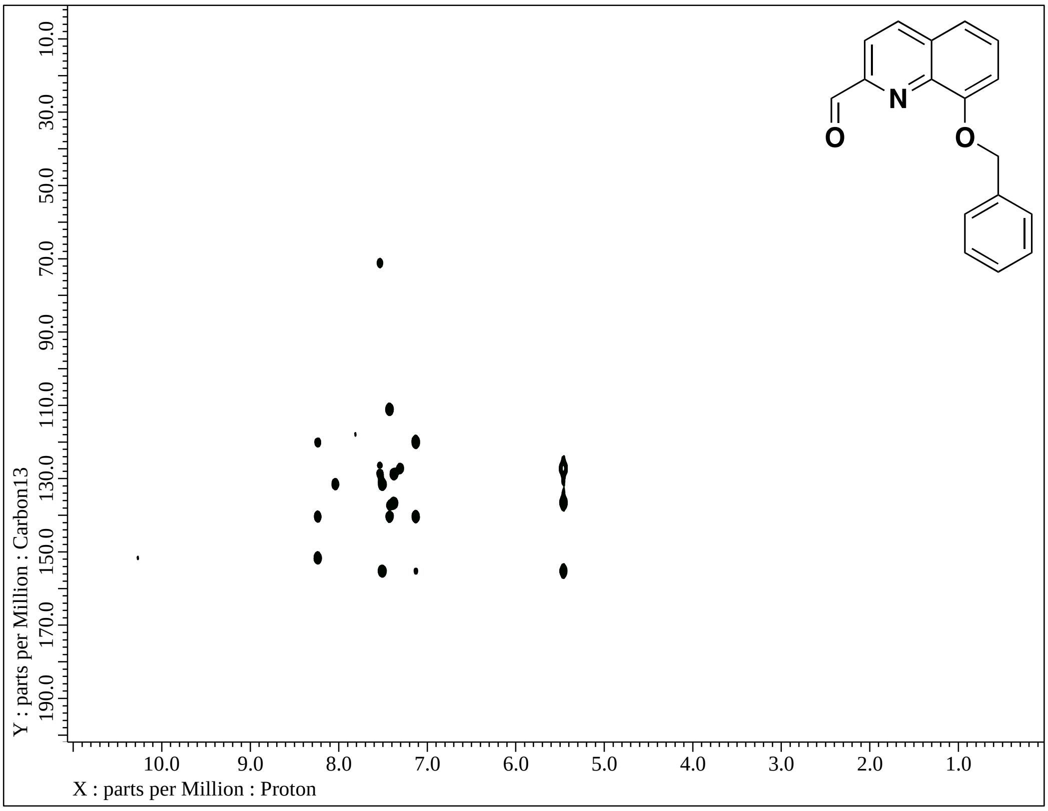

8-((Phenylmethoxy)-2-quinoline)methanol (3)

^{1}H NMR (400 MHz, $CDCl_3$, 298 K)

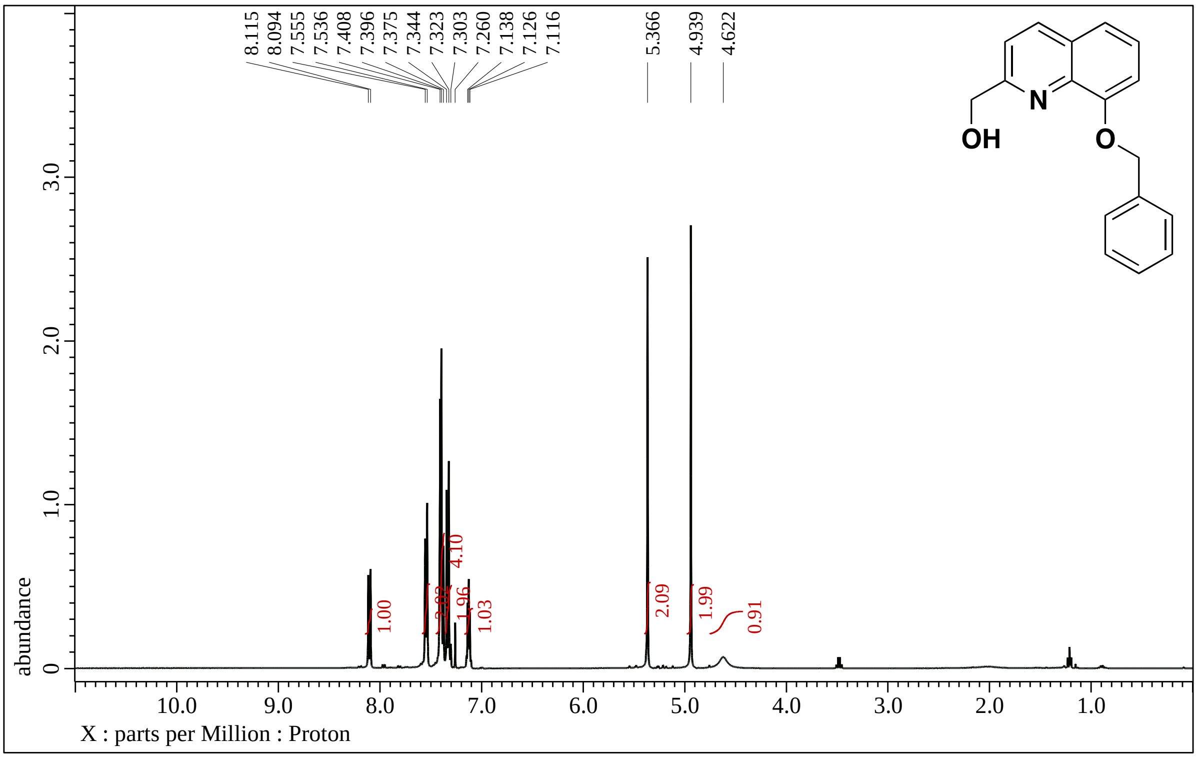

^{13}C NMR (100 MHz, $CDCl_3$, 298 K)

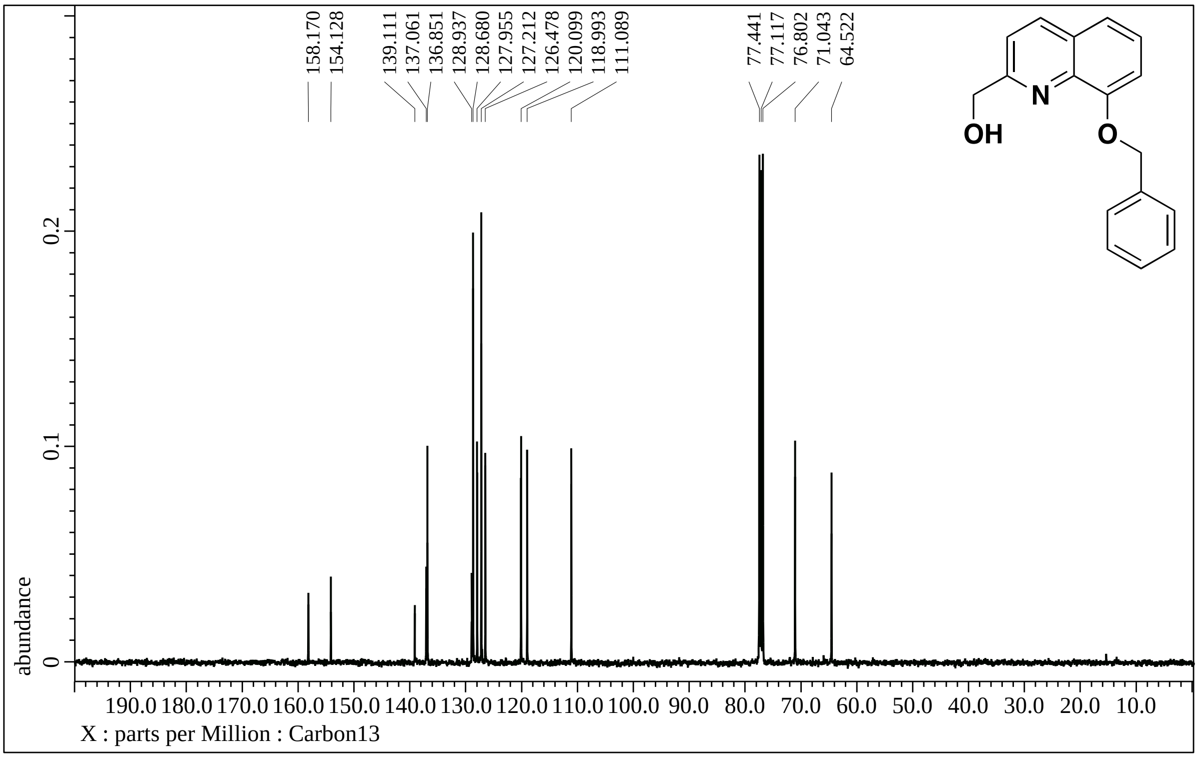

COSY (400 MHz, $CDCl_3$, 298 K) displayed range 4.0 ppm – 9.0 ppm

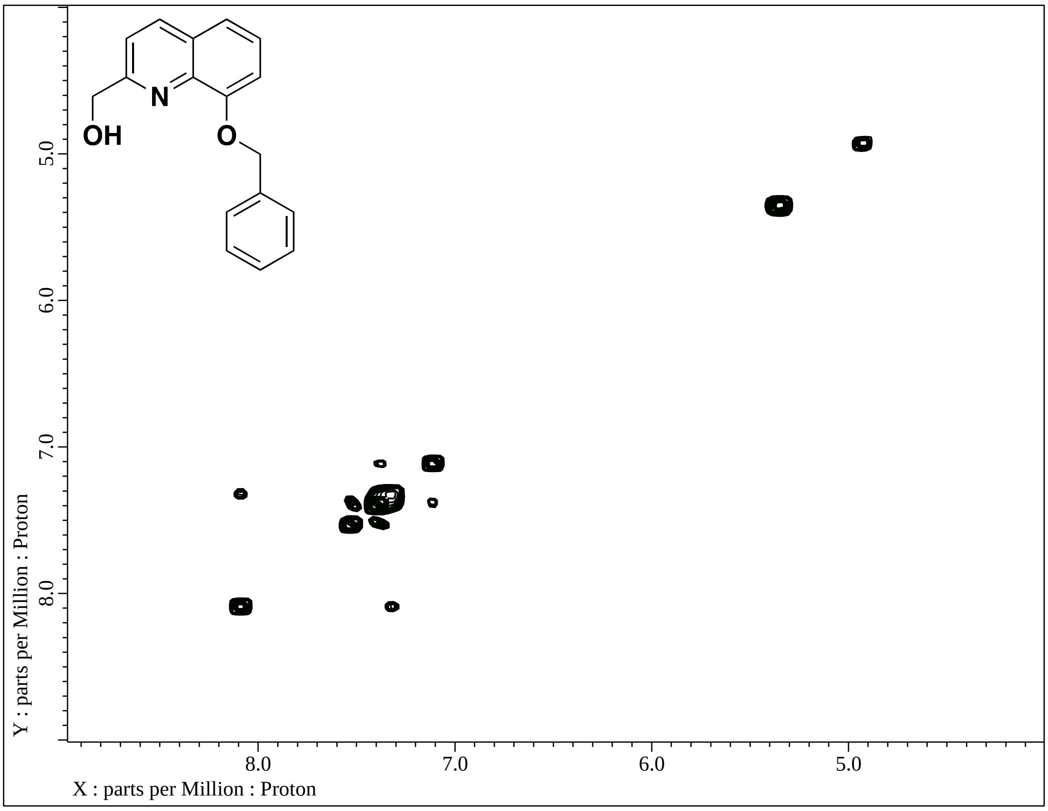

HSQC (400 MHz, $CDCl_3$, 298 K)

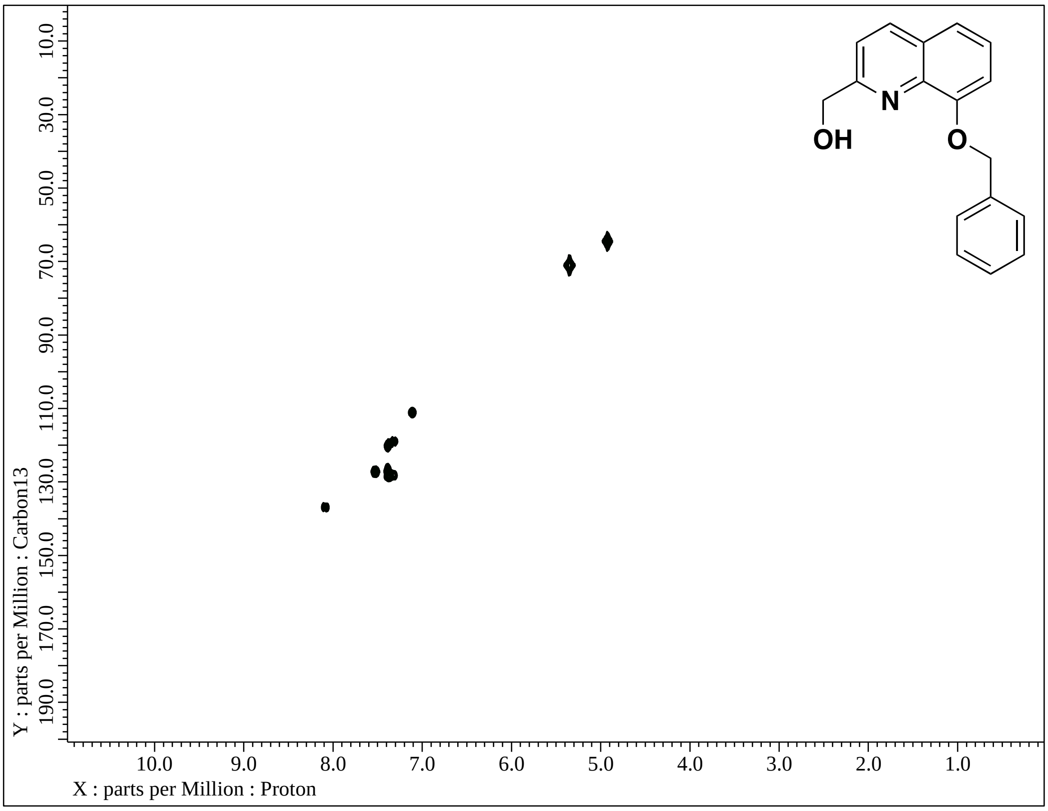

HMBC (400 MHz, $CDCl_3$, 298 K)

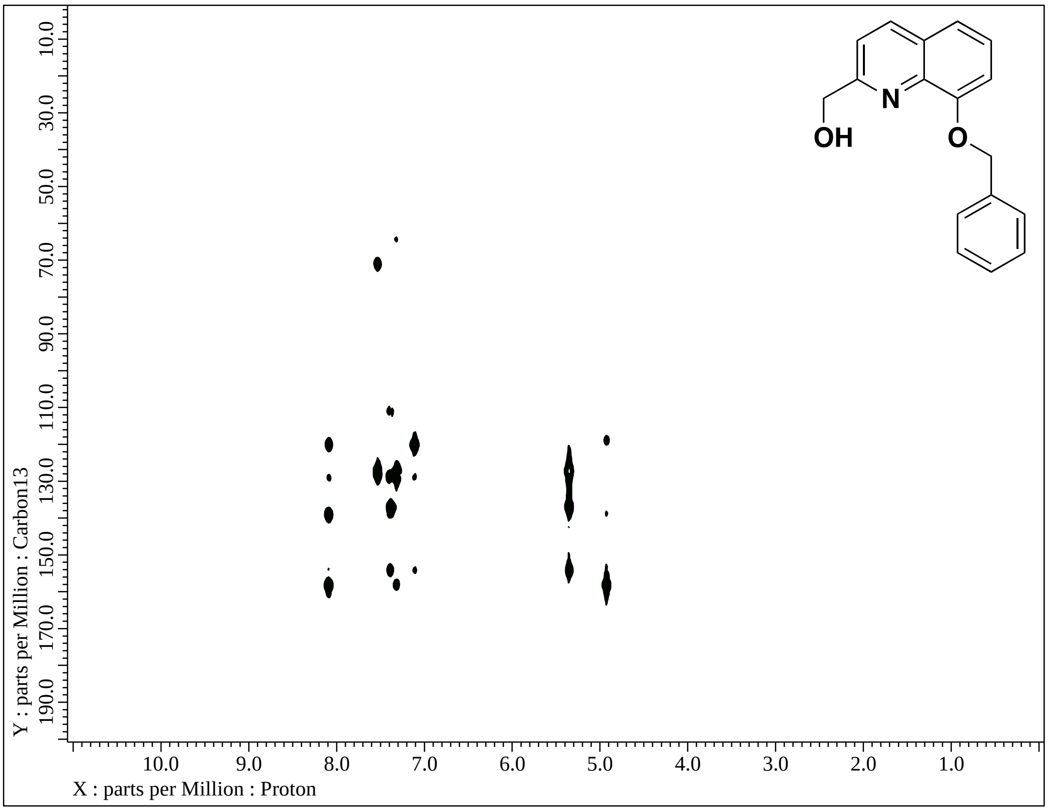

(8-((Phenylmethoxy)-2-quinoline)-methanesulfonate (4)

^{1}H NMR (400 MHz, $CDCl_3$, 298 K)

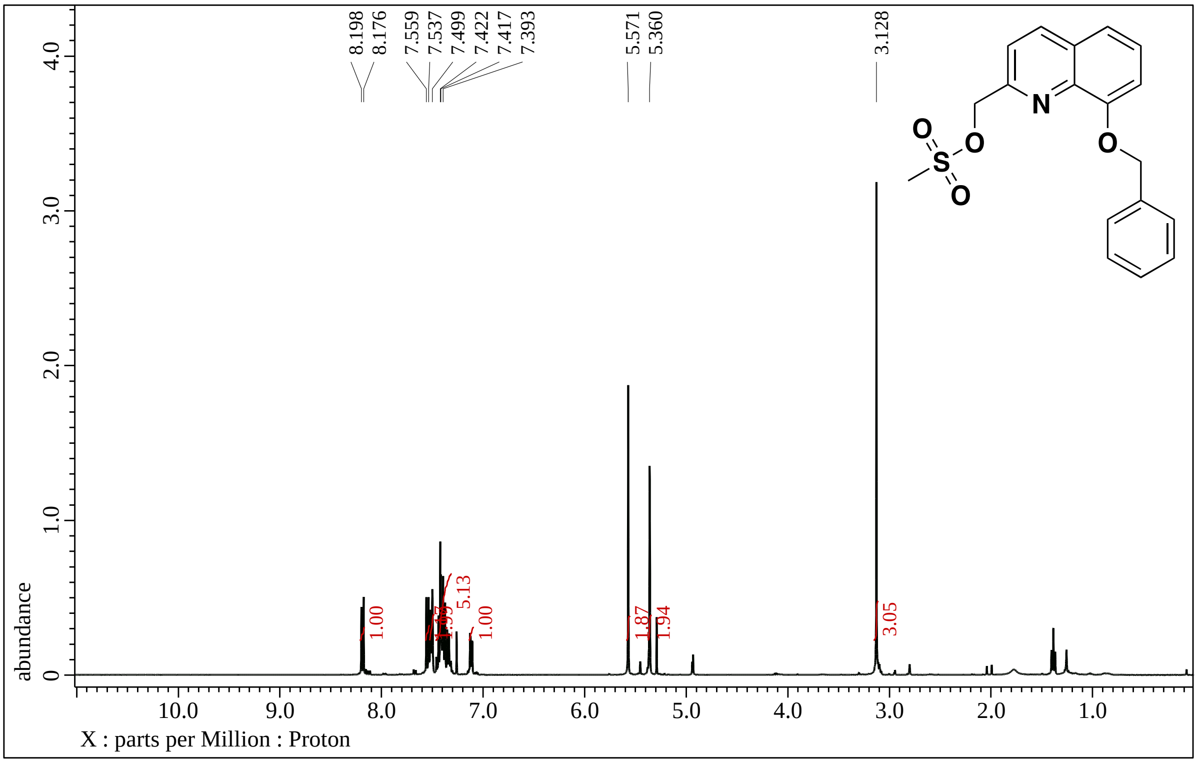

^{13}C NMR (100 MHz, $CDCl_3$, 298 K)

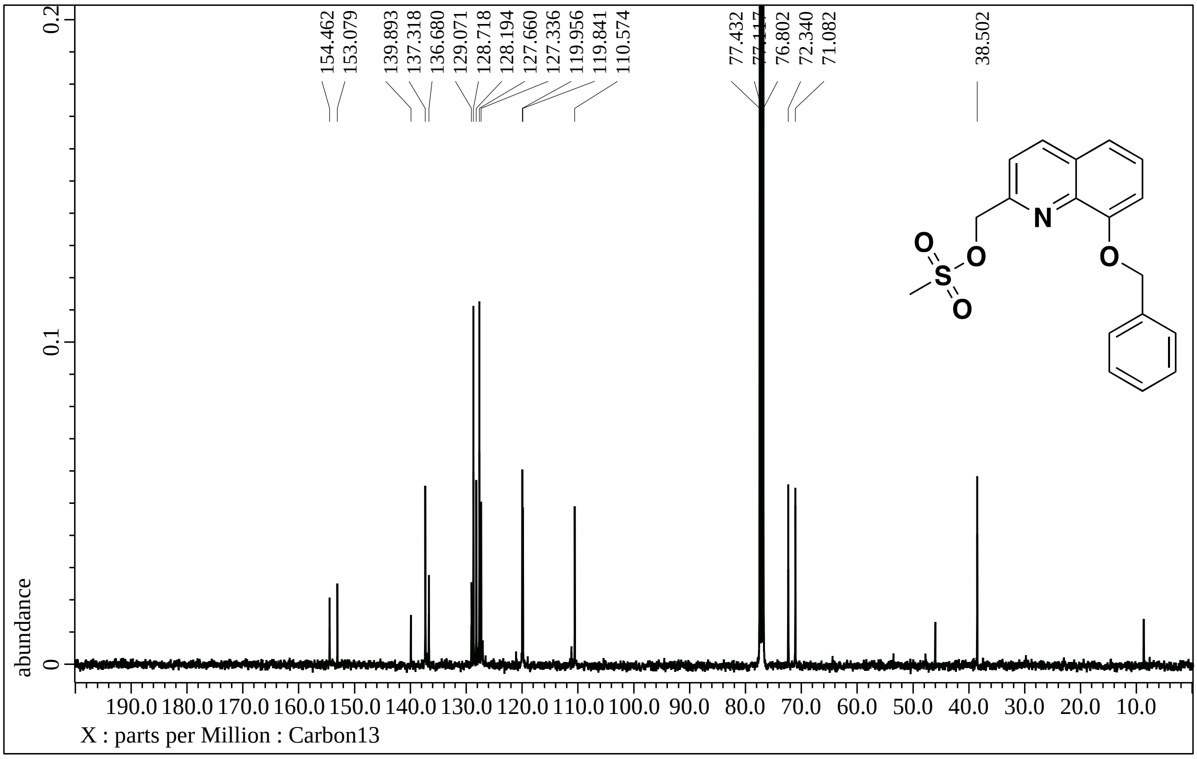

COSY (400 MHz, $CDCl_3$, 298 K) displayed range 6.5 ppm – 10.5 ppm

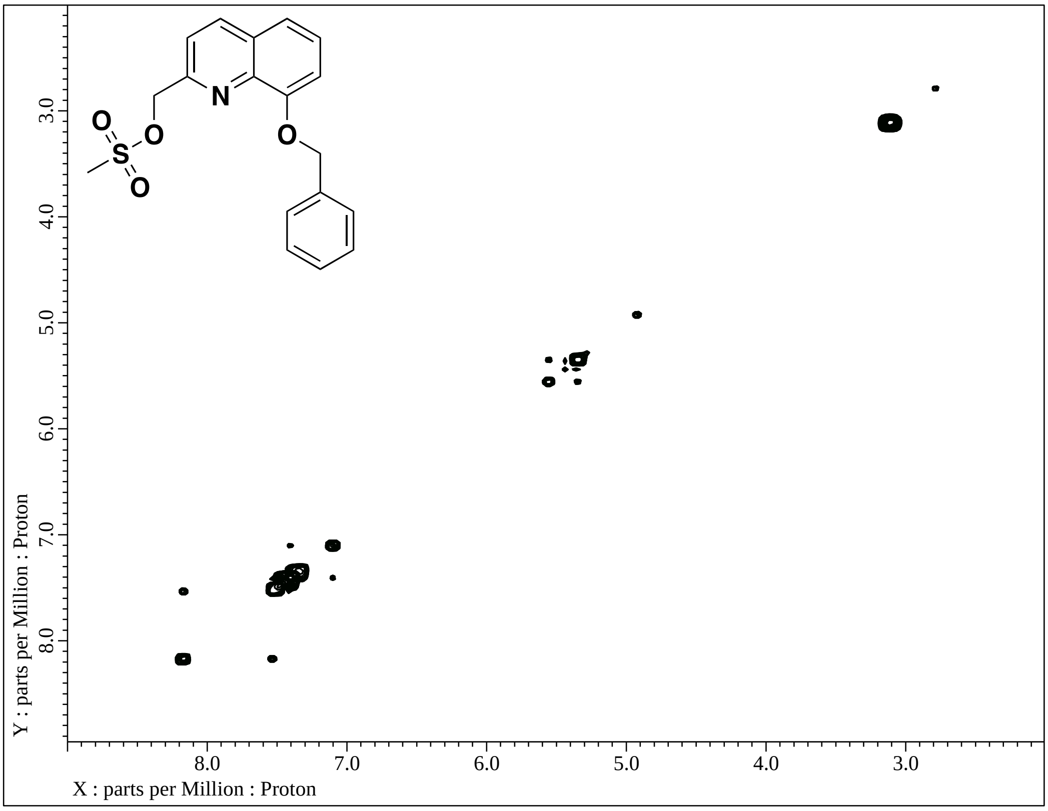

HSQC (400 MHz, $CDCl_3$, 298 K)

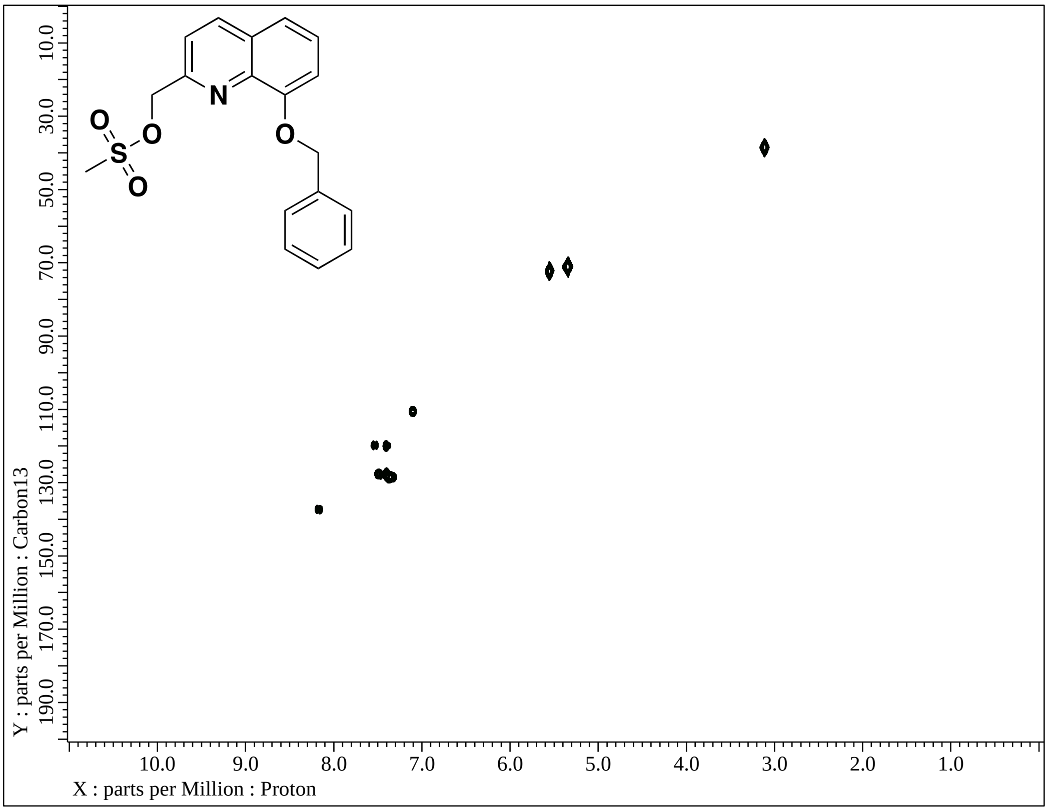

HMBC (400 MHz, $CDCl_3$, 298 K)

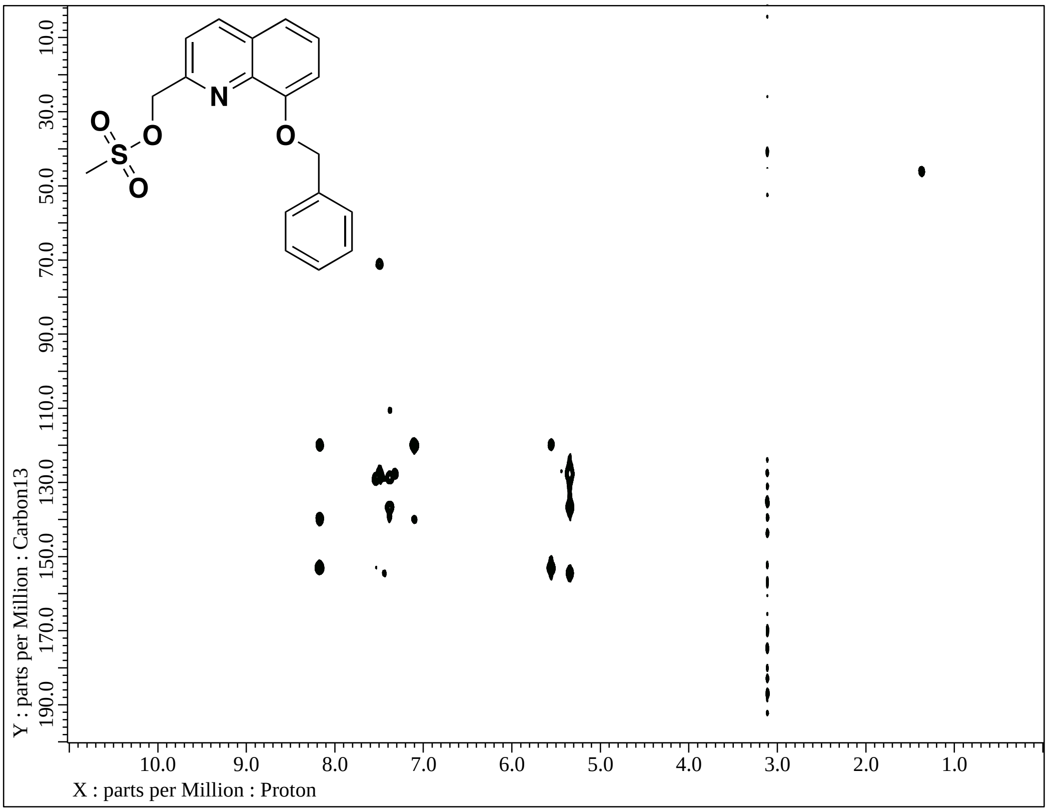

Di-*tert*-butyl 2,2'-(4-((8-(phenylmethoxy)-2-quinoline)methyl)-1,4,7,10-tetraazacyclododecane-1,7-diyl)diacetate (5)

^{1}H NMR (400 MHz, $CDCl_3$, 298 K)

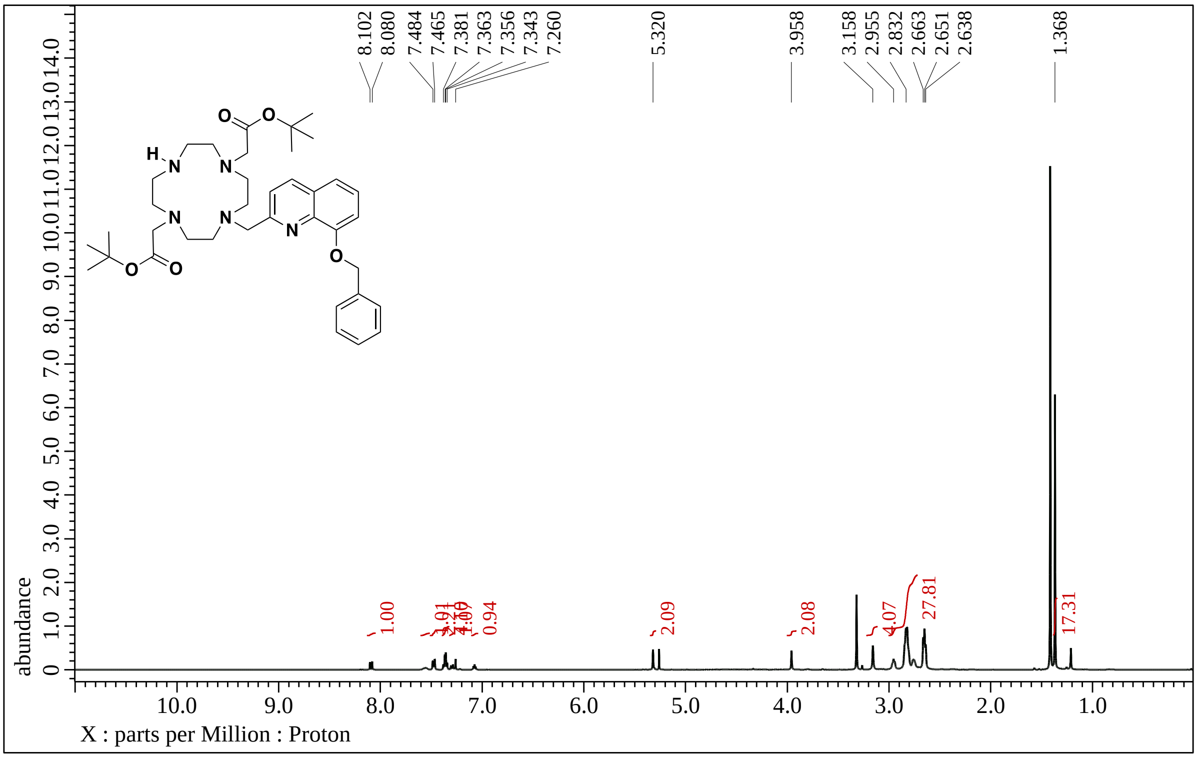

^{13}C NMR (100 MHz, $CDCl_3$, 298 K)

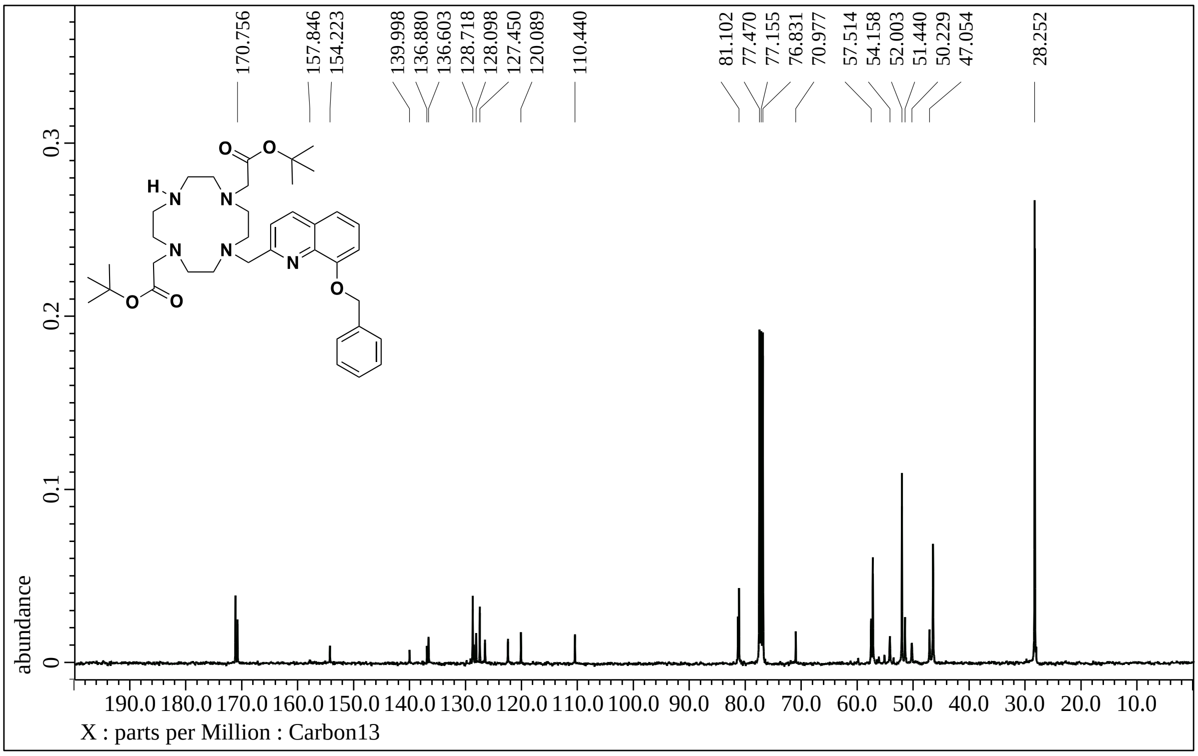

COSY (400 MHz, $CDCl_3$, 298 K) displayed range 1.0 ppm – 9.0 ppm

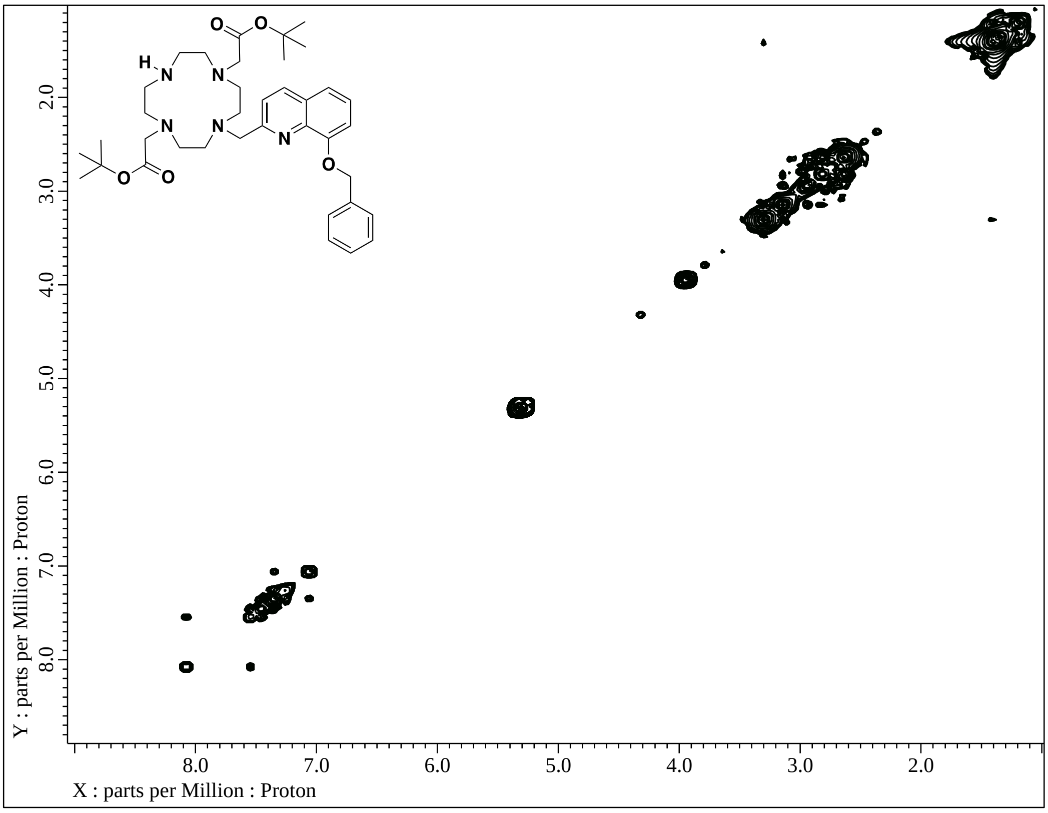

HSQC (400 MHz, $CDCl_3$, 298 K)

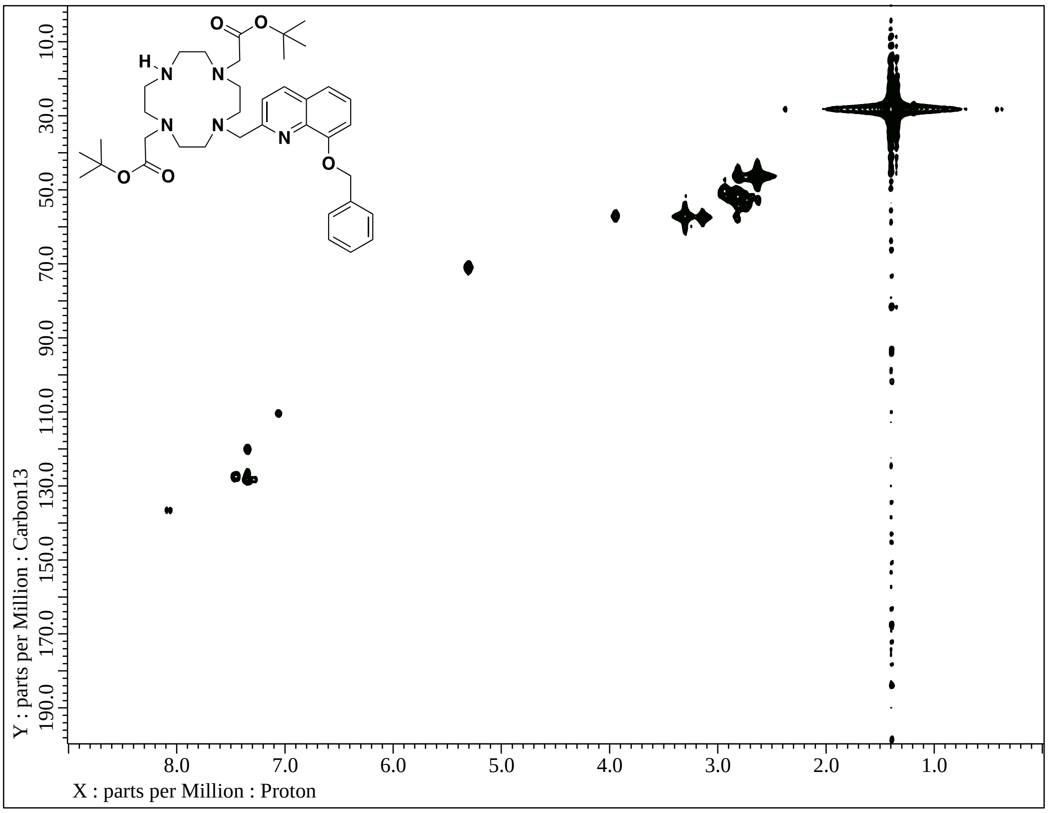

HMBC (400 MHz, $CDCl_3$, 298 K)

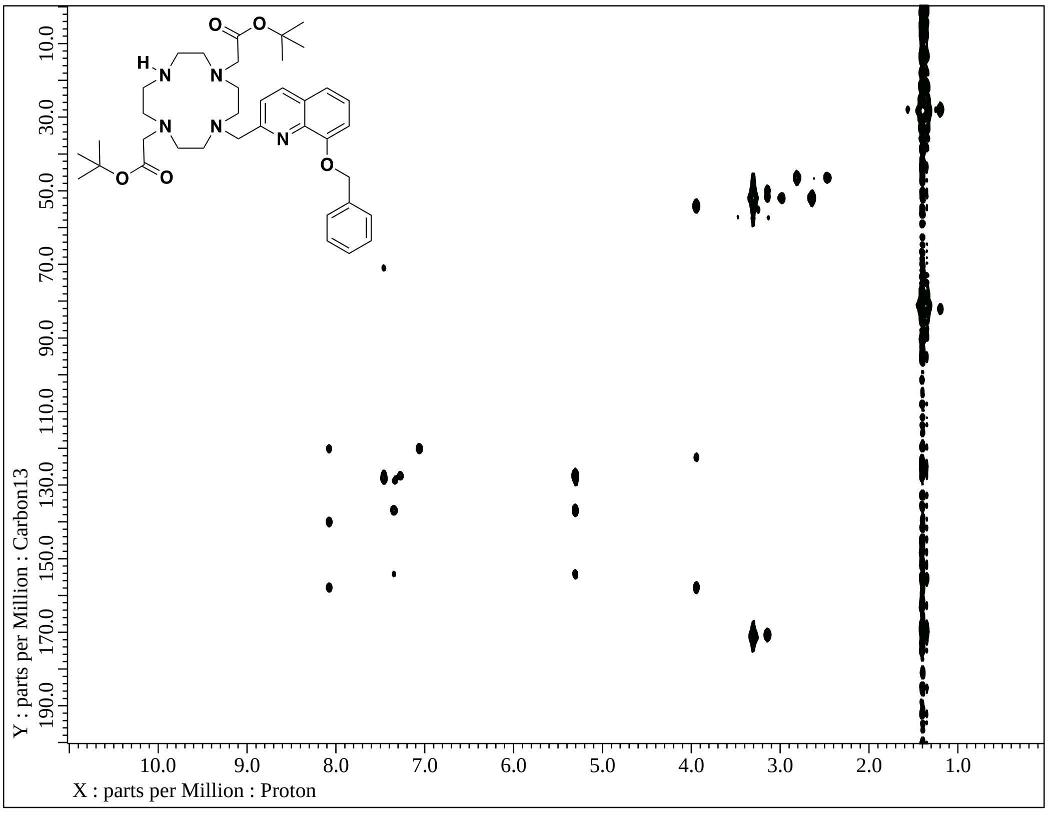

2,2'-(4-((8-(phenylmethoxy)-2-quinoline)-methyl)-1,4,7,10-tetraazacyclododecane-1,7-diyl)diacetic acid (6)

^1H NMR (400 MHz, methanol- d_4, 298 K)

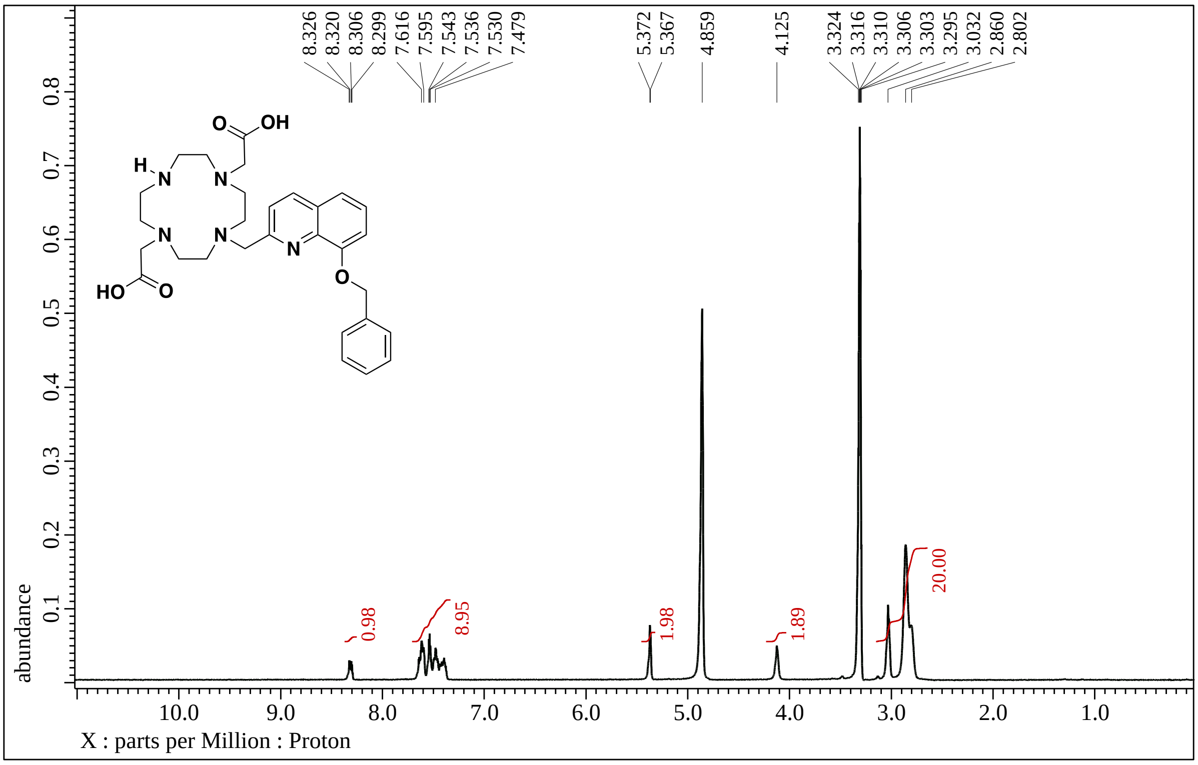

^{13}C NMR (100 MHz, methanol- d_4, 298 K)

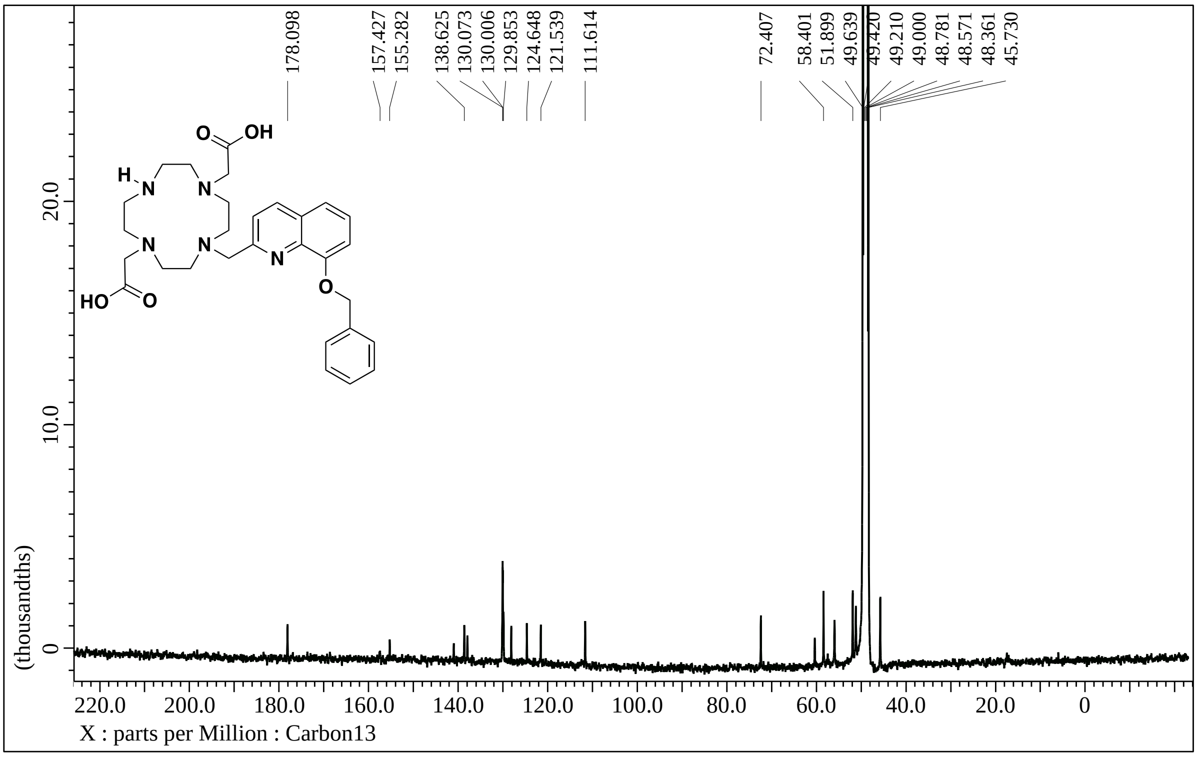

COSY (400 MHz, methanol- d_4, 298 K) displayed range 6.5 ppm – 10.5 ppm

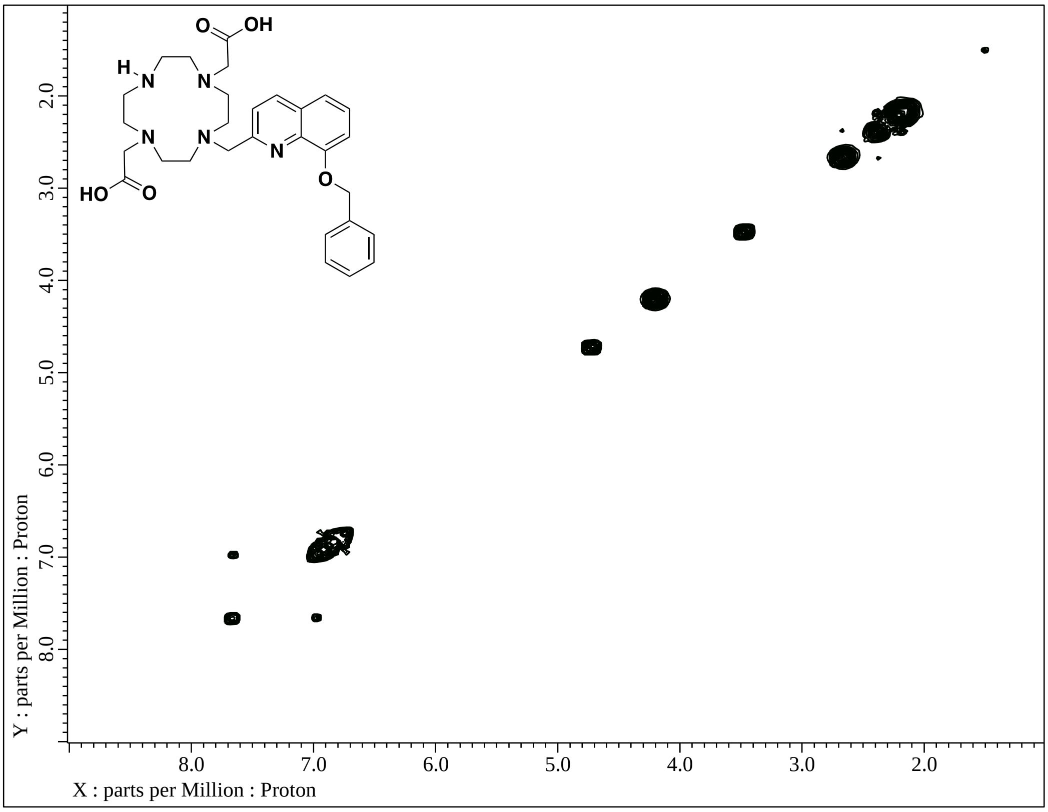

HSQC (400 MHz, methanol- d_4, 298 K)

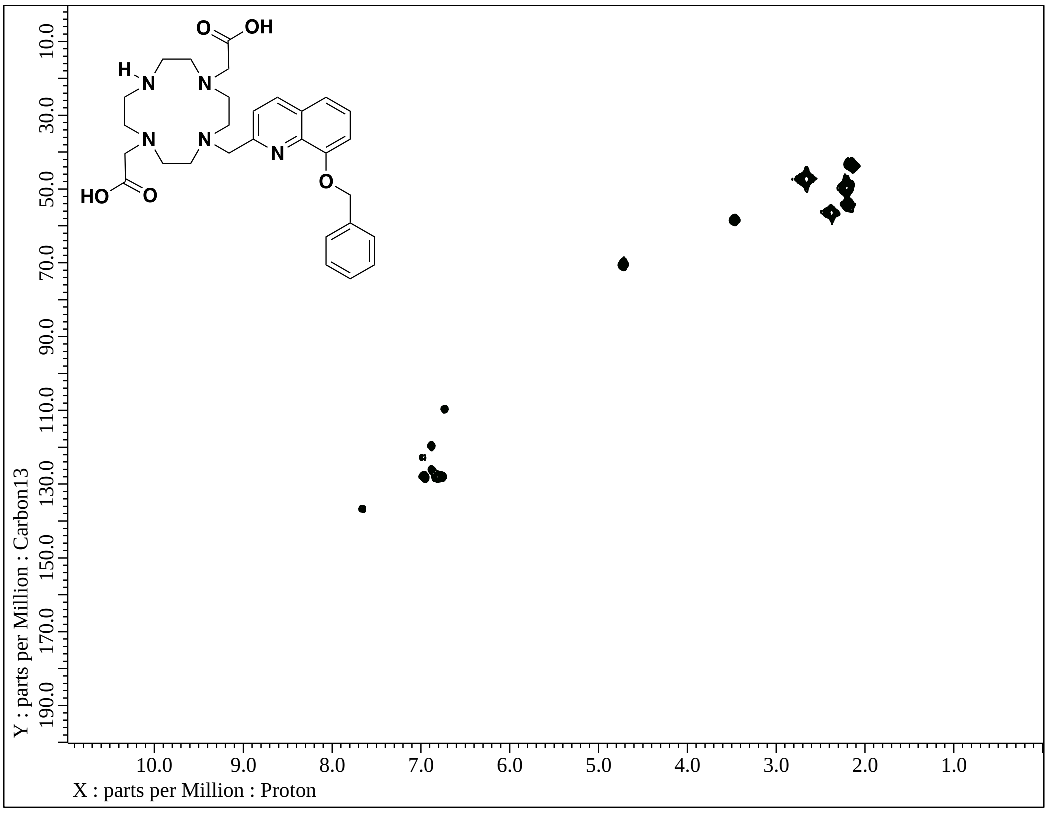

HMBC (400 MHz, methanol- d_4, 298 K)

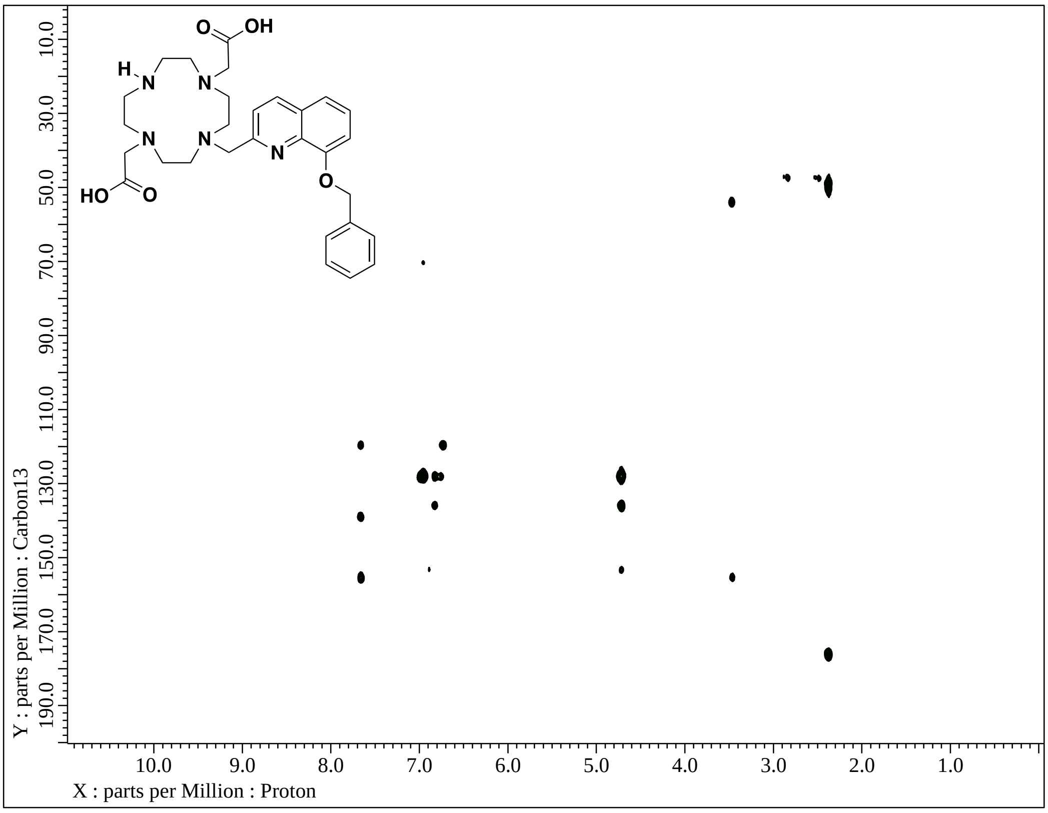

8-((3-Iodobenzyl)oxy)quinoline-2-carbaldehyde (7)

^{1}H NMR (400 MHz, $CDCl_3$, 298 K)

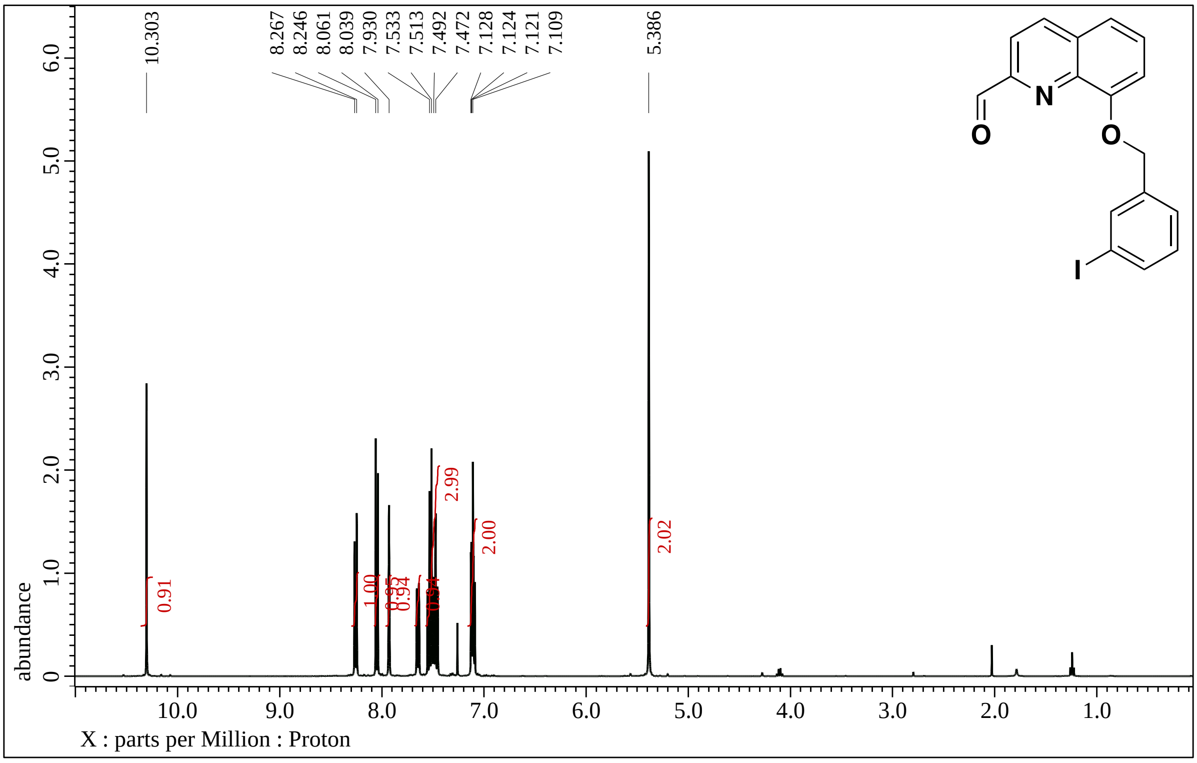

^{13}C NMR (100 MHz, $CDCl_3$, 298 K)

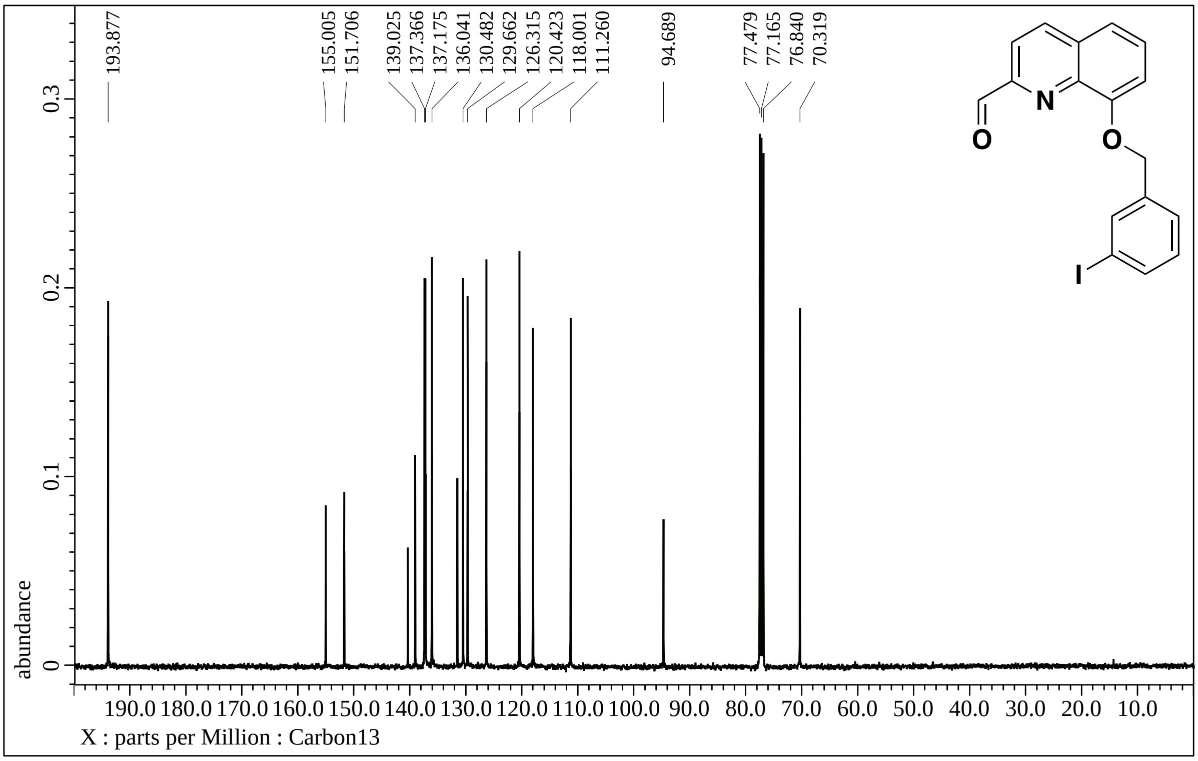

COSY (400 MHz, $CDCl_3$, 298 K) displayed range: 5.0 ppm – 10.5 ppm

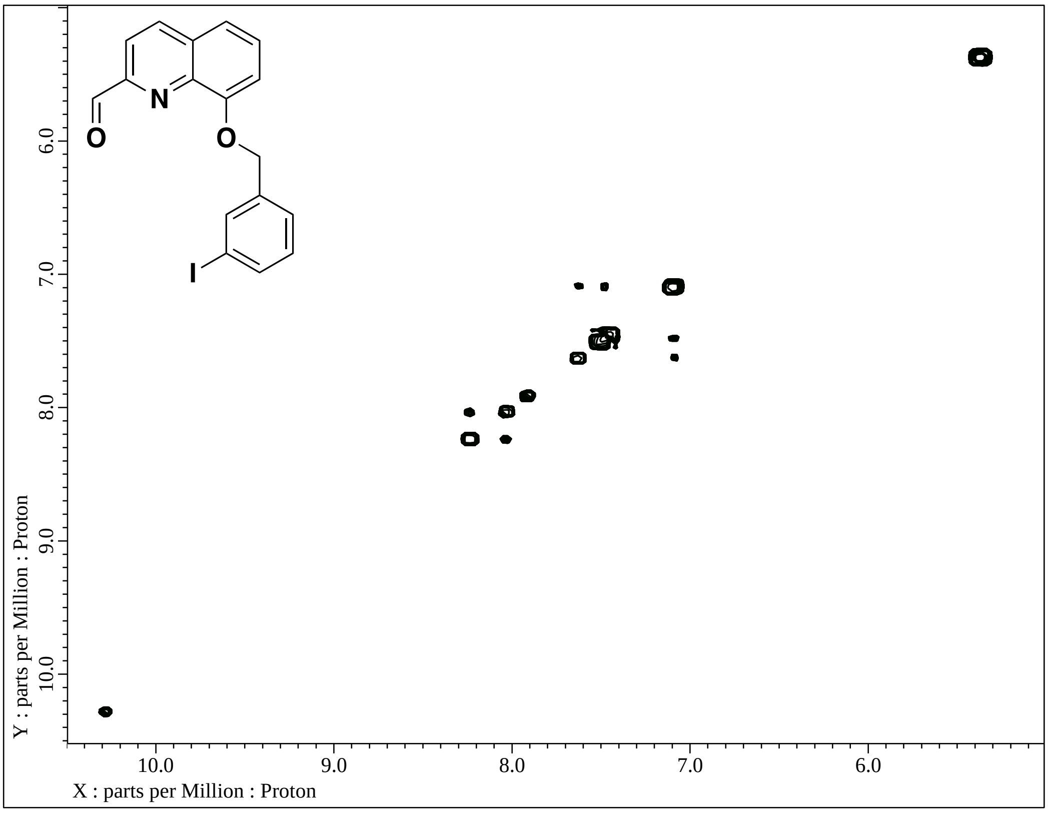

HSQC (400 MHz, $CDCl_3$, 298 K)

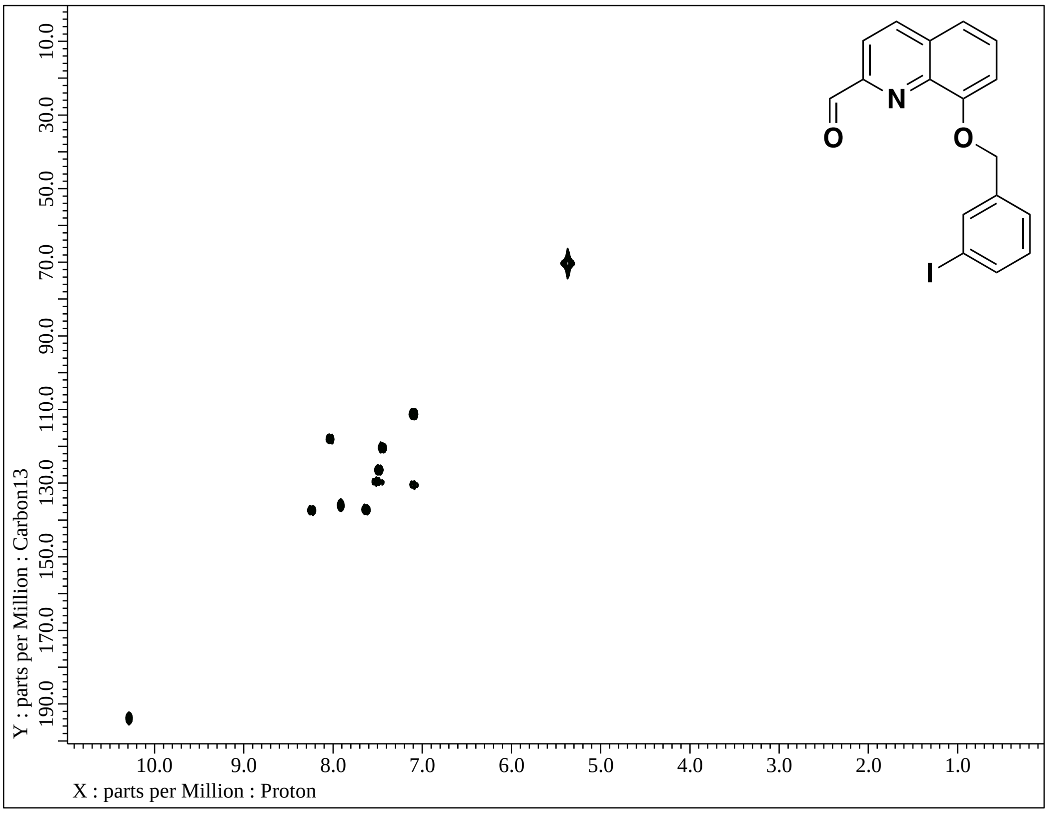

HMBC (400 MHz, $CDCl_3$, 298 K)

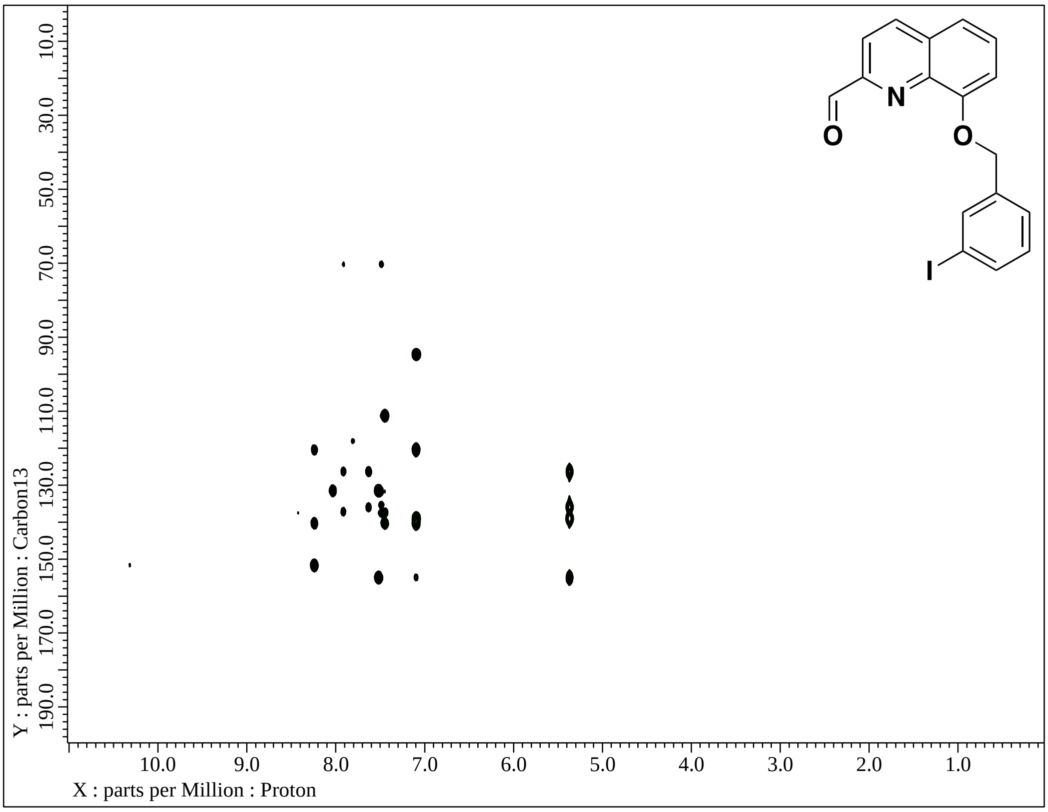

(8-((3-Iodobenzyl)oxy)quinolin-2-yl)methanol (8)

^{1}H NMR (400 MHz, $CDCl_3$, 298 K)

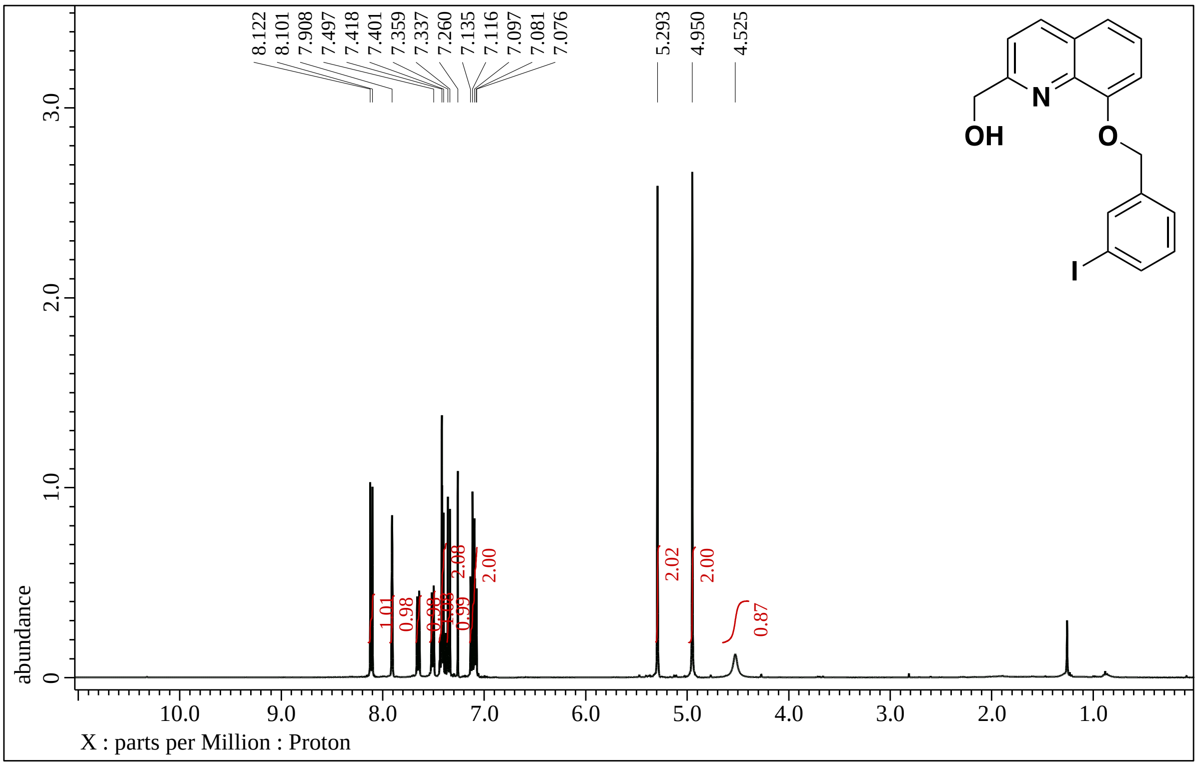

^{13}C NMR (100 MHz, $CDCl_3$, 298 K)

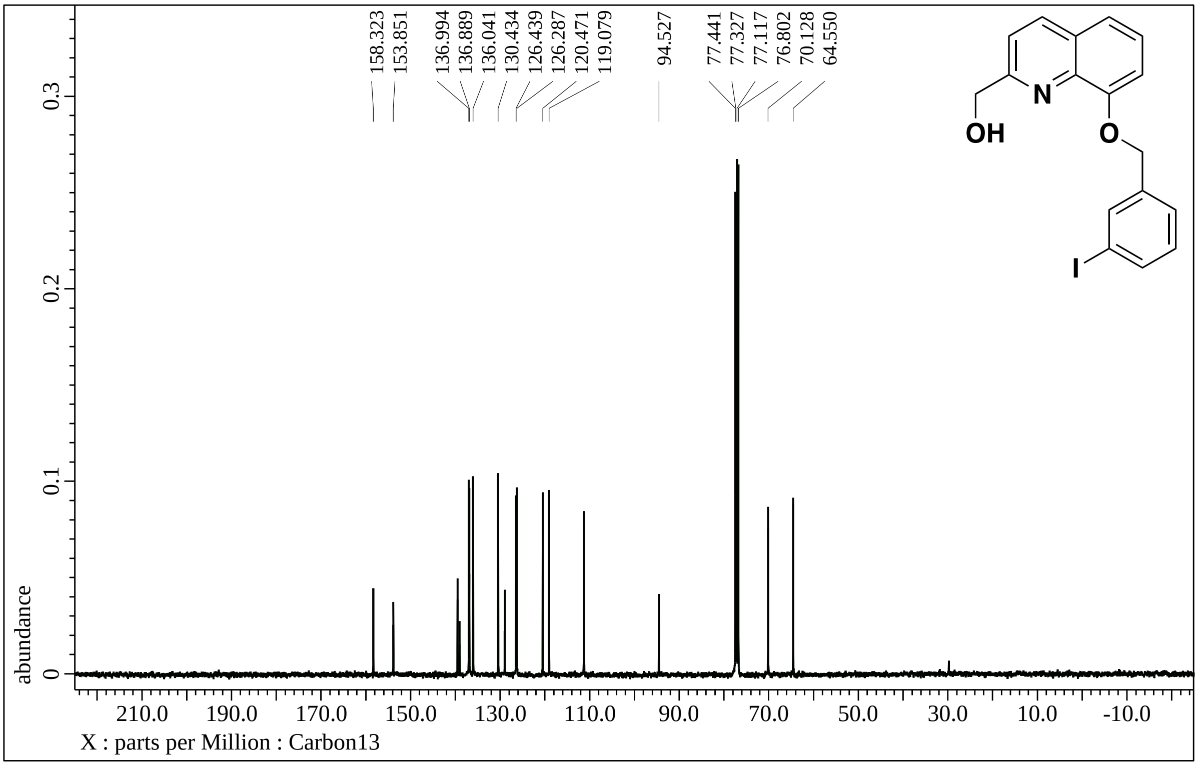

COSY (400 MHz, $CDCl_3$, 298 K) displayed range 4.5 ppm – 8.5 ppm

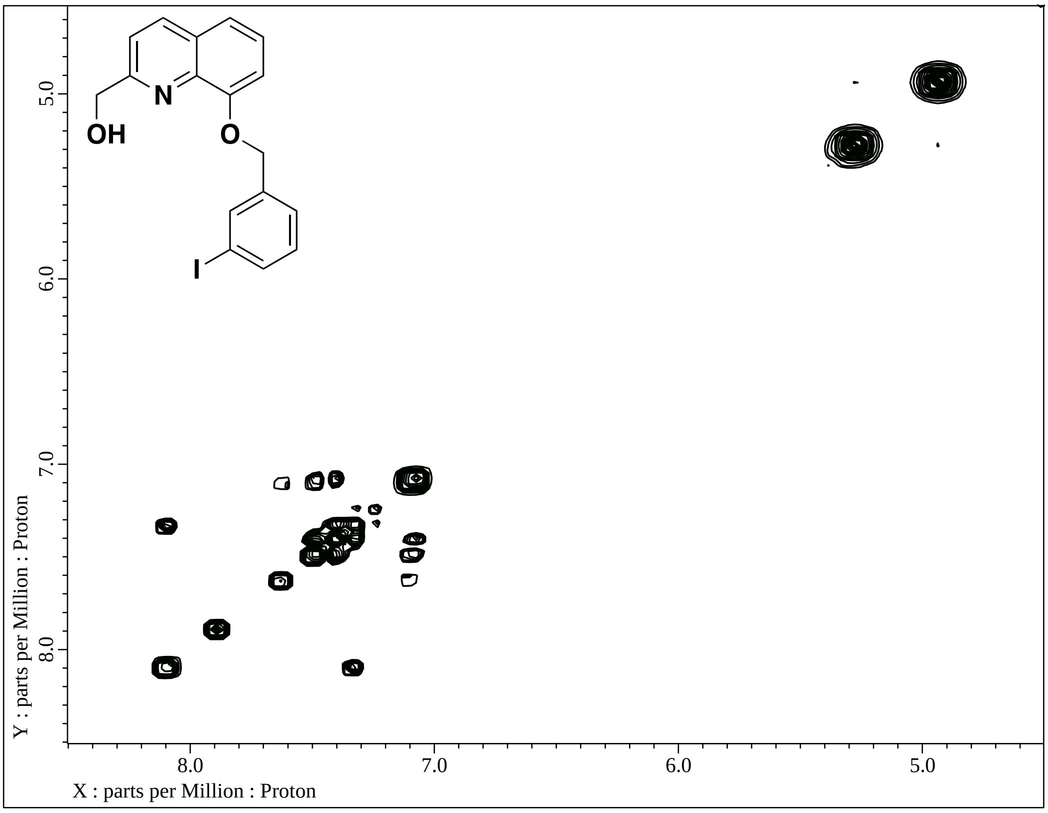

HSQC (400 MHz, $CDCl_3$, 298 K)

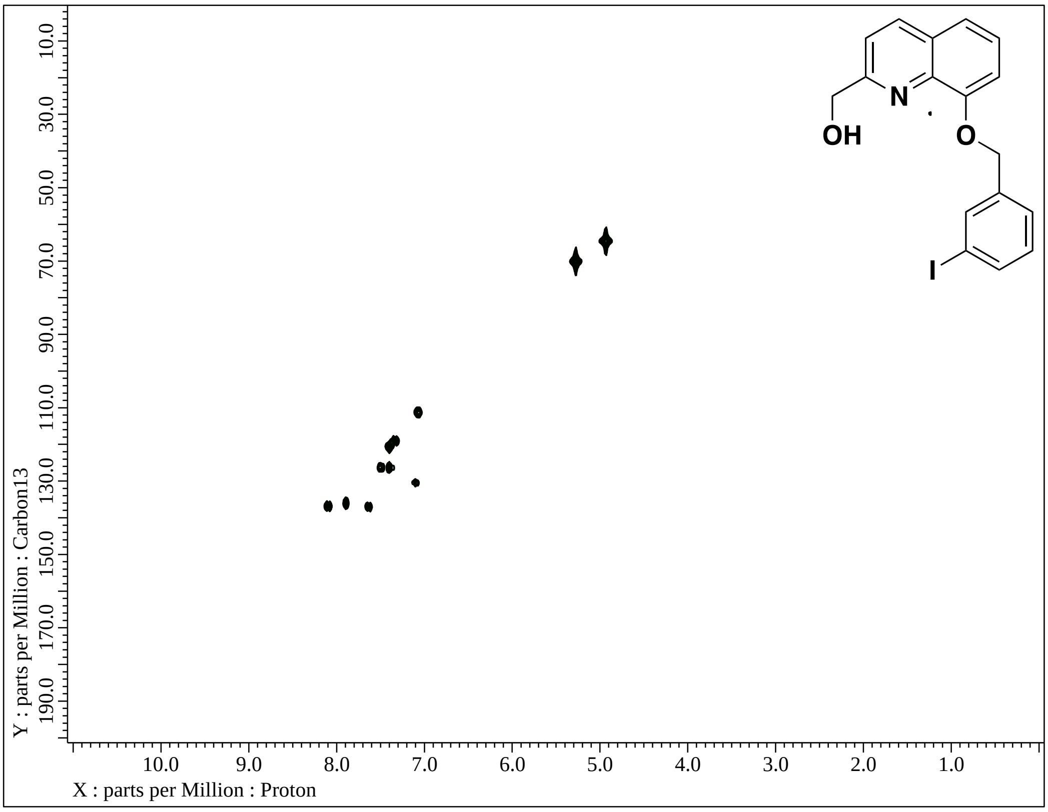

HMBC (400 MHz, $CDCl_3$, 298 K)

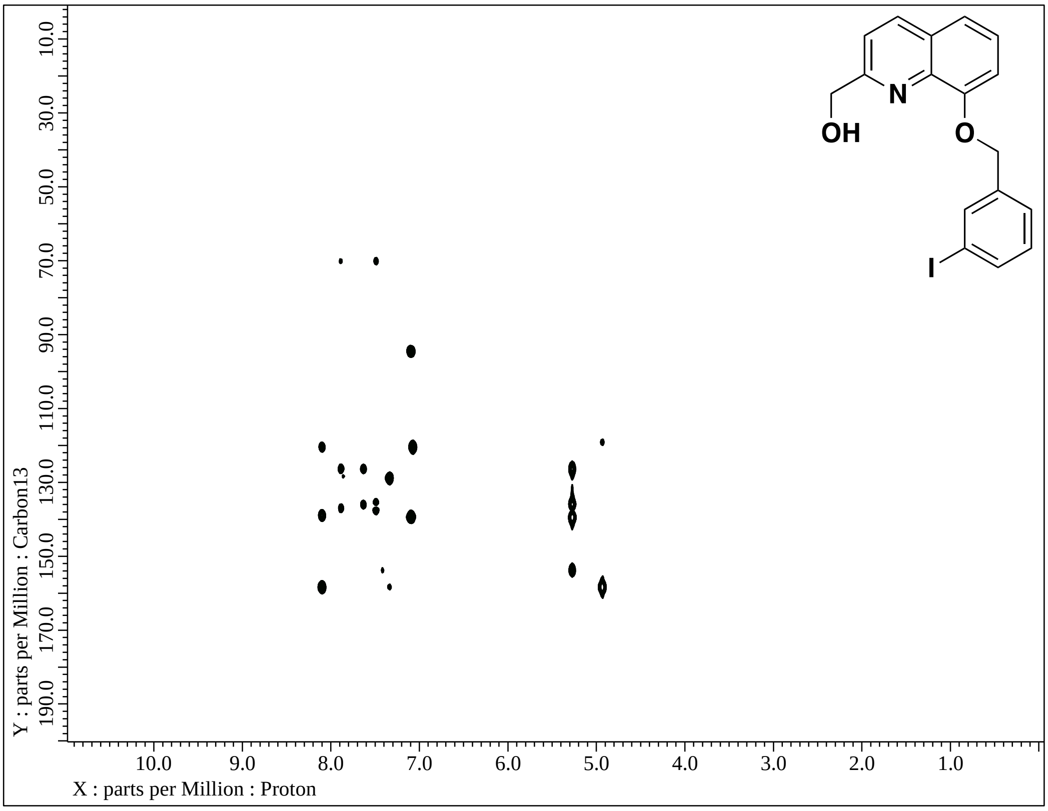

(8-((3-Iodobenzyl)oxy)quinolin-2-yl)methyl methanesulfonate (9)

^{1}H NMR (400 MHz, $CDCl_3$, 298 K)

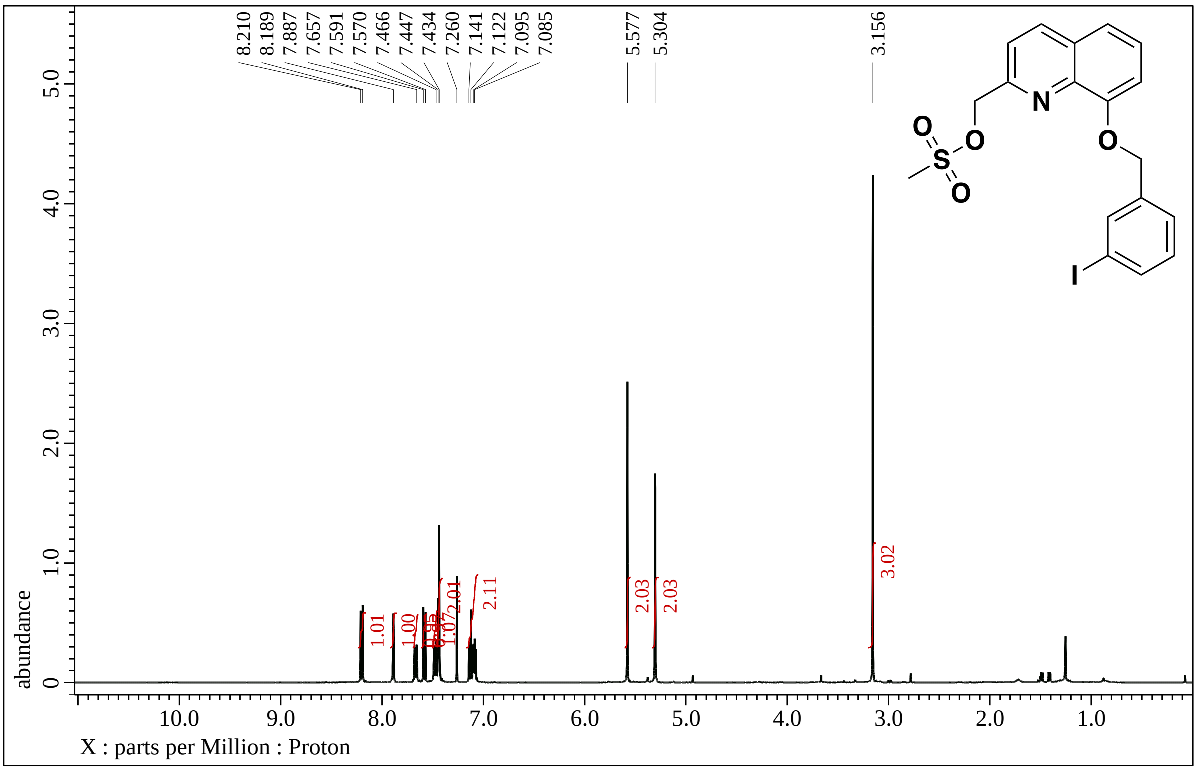

^{13}C NMR (100 MHz, $CDCl_3$, 298 K)

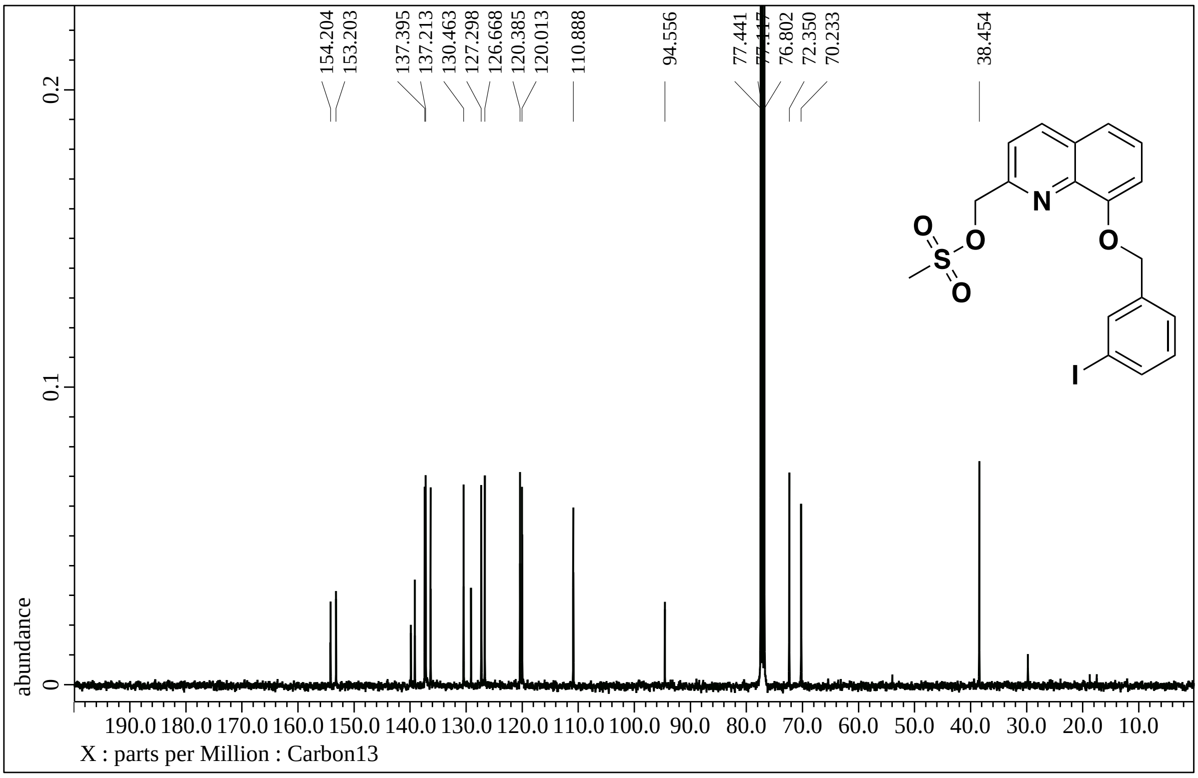

COSY (400 MHz, $CDCl_3$, 298 K) displayed range 2.5 ppm – 8.5 ppm

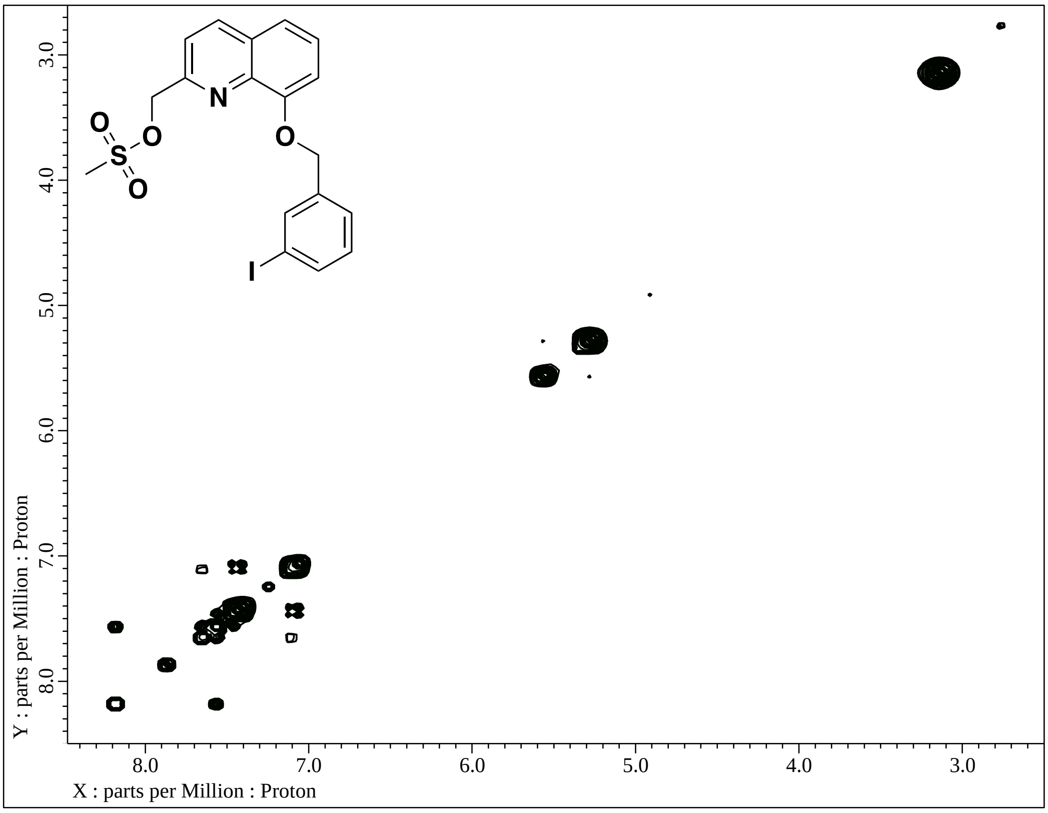

HSQC (400 MHz, $CDCl_3$, 298 K)

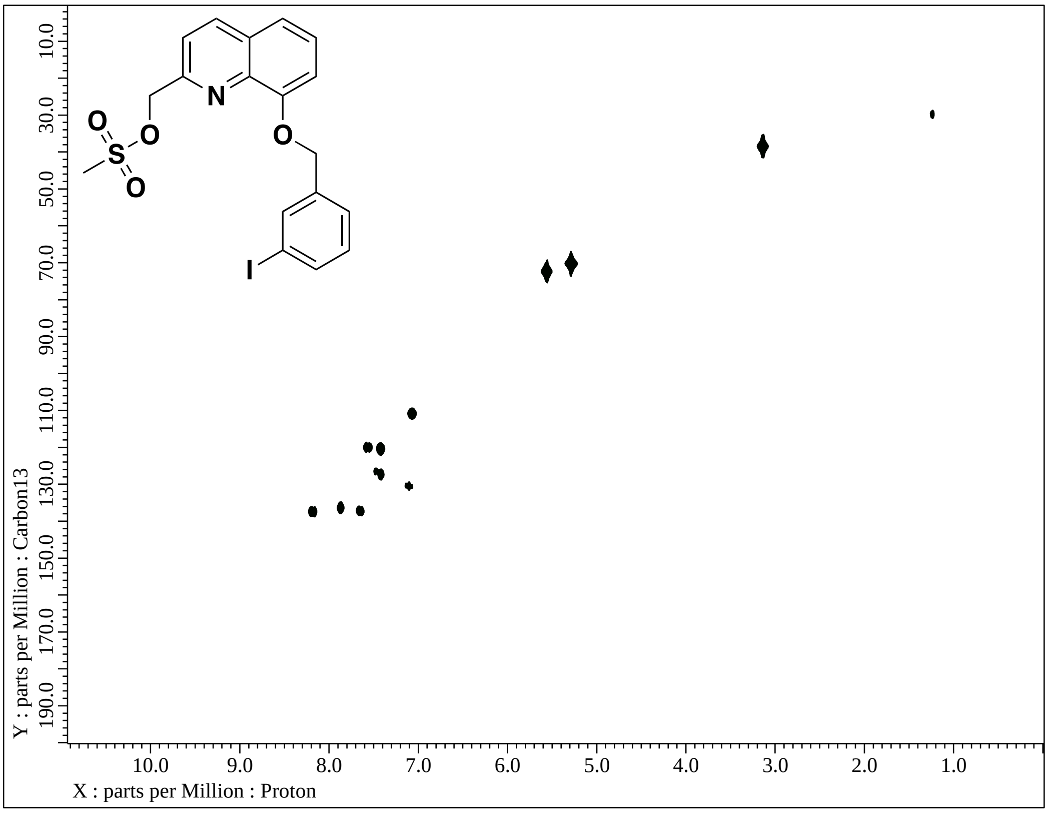

HMBC (400 MHz, $CDCl_3$, 298 K)

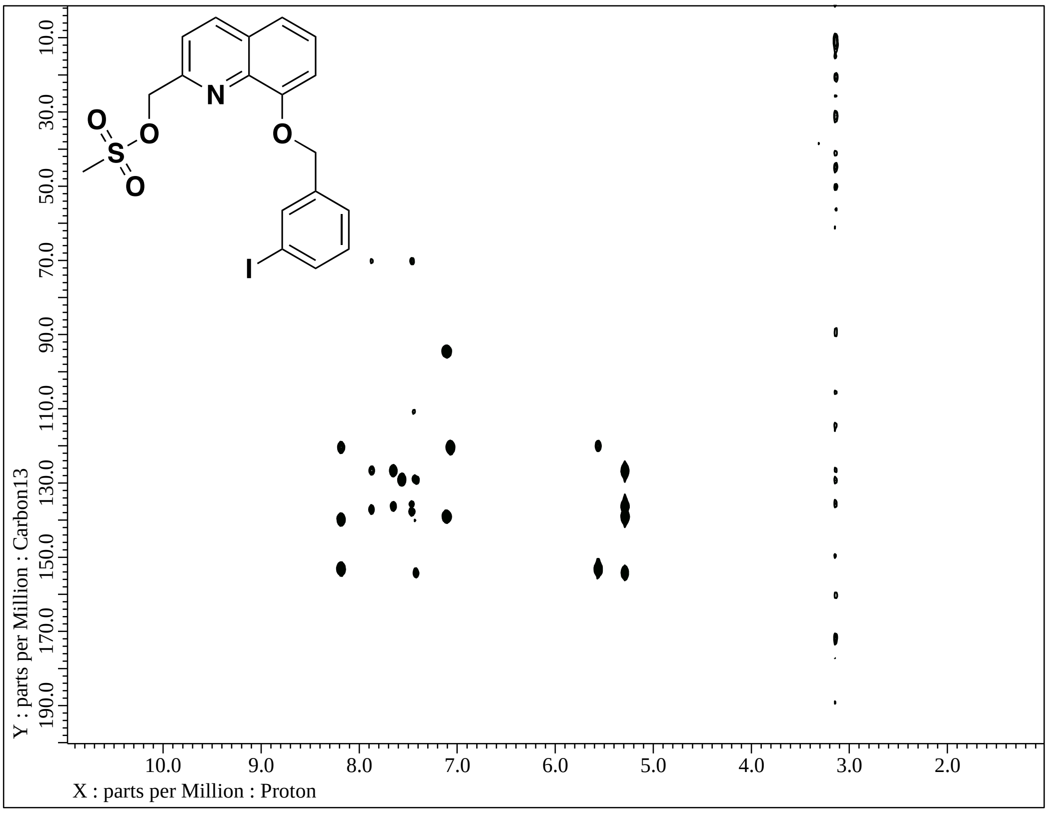

2,2'-(4-((8-((3-Iodobenzyl)oxy)quinolin-2-yl)methyl)-1,4,7,10-tetraazacyclododecane-1,7-diyl)diacetic acid (10)

^{1}H NMR (400 MHz, $CDCl_3$, 298 K)

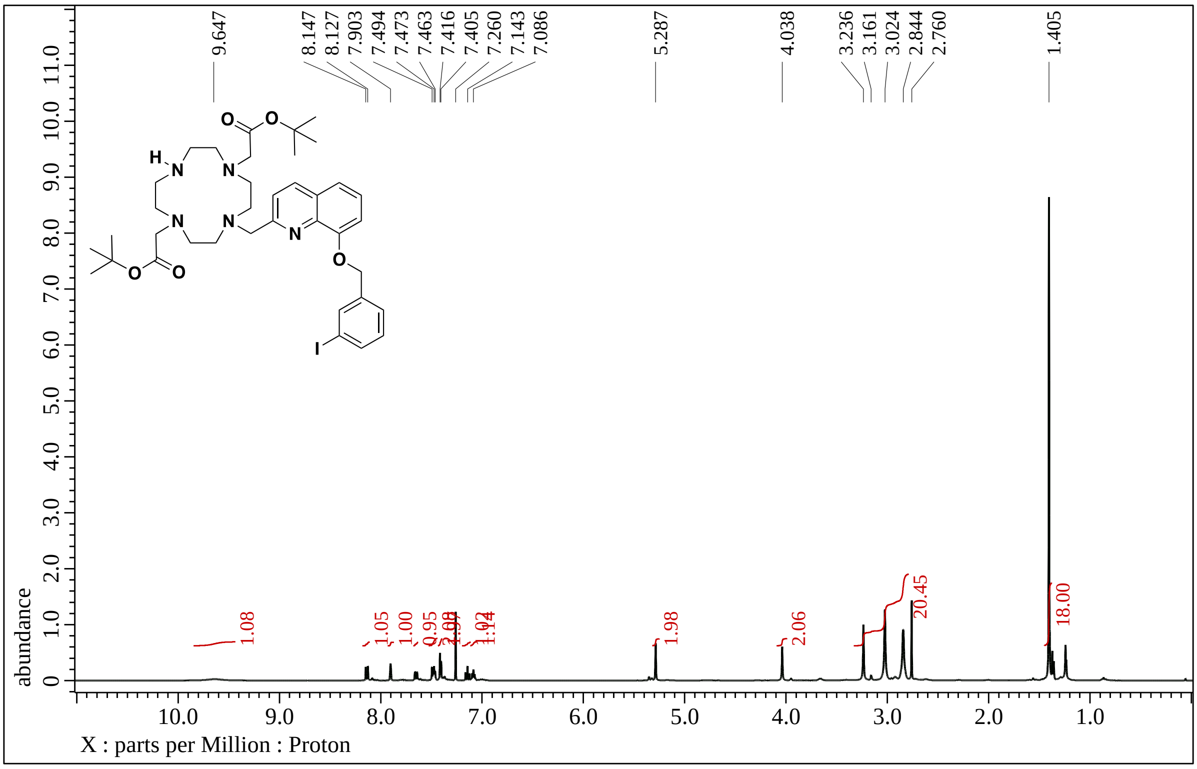

^{13}C NMR (100 MHz, $CDCl_3$, 298 K)

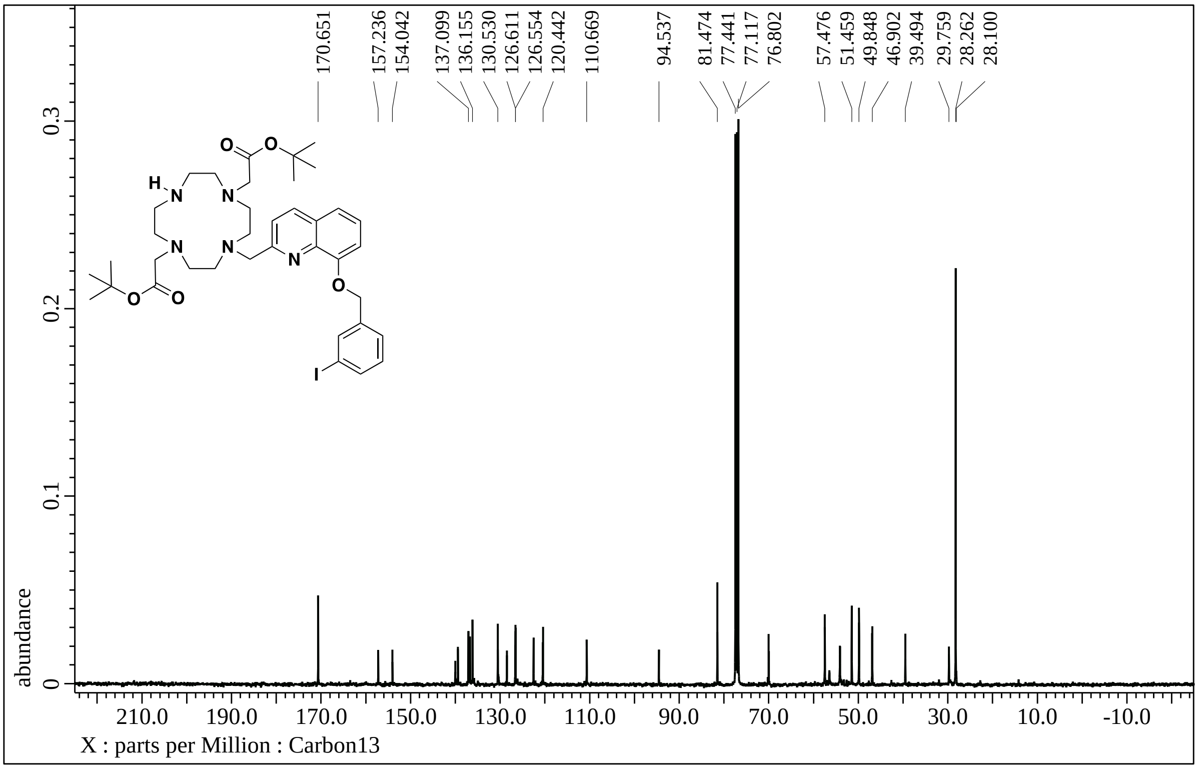

COSY (400 MHz, $CDCl_3$, 298 K) displayed range 1.0 ppm – 9.0 ppm

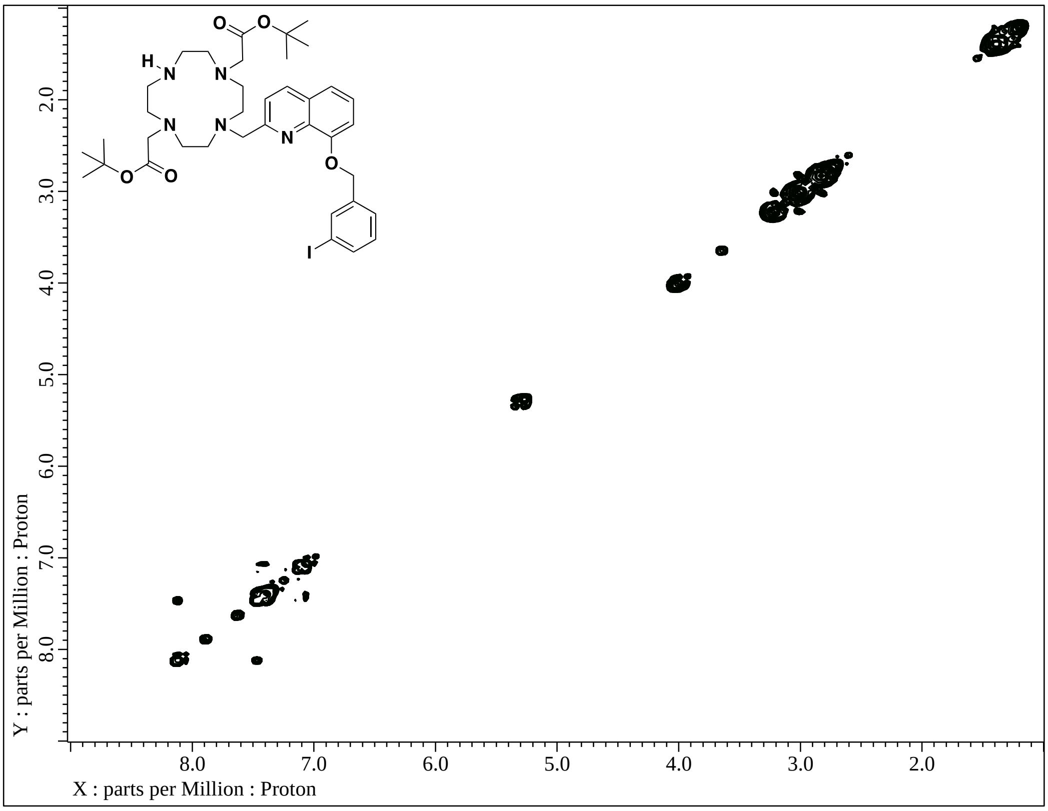

HSQC (400 MHz, $CDCl_3$, 298 K)

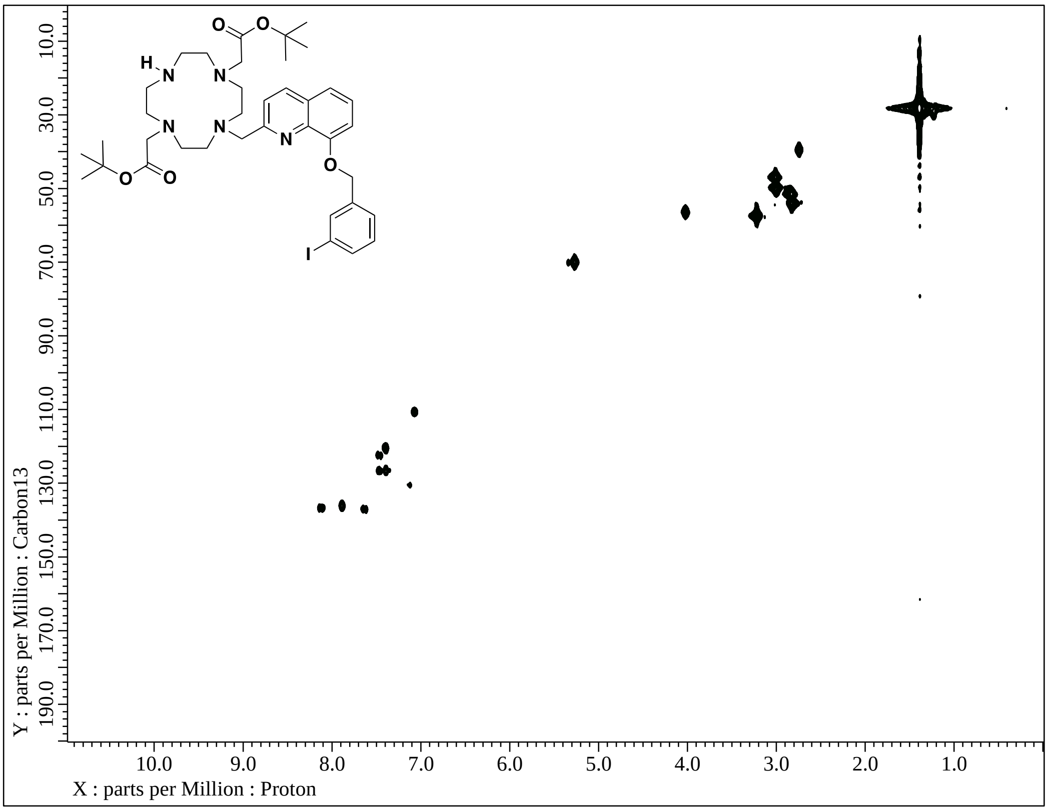

HMBC (400 MHz, $CDCl_3$, 298 K)

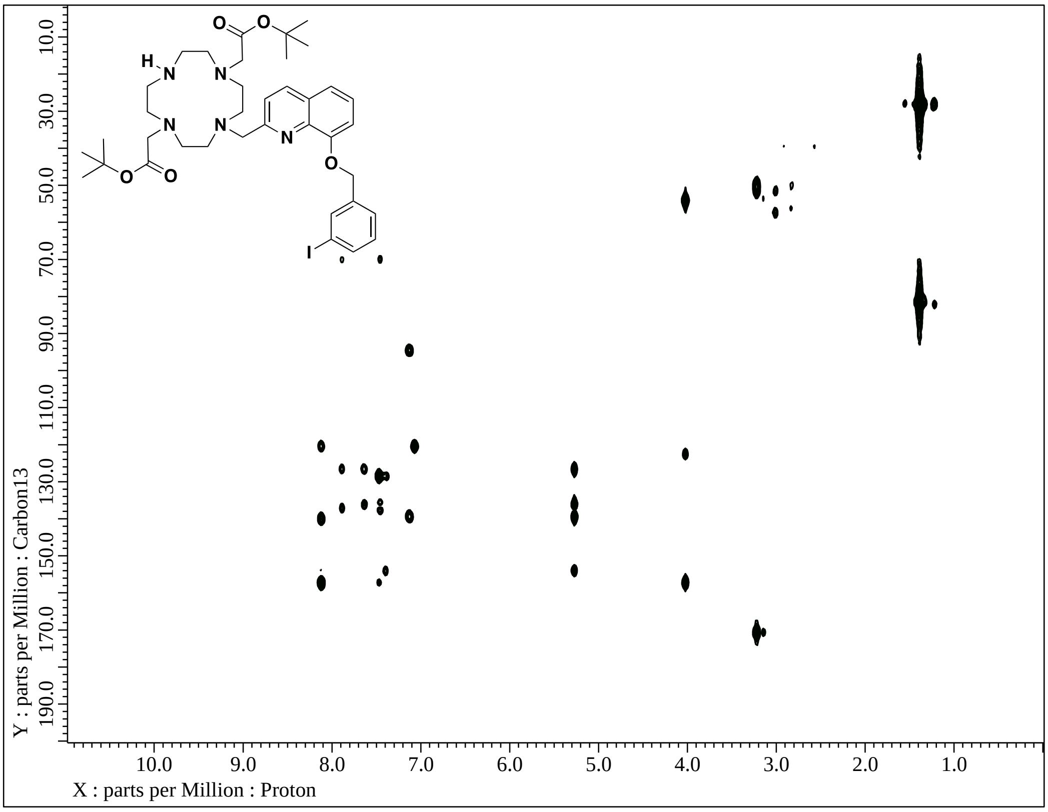

2,2'-(4-((8-((3-Iodobenzyl)oxy)quinolin-2-yl)methyl)-1,4,7,10-tetraazacyclododecane-1,7-diyl)diacetic acid (11)

^{1}H NMR (400 MHz, methanol- d_4, 298 K) [* solvent signals]

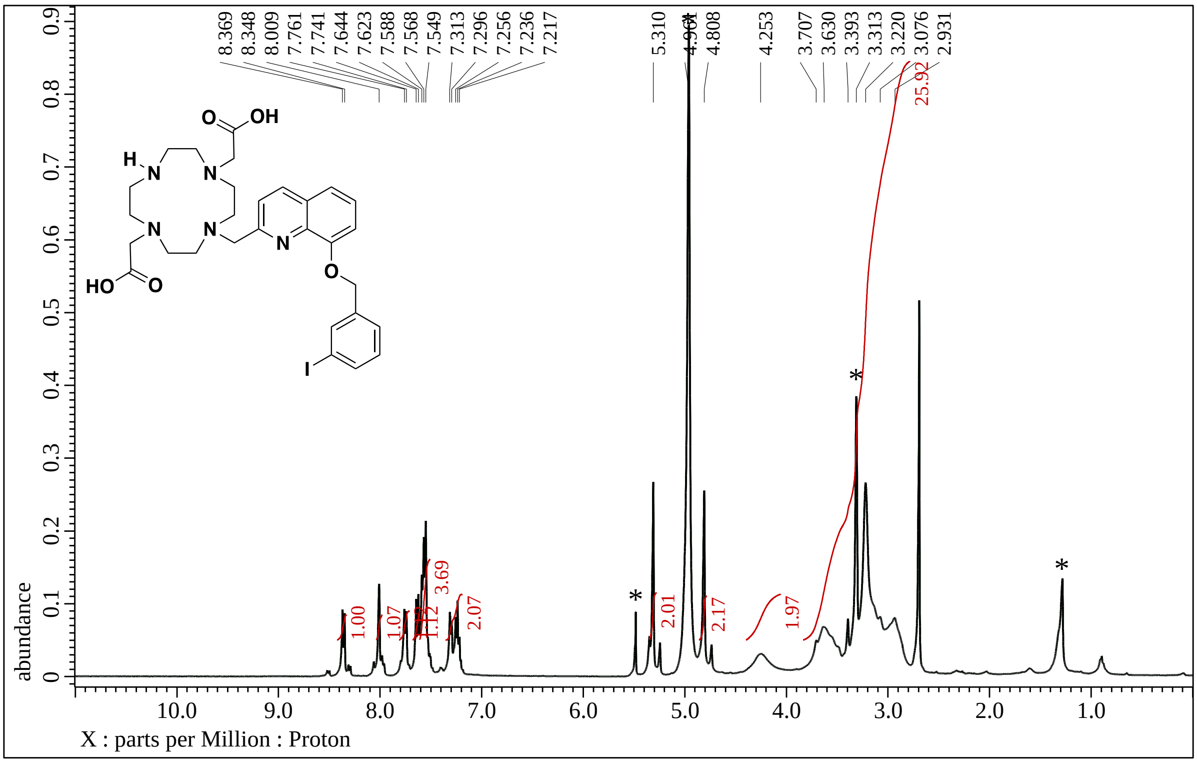

^{13}C NMR (100 MHz, methanol- d_4, 298 K)

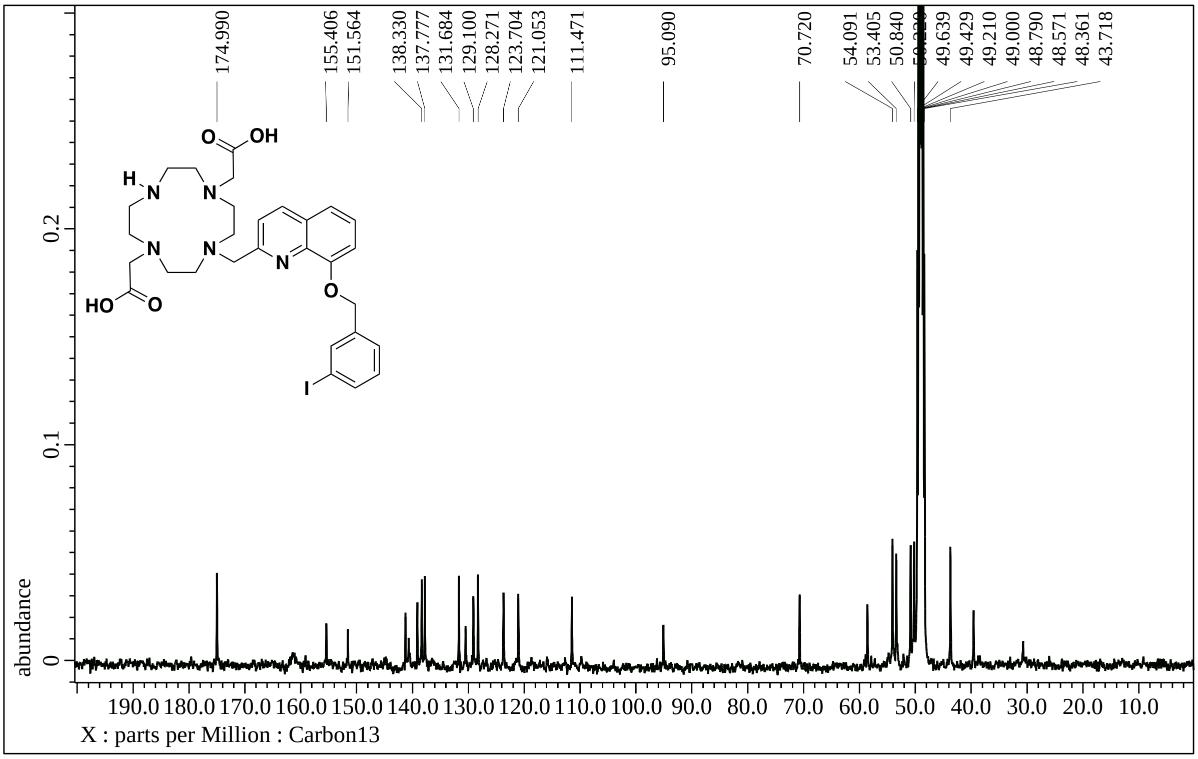

COSY (400 MHz, methanol- d_4, 298 K) displayed range 1.0 ppm – 9.0 ppm

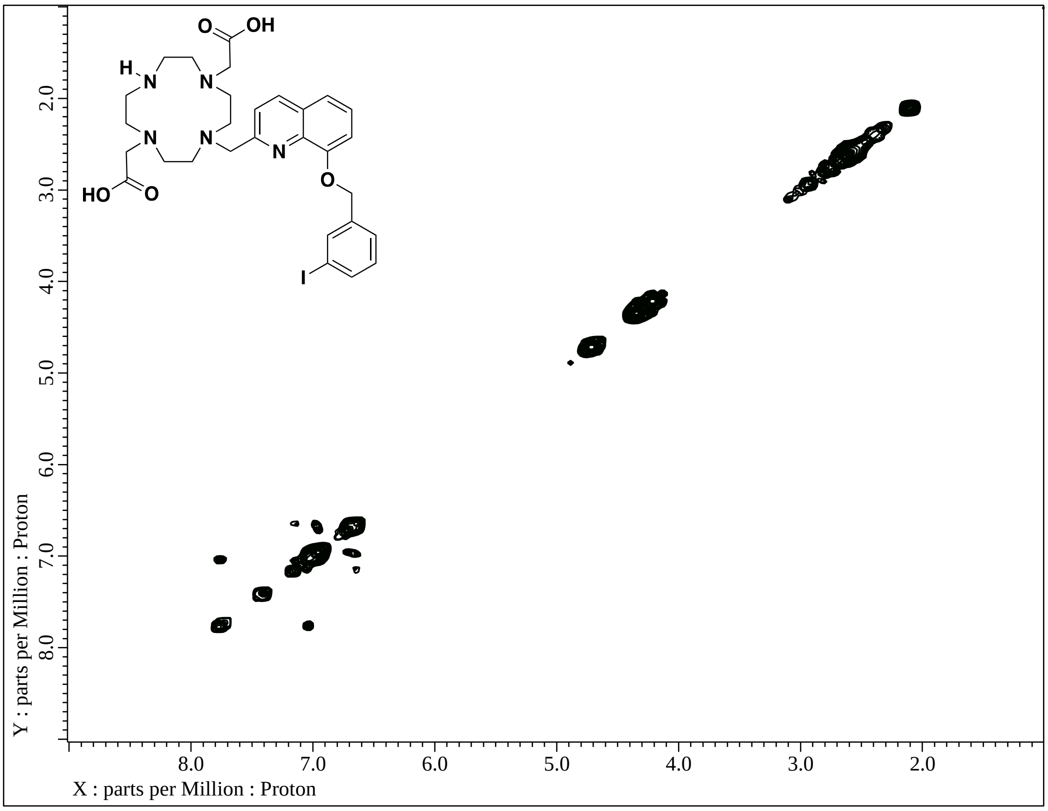

HSQC (400 MHz, methanol- d_4, 298 K)

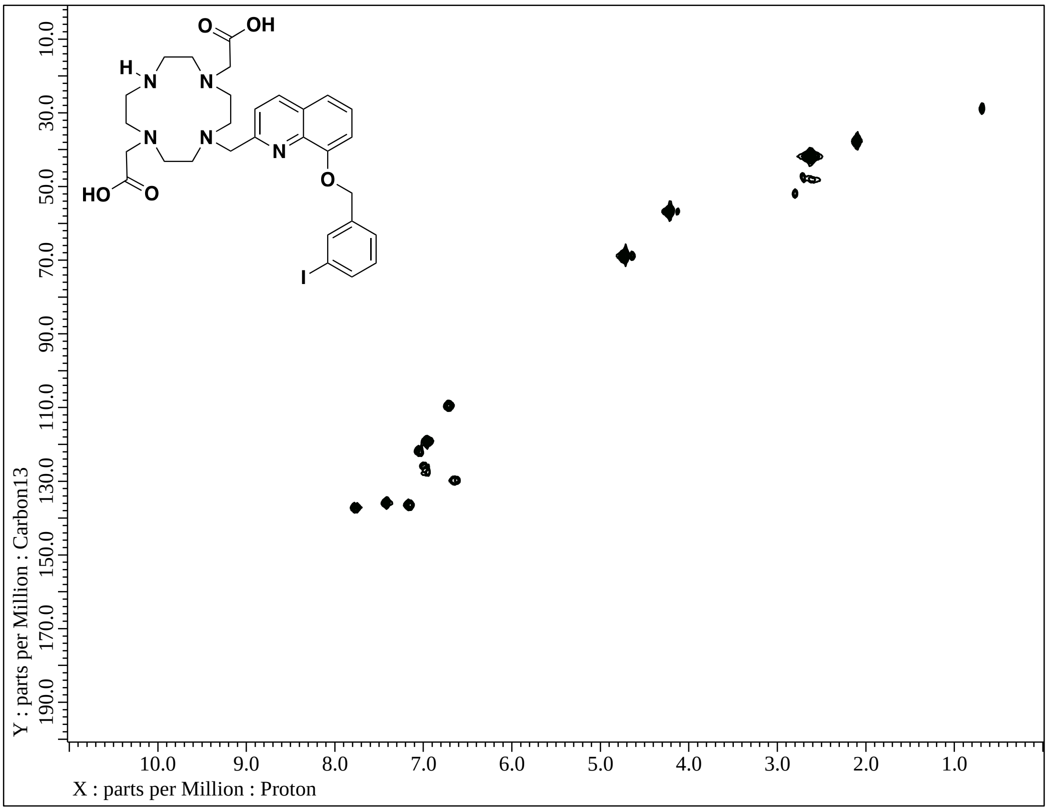

HMBC (400 MHz, methanol- d_4, 298 K)

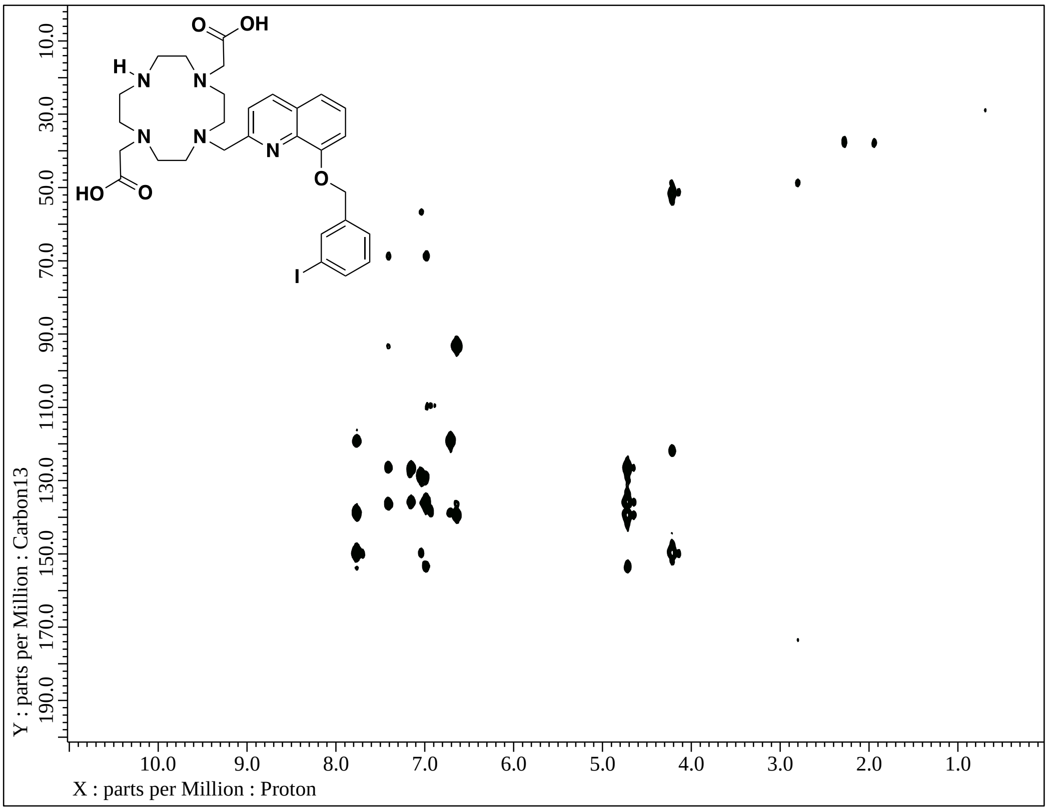

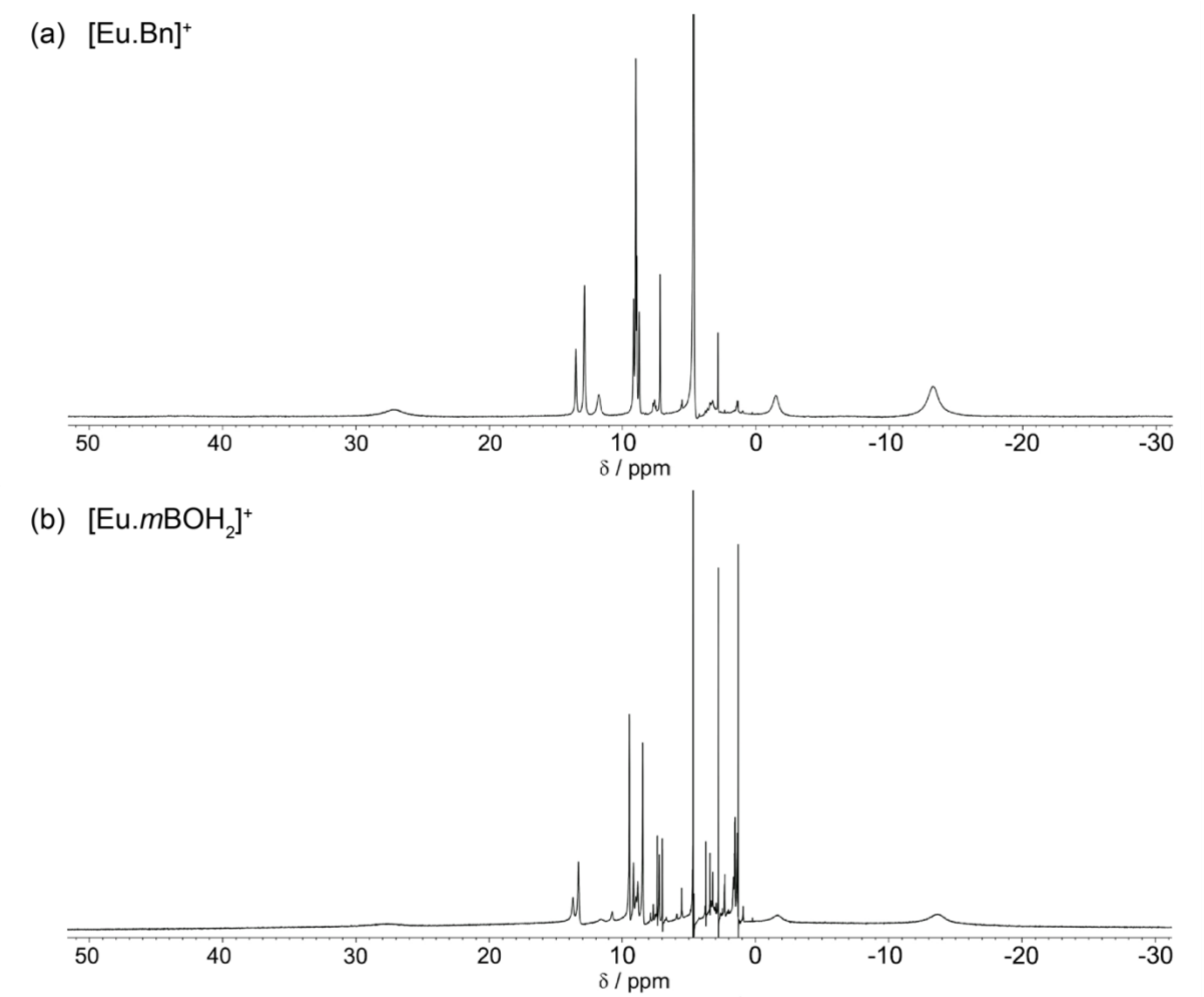

Figure S20. ^{1}H NMR spectra (500 MHz, D_2O) of (a) [**Eu.Bn**]$^{+}$**,** (b) [**Eu.*m*BOH$_2$**]$^{+}$ recorded at pD 7.4 and 50 °C. ^{1}H NMR spectra recorded at room temperature resulted in significant line broadening attributed to exchange between conformational isomers.

References

[1] K. Suzuki, A. Kobayashi, S. Kaneko, K. Takehira, T. Yoshihara, H. Ishida, Y. Shiina, S. Oishi, S. Tobita, *Phys. Chem. Chem. Phys.* **2009**, *11*, 9850-9860.

[2] A. Beeby, I. M. Clarkson, R. S. Dickins, S. Faulkner, D. Parker, L. Royle, A. S. de Sousa, J. A. Gareth Williams, M. Woods, *J. Chem. Soc., Perkin Trans. 2* **1999**, *0*, 493-504.

[3] N. Mardirossian, M. Head-Gordon, *J. Chem. Phys.* **2016**, *144*, 214110.

[4] P. C. Hariharan, J. A. Pople, *Theor. Chim. Acta* **1973**, *28*, 213-222.

[5] M. Dolg, H. Stoll, H. Preuss, *J. Chem. Phys.* **1989**, *90*, 1730-1734.

[6] V. Barone, M. Cossi, *J. Phys. Chem. A* **1998**, *102*, 1995-2001.

[7] E. Epifanovsky, A. T. Gilbert, X. Feng, J. Lee, Y. Mao, N. Mardirossian, P. Pokhilko, A. F. White, M. P. Coons, A. L. Dempwolff, e. al., *J. Chem. Phys.* **2021**, *155*, 084801.

[8] S. H. Hewitt, G. Macey, R. Mailhot, M. R. J. Elsegood, F. Duarte, A. M. Kenwright, S. J. Butler, *Chem. Sci.* **2020**, *11*, 3619-3628.

[9] J. Tian, X. Yan, H. Yang, F. Tian, *RSC Adv.* **2015**, *5*, 107012-107019.
